# Blood Immunosenescence Signatures Reflecting Age, Frailty and Tumor Immune Infiltrate in Patients with Early Luminal Breast Cancer

**DOI:** 10.3390/cancers13092185

**Published:** 2021-05-02

**Authors:** Lieze Berben, Asier Antoranz, Cindy Kenis, Ann Smeets, Hanne Vos, Patrick Neven, Giuseppe Floris, Hans Wildiers, Sigrid Hatse

**Affiliations:** 1 Laboratory of Experimental Oncology, Department of Oncology, KU Leuven, 3000 Leuven, Belgium; lieze.berben@kuleuven.be (L.B.); hans.wildiers@uzleuven.be (H.W.); 2 Laboratory of Translational Cell and Tissue Research, Department of Imaging and Pathology, KU Leuven, 3000 Leuven, Belgium; asier.antoranzmartinez@kuleuven.be (A.A.); giuseppe.floris@uzleuven.be (G.F.); 3 Department of General Medical Oncology and Geriatric Medicine, University Hospitals Leuven, 3000 Leuven, Belgium; cindy.kenis@uzleuven.be; 4 Department of Public Health and Primary Care, Academic Centre for Nursing and Midwifery, KU Leuven—University of Leuven, 3000 Leuven, Belgium; 5 Department of Surgical Oncology, KU Leuven, University Hospitals Leuven, 3000 Leuven, Belgium; ann.smeets@uzleuven.be (A.S.); hanne.vos@kuleuven.be (H.V.); 6 Department of Gynaecology and Obstetrics, University Hospitals Leuven, 3000 Leuven, Belgium; patrick.neven@kuleuven.be; 7 Department of Pathology, University Hospitals Leuven, 3000 Leuven, Belgium

**Keywords:** breast cancer, blood biomarkers, tumor immune infiltrate, aging, frailty

## Abstract

**Simple Summary:**

Treating older patients with (breast) cancer is a major challenge. On the one hand, older persons are more vulnerable to side effects of therapy, and over-treatment should be avoided. On the other hand, under-treatment (which is common in the elderly) can lead to worse survival and quality of life as well. Benefits of therapy and risk of (sometimes life threatening) toxicity should be carefully balanced. There is an urgent need for robust markers that reflect the body’s biological age and could aid in outlining optimal individual treatment regimens. Here we investigated whether age/frailty and characteristics of the tumor immune infiltrate are mirrored in specific blood biomarker combinations. Several three-biomarker panels were able to categorize patients quite efficiently, especially in terms of their clinical frailty status.

**Abstract:**

Background: Immune/senescence-related host factors play a pivotal role in numerous biological and pathological process like aging, frailty and cancer. The assessment of these host factors via robust, non-invasive, and easy-to-measure blood biomarkers could improve insights in these processes. Here, we investigated in a series of breast cancer patients in which way single circulating biomarkers or biomarker panels relate to chronological age, frailty status, and tumor-associated inflammatory microenvironment. Methods: An extensive panel of blood immune/senescence markers and the tumor immune infiltrate was studied in young, middle-aged, and old patients with an early invasive hormone-sensitive, HER2-negative breast cancer. In the old group, clinical frailty was estimated via the G8-scores. Results: Several three-blood biomarker panels proved to be able to separate old chronological age from young age very efficiently. Clinically more important, several three-blood biomarker panels were strongly associated with clinical frailty. Performance of blood biomarker panels for prediction of the tumor immune infiltrate was lower. Conclusion: Immune/senescence blood biomarker panels strongly correlate with chronological age, and clinically more importantly with frailty status in early breast cancer patients. They require further investigation on their ability to provide a more complete picture on clinical frailty status and direct personalized therapy in older persons.

## 1. Introduction

Host factors can highly affect several cancer-related aspects, such as tumorigenesis, prognosis, treatment decisions, and therapy response [1,2,3,4,5,6,7,8,9,10]. Life expectancy has increased significantly over the past years and as most epithelial cancers are considered age-related diseases, aging is an important element to take into consideration in clinical oncology [8,11]. Moreover, the general health status and frailty level of older patients might be equally or even more important [6,12]. In addition, the immune system is a crucial defense mechanism against diseases, including cancer. Given the recent clinical successes of immunotherapy, immune profiling has gained a large amount of interest in the cancer setting [2,13,14]. In particular, inflammation is highly linked with cancer but also with aging [9,10]. Robust, non-invasive and easy-to-measure biomarkers may provide important insights into the patient’s biological age and immune status and eventually could aid with oncological decision-making. Here, we investigated in patients with breast cancer how blood immunosenescence biomarkers or biomarker panels correlate with a patient’s chronological age, frailty status or even tumor-related immune microenvironment.

Frailty levels of patients can be estimated via comprehensive geriatric assessment (CGA). However, there are no precise criteria to define frailty, and CGA requires time and trained personnel, withholding widespread implementation of CGA in routine clinical practice [6,7]. Several screening tools have been developed, such as the ‘Geriatric 8 (G8)’, for oncological patients to identify patients in need of CGA [7,15]. G8 by itself already has strong prognostic impact in older patients with cancer. Nevertheless, these clinical tools are imprecise measures of frailty, and it might be interesting to evaluate additional features of frailty, such as a panel of circulating biomarkers. Like aging, frailty is associated with profound biological alterations. Increased levels of inflammatory mediators (e.g., interleukin (IL)-6, tumor necrosis factor alpha (TNF-α)) have been observed in the blood [16], as well as differential expression of circulating microRNAs (miRNAs) (e.g., miR-92a, miR-326,…) [17], alterations in immune cell populations (e.g., loss of CD28 expression and/or increased expression of CD57 on T-cells) [18] and increased expression of *P16^INK4a^* in T-cells [19].

The immune system plays an eminent yet complex role in tumor development and progression. A determining factor is the balance between anti- and pro-tumor immunity. T-lymphocytes, particularly cytotoxic T-cells (CD8^+^ T-cells), mainly exert anti-tumor activities, whereas myeloid derived suppressor cells promote tumorigenesis via their immunosuppressive functions [1,2]. Furthermore, the tumor immune infiltrate has been linked to prognosis and response to therapy in different solid tumor types, including breast tumors [2,20,21]. In breast cancer (BC), the tumor immune infiltrate is very heterogeneous and marked differences can be observed between BC subtypes [3]. The prognostic and predictive value of tumor infiltrating lymphocytes (TILs) has mainly been established in triple negative BC (TNBC) and human epidermal growth factor receptor 2-positive (HER2^+^) BC. Noteworthy, high TIL levels are associated with a more favorable prognosis in TNBC and HER2^+^ BC whereas being a poor prognostic factor in luminal (hormone-sensitive) BC and also in special subtypes of breast carcinomas that are usually linked to ER expression [3,4,22,23]. However, the role of TILs in the luminal BC subtypes is not fully elucidated yet. As of now, the tumor immune infiltrate can only be assessed when tumor tissue is available, which is not always the case (e.g., if surgery is not performed for any reason). In these situations it might be clinically relevant to consider circulating biomarkers that could reflect the status of the tumor immune infiltrate using minimally invasive approaches. Aging (and frailty) is associated with a decay of immune function, referred to as ‘immunosenescence’. This is characterized by shifts within immune cell populations accompanied by functional changes and is linked to an increased low-grade inflammation. Thus, aging and/or frailty might have a substantial impact on the tumor immune infiltrate [9,10].

Recently we performed a thorough investigation of the immune landscape in tumor and blood of patients with luminal BC [24,25]. Numerous significant age- and frailty-related changes in the blood immune profile and the tumor immune infiltrate were observed. Now, we want to investigate whether age/frailty and characteristics of the tumor immune infiltrate are mirrored in specific blood biomarker combinations. To identify such biomarker signatures, we have applied extensive bioinformatics analyses on the dataset of the above referenced study.

## 2. Materials and Methods

### 2.1. Patient Selection

Patients with an early estrogen receptor positive, HER2-negative, grade II or III invasive breast carcinoma, diagnosed on core needle biopsy and with clinical tumor size of at least 1.5 cm, planned for surgery, were included in the previously published IMAGE (IMmunity-AGE) study [24] (NCT02327572). This exploratory biomarker study included patients of 3 distinct age categories: young group (35–45 years, premenopausal), middle age group (55–65 years, postmenopausal) and old age group (≥70 years, postmenopausal). As we are mainly interested in the old age category in the present analysis, the two younger groups were merged together, creating a younger age group (35–45/55–65 years, *N* = 34) and an older age group (≥70 years, *N* = 31). Patients signed an informed consent and an extra blood sample was drawn and processed at inclusion (before surgery). Additionally, in the oldest age category (≥70 years), clinical frailty was estimated via the G8 screening tool. This enables us to perform frailty-related analysis within the old group, with a ‘fitter’ group (G8 > 14, *N* = 19) and a ‘frailer’ group (G8 ≤ 14, *N* = 10). The tumor-related analyses were performed only in case of availability of sufficient residuary tumor tissue (*N* = 62). Table 1 summarizes main patient and tumor characteristics.

### 2.2. G8 Assessment, Blood Collection, Biomarker Analysis, and Tumor Biomarker Analysis

A detailed description of the material and methods used can be found in our previous publications [24,25]. In brief, results of the widely used G8 screening tool were used as a surrogate for frailty [15,26]. G8 scores range from 17 (‘fit’) to 0 (extremely ‘frail’), patients scoring 14 or lower are considered vulnerable or frail while patients scoring higher than 14 are considered to be fit, as they have a low chance of exhibiting significant age related health problems when CGA is performed [15,27]. At inclusion a total volume of 20 mL blood was collected and was used for plasma collection, T-cell isolation and peripheral blood mononuclear cell (PBMC) isolation. Via the Human insulin-like growth factor (IGF-I) Quantikine ELISA kit (R&D Systems, Minneapolis, United States) plasma levels of IGF-1 were measured. Cytometric bead arrays (AimPlex Human Inflammation 11-plex; ImTec Diagnostics, Antwerp or custom LEGENDplex^TM^ Assay panels; BioLegend, San Diego, United States) were used to determine the plasma levels of a broad panel of cytokines [IL-1α; IL-1β; IL-6; IL-10; IL-12p70; IL-17A; IL-17F; IL-27; interferon gamma (INFγ); TNFα and transforming growth factor beta 1 (TGF-β1)), chemokines (interferon gamma-induced protein 10 (IP-10); IL-8 and monocyte chemoattractant protein 1 (MCP-1)), immune checkpoint proteins (sCD25; 4-1BB; CD86; cytotoxic T-lymphocyte-associated protein 4 (CTLA-4); programmed death-ligand 1 (PD-L1); programmed cell death protein 1 (PD-1); T-cell immunoglobulin and mucin domain-containing molecule 3 (TIM-3); lymphocyte-activation gene 3 (LAG-3); galectin (Gal-9); sCD27; PD-L2) and C-reactive protein (CRP). Additionally, levels of 20 immune related plasma miRNAs (let-7e, let-7i, miR-9, miR-17, miR-18a, miR-19a, miR-19b, miR-20a, miR-21, miR-92a, miR-125b, miR-126, miR-146a, miR-150, miR-155, miR-181a, miR-195, miR-223, miR-326 and miR-424) were quantified using miRCURY LNA miRNA Serum/Plasma Focus PCR panels (Qiagen, Hilden, Germany) and miRCURY LNA SYBR^®^ Green PCR Kit (Qiagen, Hilden, Germany). In the isolated T-cells, the expression level of *P16^INK4a^* was measured using probe-based RT-qPCR. Detailed PBMC immune subset profiling was obtained with fluorescent antibody panels using flow cytometry.

### 2.3. Tumor Immune Marker Analysis

For the final analysis, the pathological TNM staging and tumor grade were evaluated on the resection specimen. Formalin-fixed paraffin-embedded tumor tissue of the surgical resection specimen was cut at a thickness of 5 µm. On representative hematoxylin and eosin (H&E) stained tumor sections the stromal tumor infiltrating lymphocyte (sTIL) density was assessed according to published guidelines [21]. Moreover, CD3 and CD8 infiltration in different tumor regions was evaluated via immunohistochemistry (IHC) using a scoring protocol based on QuPath software as previously described [25]. We report the % of sTILs, as well as CD3 and CD8 infiltration in the whole tumor or invasive front. For sTILs, ‘low’ infiltration was defined as less than 10% sTILs, ‘intermediate’ infiltration with 10–40% sTILs and ‘high’ infiltration with more than 40% sTILs [4]. The cut offs for a low, intermediate or high CD3 or CD8 infiltration were based on IQR of the CD3 and CD8 densities: ’low infiltration’ were the tumors with a density in the lower quartile (lowest 25%); ‘high infiltration’ were the ones with a density in the upper quartile (highest 25%).

### 2.4. Statistics

Given the exploratory nature of the study, no upfront sample size was calculated and the statistical tests were performed without correcting for multiple testing. The R software (R project, Vienna, Austria) was used for the different analyses. To ensure proper comparison of the numerous biomarkers measured in different units, a z-score of each individual biomarker was computed. To evaluate statistical performance of the biomarkers, area under the curve (AUC) via receiver operating characteristic (ROC) analysis, *p*-value via Wilcox rank-sum test and log fold-change (FC), were calculated. FC is a measurement describing how much a given measure differs from a reference one and is measured as a ratio (FC = A/B). When taking the logarithm, the ratio becomes a subtraction (log FC = A − B). A positive log FC indicates that the measurement is higher than its reference while a negative measurement indicates that it is smaller. Based on their statistical performance, all biomarkers were assigned an AUC rank, *p*-value rank and a log FC score. In the final score the AUC weighted double compared to the *p*-value and log FC score. Different predictive models were trained and visualized using combinations of one, two, and three biomarkers via linear discriminant analysis (LDA). Briefly, LDA is a machine learning method that finds linear combinations of the features included in the study that separates the different classes. The resulting combination can be used as a classifier or biomarker on its own. The number of linear discriminants depends on the complexity of the model which is defined by the number of features and the number of classes used. Moreover, the performance of the blood biomarker panels complemented with age, BC parameters (tumor size, tumor grade, and lymph node involvement) or frailty status as additional variables were also evaluated. Due to the relatively small sample size and the exploratory character of the study, cross-validation was not performed. The predictive models were assigned rankings based on their accuracy and loss (L) across all the samples. Accuracy represents the number of correctly classified samples defined as (TP+TN)/(P+N), where TP: true positives, TN: true negatives, P: positive samples, N: negative samples. On the other hand, L (i.e., sum of errors made for each individual sample when comparing predicted value with true value) is a number indicating how bad the model’s prediction is for a given sample. If the prediction is perfect, then L is zero, otherwise L is bigger than 0. In our case we have used residual probabilities of misclassification for the calculation of L. LDA training focuses on finding the right set of parameters that return, on average, a low L across all samples.

## 3. Results

### 3.1. Performance of the Blood Biomarkers to Discriminate the Different Age Groups

The individual performance of each biomarker for the prediction of the correct age group can be found in Table S1. The 10 top-ranked biomarkers included miRNAs (miR-326, miR-155, miR-18a and miR-19b), T-cell *P16^INK4a^* expression, plasma cytokines (IL-1α and IL-17A), plasma Gal-9, and naive CD8^+^ cells expressing CD27 alone or in combination with CD28.

**Figure 1:**
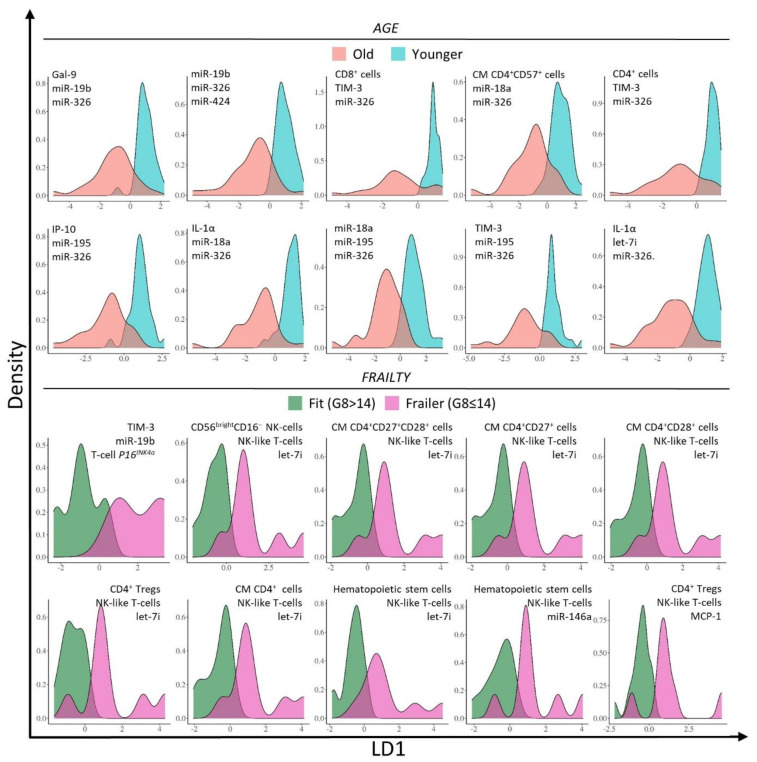
Linear discriminant analysis (LDA) density plots of the blood biomarker panels predicting age and frailty status with the LD1 coordinate on the x-axis and the density on the y-axis. The top 10 highest ranked blood biomarker panels are shown (Table 2). Old patients (≥70 years) are represented in blue, younger patients (35–45/55–65 years) in red, fit patients (G8 > 14) in green and frailer patients (G8 ≤ 14) in purple.

Next, an LDA-based algorithm was used to create two- or three-biomarker panels and compare their ability to classify patients in the correct age group. Panels consisting of 3 biomarkers generally showed the highest accuracy (i.e., % of patients correctly classified) and lowest L for allocation of patients into distinct age groups. The 10 highest ranked biomarker panels for age can be found in Table 2 (part a) and are visualized by LDA density plots in Figure 1. Several biomarker combinations with an accuracy of 90% or higher and relatively low L ranging between 12.8 and 17.4% could be identified. This performance is visualized by a fairly good separation in the density plots in Figure 1.

### 3.2. Performance of the Blood Biomarkers to Discriminate the Different Frailty Groups

The individual performance of each biomarker to predict frailty status can be found in Table S2. Apart from T-cell *P16^INK4a^* expression, all biomarkers in the top 10 were PBMC populations: NK-like T-cells; CD8^+^ and terminally differentiated effector memory re-expressing CD45RA (TEMRA) CD8^+^ cells co-expressing CD27 and CD28; CD8^+^ and TEMRA CD8^+^ cells lacking CD27 and CD28; several T cell subsets expressing CD57.

The algorithm was used to create one-, two-, or three-biomarker panels and compare their ability to classify patients in the correct frailty group. Panels including three biomarkers performed better compared to panels consisting out of only one or two biomarkers. The 10 highest ranked biomarker panels can be found in Table 2 (part b). The highest ranked frailty panels showed an even higher accuracy (≥ 96%) compared to the age panels (90.8–92.3%). Noteworthy, the top ranked biomarker panel for the prediction of frailty status (TIM-3, miR-19b and T-cell *P16^INK4a^)* had an accuracy of 100%, meaning that all the patients were correctly classified as ‘fit’ or ‘frailer’ using this model. L ranged between 12.0 and 24.6% for the top 10 frailty panels. The performance of the different biomarker panels is also demonstrated in the LDA density plots (Figure 1), showing that the frailty groups could be distinguished quite well from each other by the different models.

### 3.3. Performance of the Blood Biomarkers to Discriminate Tumors with Different Immune Infiltration Patterns

We also examined correlations between individual blood biomarkers or biomarker panels and the tumor immune infiltration, as assessed by sTIL level, CD3 and CD8 infiltration in both the whole tumor and the invasive front. The three-biomarker panels showed the highest accuracy and lowest L for categorization of patients according to their levels of tumor infiltration by sTILs, and CD3 and CD8 cells (Table 3). Additionally, we evaluated the importance of age, frailty or BC parameters (tumor size, tumor grade, and lymph node involvement) in relation to the tumor immune infiltrate.

### 3.4. Performance of Individual Blood Biomarkers and BC Parameters for Immune Infiltration Patterns

The individual performance of each of the blood biomarkers, age and BC parameters for categorizing the tumors as ‘high’, ‘intermediate’ or ‘low’ in terms of sTILs, CD3 or CD8 infiltration can be found in Tables S3–S7. When further exploring these tables, we noted that certain biomarkers regularly appeared in the top 10 highest ranked biomarkers. However, these recurrent biomarkers seemed to be somewhat divergent for high infiltration on the one hand *versus* intermediate/low infiltration on the other hand, for all five infiltrate measurements (sTILs; CD3 or CD8 in whole tumor; CD3 or CD8 in invasive front). TEMRA CD8^+^ cell populations as well as IL-1α, TIM-3, Gal-9, and miR-195 were strongly represented among the best performing blood biomarkers for prediction of high sTILs, CD3 and CD8 infiltration, whereas intermediate/low infiltration patterns were rather associated with CM CD8^+^ subsets, total CD4^+^ cells, CD4/CD8 ratio, sCD27, PD-1/PD-L1, and T-cell *P16^INK4a^* expression. As previously reported, patients’ age and tumor grade were also good predictors of a high level of tumor infiltration (sTILs, CD3 and/or CD8 infiltration).

### 3.5. Performance of Three-Biomarker Panels for Immune Infiltration Patterns

**Figure 2:**
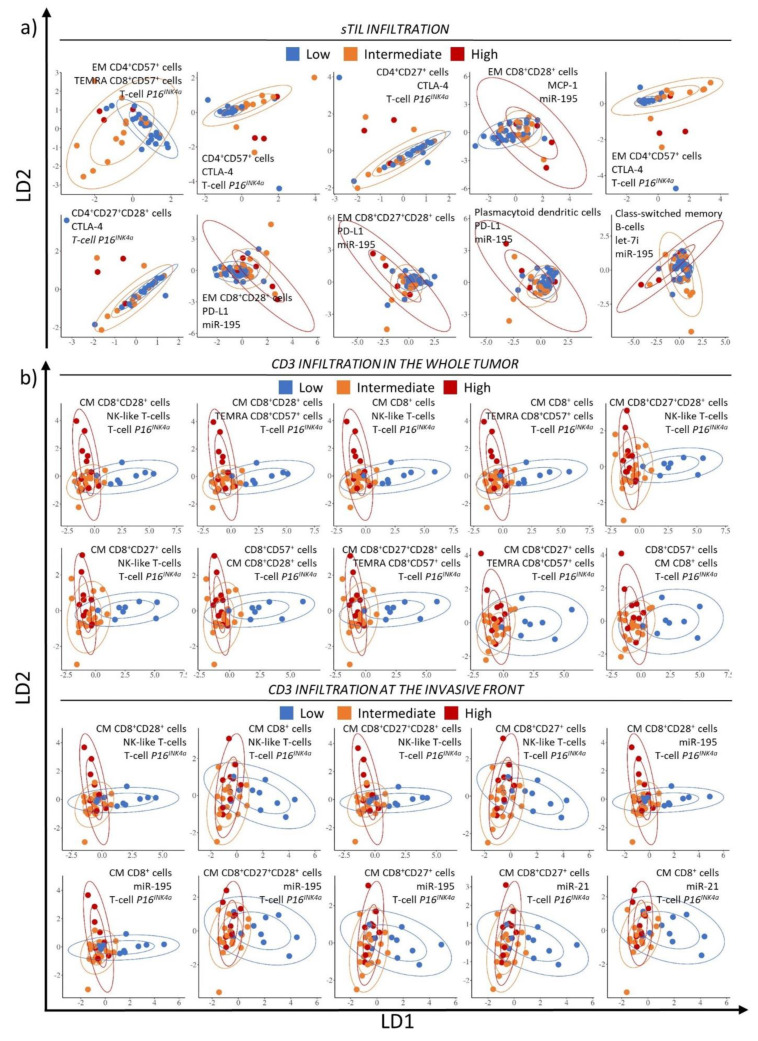

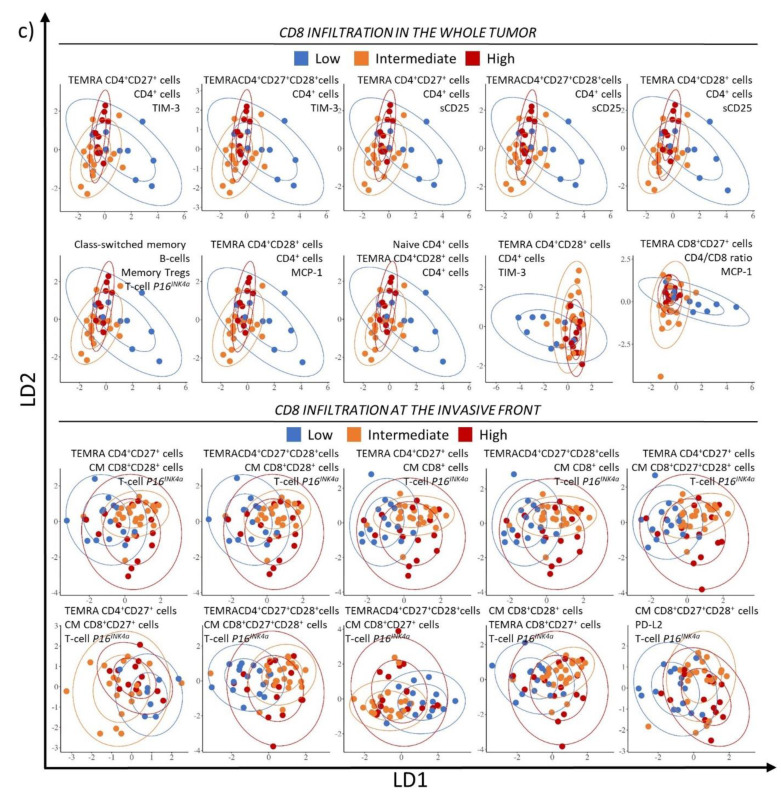
Linear discriminant analysis (LDA) plots of the blood biomarker panels predicting the tumor immune infiltrate in the whole group, with the LD1 coordinate on the x-axis and the LD2 coordinate on the y-axis. Confidence ellipsoids are added as well (66 and 95%). The top 10 highest ranked blood biomarker panels are shown (Table 3). The sTIL (**a**), CD3 (**b**) and CD8 (**c**) infiltration blood biomarker panels are shown. For sTILs a low infiltration corresponded with less than 10% sTILs, an intermediate infiltration with a sTILs percentage between 10 and 40% and a high infiltration with more than 40% sTILs. The cut offs for a low, intermediate or high infiltration were based on IQR of the CD3 and CD8 densities in the whole cohort. A low infiltration reflected tumors with a density in the lower quartile (lowest 25%), while those of high infiltration were the ones with a density in the upper quartile (highest 25%). Tumors with a low infiltration are shown in blue, an intermediate infiltration in orange and a high infiltration in red.

Biomarker combinations were subsequently evaluated for their ability to classify tumors according to their immune infiltration pattern regardless of the age categories. The 10 highest ranked blood three-biomarker panels for sTIL infiltration, CD3 and CD8 infiltration in the whole tumor and invasive front are listed in Table 3 and their LDA score plots are shown in Figure 2a–c. Of note, the top-ranking panels are not necessarily composed of the best performing individual biomarkers identified for the same infiltration variable. The biomarker panels selected for sTIL infiltration had an accuracy of at least 66.7% and L around 40%, the LDA score plots (Figure 2a) show a rather poor separation of the 3 categories (low, intermediate and high sTIL infiltration). The top 10 biomarker panels correlating with CD3 infiltration in the whole tumor had an accuracy of 66.7% and L ranging between 30.2% and 32.7%. In comparison, the biomarker panels correlating with CD3 infiltration in the invasive front had a higher accuracy (up to 76.9%) and slightly lower L (25.6–31.3%). The score LDA plots in Figure 2b show a relatively good separation of low and high CD3 infiltration, although it is hard to distinguish intermediate CD3 infiltrated tumors from either low or highly CD3 infiltrated tumors. Major players in the CD3 infiltration panels were CM CD8^+^ subsets, T-cell *P16^INK4a^*, NK-like T cells, and miR-195 (Table 3). The accuracy of the best biomarker panels for CD8 infiltration in the whole tumor ranged between 66.7 and 70.4% with L between 37.7 and 45.6%. For the invasive front, accuracy of the top 10-biomarker panels ranged between 67.5 and 70.0% with L between 31.3 and 38.6% (Table 3). The score LDA plots for CD8 infiltration (Figure 2c) in the whole tumor show a moderate dissection of the low infiltration category only. Figure 2c also shows a poor separation between the 3 levels of infiltration the invasive front where there is a great deal of overlap.

Next, we examined whether the addition of age or BC clinical parameters (tumor size, grade and lymph node involvement) to the blood biomarker panels could improve the accuracy and/or reduce L. In fact, L indeed tended to decrease with the addition of age, but accuracies were also reduced (Table 3). The reduction of L was even more pronounced when breast tumor characteristics were added instead of age, but the accuracies were not improved, except for the blood biomarker panels correlating with CD3 and CD8 infiltration in the invasive front (Table 3).

## 4. Discussion

In our previous IMAGE study, an extensive number of age and immunosenescence markers were evaluated in the blood of patients with luminal B-type BC. In addition, the tumor immune infiltrate was also investigated in detail, by assessing the amount and spatial distribution of different immune subsets. Numerous age-related as well as frailty-related changes were observed in both blood and tumor [24]. To further elaborate on that study and make optimal use of this unique and comprehensive dataset, we evaluated the ability of (combinations of) blood biomarkers to classify patients into the correct age/frailty group and to distinguish between tumors with different immune infiltration patterns.

In our previous publication [24], we already reported that panels of 5 or 10 immune biomarkers could fairly well separate older patients from younger patients. These panels consisted of combinations of blood and tumor immune biomarkers. Here we show that panels consisting of only three blood biomarkers are also able to achieve quite a clear separation of older patients from younger patients. From a biological point of view, it is intriguing that a person’s chronological age is reflected in such a limited panel of blood biomarkers. However, this finding does not have immediate consequences as the chronological age can be easily deducted from a patients’ medical file.

More importantly, our study also revealed that the blood three-biomarker panels are able to accurately reflect the patients’ fitness status, as assessed by the G8 screening tool (which has been shown to strongly correlate with overall survival in older cancer patients). We have identified several panels with very high accuracies (96–100%) and relatively low L (<20%). Biomarkers constituting these superior frailty panels included the immune checkpoint mediator TIM-3, plasma microRNAs (miR-19b and let-7i), CM CD4^+^ cell subsets, NK-related cell subsets and T cell *p16^INK4a^*. As outlined in the introduction, biological age and frailty status are important factors to take into consideration when treating older patients with cancer [12]. As of now, a patient’s frailty status is estimated via a geriatric screening tool like G8, or via a CGA in clinical practice. This is far from perfect, however, and a CGA is time consuming and requires trained personnel. Even if a CGA can be properly performed, biomarkers could add important biological information to the clinical evaluation of frailty. Moreover, these biomarkers may be able to identify patients at risk of becoming frail. Therefore, an easy and reproducible biomarker panel of clinical frailty would be a major step forward in the field of geriatric oncology, allowing improved personalized treatment based on frailty status. In combination with CGA, such biomarker signatures may yield a more complete picture of the general health status of older patients. Extensive validation studies are of course needed to evaluate whether such frailty biomarker panels are also able to predict clinically relevant outcomes, such as chemotherapy induced toxicity, functional decline, loss of quality of life and independence, and survival.

In the present study, we also evaluated whether individual blood biomarkers or biomarker panels could reflect the level of tumor infiltration (as assessed by sTILs, CD3 and CD8). The results were less clear than the frailty prediction, and the interpretation is more challenging. Even the best ranking blood biomarker combinations afforded only moderate separation between the tumors with different levels of infiltration. Somewhat surprisingly, performance of the biomarker panels could not be improved by adding the BC parameters tumor size, tumor grade, and lymph node involvement to the panels. Noteworthy, the ‘best’ biomarker panels did not necessarily consist out of the highest ranked individual biomarkers. This indicates that specific blood biomarkers can be reinforced when considered in combination with others and modulating interactions between individual biomarkers could be present, making biomarker signatures much more potent than their individual components. Although scientifically interesting, these analyses did not reveal biomarker panels strongly predicting tumor immune infiltration and, thus, did not lead to clinically relevant conclusions.

This study has some limitations. Because of its exploratory nature, cross validation and correction for multiple testing was not performed and further investigation/validation is definitely required. Secondly, as described in our previous publication [24] the study cohort was rather small and the older patients were all relatively fit, with rather small differences in G8 score between fit and frailer patients. Nevertheless, for both age and frailty status we could achieve a good separation between younger and older patients and fitter and frailer patients, respectively. Moreover, an extension of these analyses to healthy subjects might be interesting to gain insights concerning the correlation between blood biomarkers and aging/frailty independent of the tumor. Although the accuracy of biomarker panels correlating with the tumor infiltrate categories was lower compared to that of age and frailty panels, a relatively good separation was observed with some of the blood biomarker panels, especially for the distinction between low and high CD3 infiltration. The prognostic value of TILs and, more specifically, the immune cell subtypes in luminal BC is still not completely understood. Several immune cell subtypes (including CD3^+^ and CD8^+^ cells) have been studied recently, but data in luminal breast tumors is still limited [28]. It may be interesting to further investigate correlations between systemic immunity and the tumor immune infiltrate in a larger patient population. As both age and frailty could have a substantial impact on the immune infiltrate, it would be interesting to stratify the patients in a larger population by age and frailty and evaluate if the blood biomarkers could then be more predictive of the tumor immune infiltrate. However, in this study we wanted to emphasize the utility of blood biomarkers panels for the prediction of frailty level, which is clinically more relevant as it can help with the selection of the appropriate treatment strategy in older patients with cancer. Thirdly, patients included in this study often had an intermediate or low tumor immune infiltration, there were only few tumors with a very high infiltration. This is of course related to the composition of the cohort, which included exclusively luminal B-type tumors, which are usually less heavily infiltrated than triple negative or HER2^+^ tumors [3]. However, the used cut-off for sTILs infiltration applies mostly to triple negative or HER2^+^ tumors, a cut-off for specifically for luminal tumors has not yet been defined. Additionally, only a selection of blood biomarkers was investigated in this study, and evaluating an even broader panel of immune markers in both blood and tumor might result in improved panel performances.

We also want to highlight some strengths of this study. Blood and tumor analyses were conducted on a carefully selected, homogeneous cohort of patients with luminal BC. To date, the immune landscape of this BC subtype remains highly underexplored. In our previous paper, we have demonstrated marked differences between distinct age and frailty groups with regard to both blood and tumor immune biomarker profiles. Now we have further extended these findings by showing that it is possible to identify blood biomarker signatures that reflect the patient’s age and, most importantly, frailty status. Additionally, our data suggest that such signatures, which can easily be measured in the blood, might also give an impression of the tumor immune infiltrate, although the correlation between panels of circulating biomarkers and measurements of tumor immune infiltration was less strong.

## 5. Conclusions

We have identified blood biomarker panels distinguishing frailer patients from fit patients with high accuracy. We also found that panels of multiple biomarkers can release stronger predictive information as compared to individual biomarkers in all categories. This observation suggests that biomarker signatures could be a valuable addition to the standard evaluation of the global health status of older patients. Further investigation is needed to better understand the biological and clinical relevance of our findings, for instance with regard to prediction to (immune) therapies in older BC patients.

## Figures and Tables

**Table 1:** Patient, tumor, and tumor infiltrate characteristics. Definitions: sTILs infiltration (‘low’: <10%, ‘intermediate’: 10–40%, ‘high’: >40%), CD3 and CD8 infiltration (cutoffs based on IQR of CD3 and CD8 density: ‘low’: lowest 25%, ‘high’: highest 25%). The inclusion criteria were based on clinical estimate of the tumor size (1.5 cm or bigger) and on the grading (grade II or III) based on the core needle biopsy. Enough tumor material was available for 62 out of the 65 patients. The table reports pathological tumor size and tumor grade measured on the resection specimen after surgery, explaining a few discrepancies between selection criteria and results on the surgical specimen reported here. For two patients in the old group the G8 scores were not available.

Variable	Statistic	All	35–45/55–65 Years	≥70 Years
Age				
	*N* (%)	65	34	31
	Mean	63.4	51.6	76.3
	(Range)	(35.0; 89.0)	(35.0; 65.0)	(70.0; 89.0)
G8 score				
All	*N*			29
	Mean			15.2
	Range			(12.0; 17.0)
Fitter older patients (G8 > 14)	*N*			19
	Mean			16.1
	Range			(15.0; 17.0)
Frailer older patients (G8 ≤ 14)	*N*			10
	Mean			13.7
	Range			(12.0; 14.0)
Tumor histological Subtype				
Ductal (IDA)	*n/N* (%)	54/65 (83.1)	15/34 (44.1)	26/31 (83.9)
Lobular (ILA)	*n/N* (%)	5/65 (7.7)	3/34 (8.8)	2/31 (6.5)
Mixed ILA-IDA	*n/N* (%)	2/65 (3.1)	1/34 (2.9)	1/31 (3.2)
Invasive solid papillary	*n/N* (%)	2/65 (3.1)	1/34 (2.9)	1/31 (3.2)
Micro-papillary	*n/N* (%)	1/65 (1.5)	0/34 (0.0)	1/31 (3.2)
Mixed micro-papillary and mucinous	*n/N* (%)	1/65 (1.5)	1/34 (2.9)	0/31 (0.0)
Tumor Grade				
Grade I	*n/N* (%)	1/65 (0.02)	0/34 (0.0)	1/31 (0.03)
Grade II	*n/N* (%)	40/65 (61.5)	19/34 (55.9)	21/31 (67.7)
Grade III	*n/N* (%)	24/65 (36.9)	15/34 (44.1)	9/31 (29.0)
Tumor Size (mm)				
	*N*	65	34	31
	Mean	31.8	29.8	34.0
	Range	(10.0; 115.0)	(10.0; 60.0)	(12.0; 115.0)
Node status				
pN0	*n/N* (%)	32/65 (49.2)	15/34 (44.1)	17/31 (54.8)
pN1	*n/N* (%)	29/65 (44.6)	16/34 (47.1)	12/31 (38.7)
pN2	*n/N* (%)	3/65 (4.6)	2/34 (5.9)	1/31 (3.2)
pN3	*n/N* (%)	1/65 (1.5)	0/34 (0.0)	1/31 (3.2)
sTIL infiltration				
Low	*n/N* (%)	37/62 (59.7)	17/33 (51.5)	20/29 (69.0)
Intermediate	*n/N* (%)	20/62 (32.3)	12/33 (36.4)	8/29 (27.6)
High	*n/N* (%)	5/62 (8.1)	4/33 (12.1)	1/29 (3.4)
CD3 infiltration whole tumor				
Low	*n/N* (%)	15/61 (24.6)	6/33 (18.2)	9/28 (32.1)
Intermediate	*n/N* (%)	31/61 (50.8)	14/33 (42.4)	17/28 (60.7)
High	*n/N* (%)	15/61 (24.6)	13/33 (39.4)	2/28 (7.1)
CD3 infiltration invasive front				
Low	*n/N* (%)	15/61 (24.6)	6/33 (18.2)	9/28 (32.1)
Intermediate	*n/N* (%)	31/61 (50.8)	15/33 (45.5)	16/28 (57.1)
High	*n/N* (%)	15/61 (24.6)	12/33 (36.4)	3/28 (10.7)
CD8 infiltration whole tumor				
Low	*n/N* (%)	16/62 (25.8)	6/33 (18.2)	10/29 (34.5)
Intermediate	*n/N* (%)	30/62 (48.4)	14/33 (42.4)	16/29 (55.2)
High	*n/N* (%)	15/62 (24.2)	13/33 (39.4)	3/29 (10.3)
CD8 infiltration invasive front				
Low	*n/N* (%)	16/62 (25.8)	6/33 (18.2)	10/29 (34.5)
Intermediate	*n/N* (%)	30/62 (48.4)	14/33 (42.4)	16/29 (55.2)
High	*n/N* (%)	15/62 (24.2)	13/33 (39.4)	3/29 (10.3)

**Table 2:** Based on their performance (i.e., accuracy and loss), the biomarker panels were assigned a rank. The 10 highest ranked (i.e., highest accuracy and lowest loss) biomarker panels to classify patients in (a) the age groups: younger age group (35–45/55–65 years) or the older age group (≥70 years), and (b) the frailty groups: fitter group of older patients (G8 > 14) or the frailer group of older patients (G8 ≤ 14) are shown. The number of patients (*N*) for whom a specific panel could be assessed, as well as the panels’ accuracies and losses are reported.

Rank	Biomarker Panel			*N*	Accuracy (%)	Loss (%)
**(a)**	**Age**					
1	Gal-9	miR-19b	miR-326	65	92.3	13.7
2	miR-19b	miR-326	miR-424	65	92.3	17.4
3	CD8^+^ cells	TIM-3	miR-326	57	91.2	14.4
4	CM CD4^+^CD57^+^ cells	miR-18a	miR-326	57	91.2	15.4
5	CD4^+^ cells	TIM-3	miR-326	57	91.2	15.5
6	IP-10	miR-195	miR-326	65	90.8	12.8
7	IL-1α	miR-18a	miR-326	65	90.8	13.7
8	miR-18a	miR-195	miR-326	65	90.8	13.9
9	TIM-3	miR-195	miR-326	65	90.8	13.9
10	IL-1α	let-7i	miR-326	65	90.8	14.0
**(b)**	**G8**					
1	TIM-3	miR-19b	T-cell *P16^INK4a^*	20	100.0	12.0
2	CD56^bright^CD16^−^ NK-cells	NK-like T-cells	let-7i	25	96.0	14.8
3	CM CD4^+^CD27^+^CD28^+^ cells	NK-like T-cells	let-7i	25	96.0	18.8
4	CM CD4^+^CD27^+^ cells	NK-like T-cells	let-7i	25	96.0	18.8
5	CM CD4^+^CD28^+^ cells	NK-like T-cells	let-7i	25	96.0	19.0
6	CD4^+^ Tregs	NK-like T-cells	let-7i	25	96.0	19.0
7	CM CD4^+^ cells	NK-like T-cells	let-7i	25	96.0	19.1
8	Hematopoietic stem cells	NK-like T-cells	let-7i	25	96.0	20.3
9	Hematopoietic stem cells	NK-like T-cells	miR-146a	25	96.0	21.2
10	CD4^+^ Tregs	NK-like T-cells	MCP-1	25	96.0	24.6

**Table 3:** Based on the performance of the biomarker panels i.e., highest accuracy and lowest loss, the biomarker panels were assigned a rank. The 10 highest ranked biomarker panels correlating with the tumor immune infiltrate for all patients are shown: (a) sTILs infiltration, (b) CD3 infiltration in the whole tumor, (c) CD3 infiltration at the invasive front, (d) CD8 infiltration in the whole tumor, and (e) CD8 infiltration at the invasive front. The number of patients (*N*) for whom a specific panel could be assessed, the panels’ accuracies and losses are reported as well as the accuracy and loss of the panel when age or BC parameters (tumor size, tumor grade and lymph node involvement) are added to it.

Rank	Biomarker Panel			*N*	Accuracy (%)	Loss (%)	Accuracy with Age (%)	Loss with Age (%)	Accuracy with BC Parameters (%)	Loss with BC Parameters (%)
(a)	sTIL INFILTRATION									
1	EM CD4^+^CD57^+^ cells	TEMRA CD8^+^CD57^+^ cells	T-cell *P16^INK4a^*	40	70.0	34.1	60.0	30.8	52.5	27.5
2	CD4^+^CD57^+^ cells	CTLA-4	T-cell *P16^INK4a^*	40	70.0	38.9	65.0	28.3	65.0	25.3
3	CD4^+^CD27^+^ cells	CTLA-4	T-cell *P16^INK4a^*	40	70.0	43.8	55.0	37.1	50.0	28.8
4	EM CD8^+^CD28^+^ cells	MCP-1	miR-195	54	68.5	40.0	63.0	38.0	59.3	32.4
5	EM CD4^+^CD57^+^ cells	CTLA-4	T-cell *P16^INK4a^*	40	67.5	37.6	67.5	34.4	60.0	26.6
6	CD4^+^CD27^+^CD28^+^ cells	CTLA-4	T-cell *P16^INK4a^*	40	67.5	44.6	52.5	37.9	47.5	29.3
7	EM CD8^+^CD28^+^ cells	PD-L1	miR-195	54	66.7	42.6	55.6	39.7	61.1	33.0
8	EM CD8^+^CD27^+^CD28^+^ cells	PD-L1	miR-195	54	66.7	43.3	57.4	39.2	57.4	33.3
9	Plasmacytoid dendritic cells	PD-L1	miR-195	54	66.7	44.4	59.3	41.7	55.6	33.5
10	Class-switched memory B-cells	let-7i	miR-195	54	66.7	44.7	61.1	39.0	57.4	35.8
(b)	CD3 INFILTRATION WHOLE TUMOR									
1	CM CD8^+^CD28^+^ cells	NK-like T-cells	T-cell *P16^INK4a^*	39	66.7	30.2	59.0	25.1	64.1	16.7
2	CM CD8^+^CD28^+^ cells	TEMRA CD8^+^CD57^+^ cells	T-cell *P16^INK4a^*	39	66.7	30.4	59.0	24.5	66.7	16.0
3	CM CD8^+^ cells	NK-like T-cells	T-cell *P16^INK4a^*	39	66.7	31.0	59.0	25.9	64.1	18.1
4	CM CD8^+^ cells	TEMRA CD8^+^CD57^+^ cells	T-cell *P16^INK4a^*	39	66.7	31.1	59.0	25.2	66.7	17.3
5	CM CD8^+^CD27^+^CD28^+^ cells	NK-like T-cells	T-cell *P16^INK4a^*	39	66.7	31.8	59.0	26.4	64.1	17.5
6	CM CD8^+^CD27^+^ cells	NK-like T-cells	T-cell *P16^INK4a^*	39	66.7	32.0	59.0	26.5	64.1	17.8
7	CD8^+^CD57^+^ cells	CM CD8^+^CD28^+^ cells	T-cell *P16^INK4a^*	39	66.7	32.1	59.0	25.3	66.7	17.2
8	CM CD8^+^CD27^+^CD28^+^ cells	TEMRA CD8^+^CD57^+^ cells	T-cell *P16^INK4a^*	39	66.7	32.1	59.0	25.5	66.7	16.7
9	CM CD8^+^CD27^+^ cells	TEMRA CD8^+^CD57^+^ cells	T-cell *P16^INK4a^*	39	66.7	32.2	59.0	25.5	66.7	17.0
10	CD8^+^CD57^+^ cells	CM CD8^+^ cells	T-cell *P16^INK4a^*	39	66.7	32.7	59.0	26.2	64.1	17.9
(c)	CD3 INFILTRATION INVASIVE FRONT									
1	CM CD8^+^CD28^+^ cells	NK-like T-cells	T-cell *P16^INK4a^*	39	76.9	25.6	74.4	23.0	79.5	11.8
2	CM CD8^+^ cells	NK-like T-cells	T-cell *P16^INK4a^*	39	76.9	26.2	74.4	23.7	79.5	12.1
3	CM CD8^+^CD27^+^CD28^+^ cells	NK-like T-cells	T-cell *P16^INK4a^*	39	76.9	26.3	74.4	23.5	82.1	12.9
4	CM CD8^+^CD27^+^ cells	NK-like T-cells	T-cell *P16^INK4a^*	39	76.9	26.3	74.4	23.8	79.5	13.2
5	CM CD8^+^CD28^+^ cells	miR-195	T-cell *P16^INK4a^*	39	71.8	28.8	64.1	22.5	82.1	11.2
6	CM CD8^+^ cells	miR-195	T-cell *P16^INK4a^*	39	71.8	29.3	64.1	22.9	79.5	12.2
7	CM CD8^+^CD27^+^CD28^+^ cells	miR-195	T-cell *P16^INK4a^*	39	71.8	29.4	61.5	22.9	79.5	12.0
8	CM CD8^+^CD27^+^ cells	miR-195	T-cell *P16^INK4a^*	39	71.8	29.7	61.5	23.1	79.5	12.4
9	CM CD8^+^CD27^+^ cells	miR-21	T-cell *P16^INK4a^*	39	71.8	32.0	64.1	29.2	76.9	13.5
10	CM CD8^+^ cells	miR-21	T-cell *P16^INK4a^*	39	69.2	31.3	64.1	28.9	76.9	12.5
(d)	CD8 INFILTRATION WHOLE TUMOR									
1	TEMRA CD4^+^CD27^+^ cells	CD4^+^ cells	TIM-3	54	70.4	37.4	59.3	34.2	63.0	27.5
2	TEMRA CD4^+^CD27^+^CD28^+^ cells	CD4^+^ cells	TIM-3	54	70.4	37.6	59.3	34.5	63.0	27.4
3	TEMRA CD4^+^CD27^+^ cells	CD4^+^ cells	sCD25	54	70.4	38.9	59.3	35.8	57.4	27.5
4	TEMRA CD4^+^CD27^+^CD28^+^ cells	CD4^+^ cells	sCD25	54	70.4	38.9	57.4	36.0	57.4	27.5
5	TEMRA CD4^+^CD28^+^ cells	CD4^+^ cells	sCD25	54	68.5	38.6	57.4	33.9	59.3	27.3
6	Class-switched memory B-cells	Memory Tregs	T-cell *P16^INK4a^*	40	67.5	45.6	60.0	37.2	72.5	31.5
7	TEMRA CD4^+^CD28^+^ cells	CD4^+^ cells	MCP-1	54	66.7	37.0	61.1	31.9	61.1	25.4
8	Naive CD4^+^ cells	TEMRA CD4^+^CD28^+^ cells	CD4^+^ cells	54	66.7	37.1	57.4	30.4	55.6	25.2
9	TEMRA CD4^+^CD28^+^ cells	CD4^+^ cells	TIM-3	54	66.7	37.9	57.4	33.1	63.0	27.3
10	TEMRA CD8^+^CD27^+^ cells	CD4/CD8 ratio	MCP-1	54	66.7	40.2	59.3	38.3	57.4	29.2
(e)	CD8 INFILTRATION INVASIVE FRONT									
1	TEMRA CD4^+^CD27^+^ cells	CM CD8^+^CD28^+^ cells	T-cell *P16^INK4a^*	40	70.0	31.3	65.0	21.7	75.0	18.7
2	TEMRA CD4^+^CD27^+^CD28^+^ cells	CM CD8^+^CD28^+^ cells	T-cell *P16^INK4a^*	40	70.0	31.6	65.0	21.9	75.0	18.8
3	TEMRA CD4^+^CD27^+^ cells	CM CD8^+^ cells	T-cell *P16^INK4a^*	40	70.0	31.8	65.0	22.3	75.0	19.2
4	TEMRA CD4^+^CD27^+^CD28^+^ cells	CM CD8^+^ cells	T-cell *P16^INK4a^*	40	70.0	32.0	65.0	22.4	75.0	19.3
5	TEMRA CD4^+^CD27^+^ cells	CM CD8^+^CD27^+^CD28^+^ cells	T-cell *P16^INK4a^*	40	70.0	32.1	65.0	22.3	75.0	19.2
6	TEMRA CD4^+^CD27^+^ cells	CM CD8^+^CD27^+^ cells	T-cell *P16^INK4a^*	40	70.0	32.1	65.0	22.3	75.0	19.3
7	TEMRA CD4^+^CD27^+^CD28^+^ cells	CM CD8^+^CD27^+^CD28^+^ cells	T-cell *P16^INK4a^*	40	70.0	32.3	65.0	22.5	75.0	19.4
8	TEMRA CD4^+^CD27^+^CD28^+^ cells	CM CD8^+^CD27^+^ cells	T-cell *P16^INK4a^*	40	67.5	32.4	65.0	22.5	75.0	19.5
9	CM CD8^+^CD28^+^ cells	TEMRA CD8^+^CD27^+^ cells	T-cell *P16^INK4a^*	40	67.5	33.0	57.5	26.5	65.0	19.0
10	CM CD8^+^CD27^+^CD28^+^ cells	PD-L2	T-cell *P16^INK4a^*	40	67.5	38.6	62.5	25.2	72.5	22.5

## Data Availability

The data presented in this study are available in the supplementary tables and in our previous publication [24].

## Supplementary Materials

The following are available online at https://www.mdpi.com/article/10.3390/cancers13092185/s1, Table S1: Individual performance_age, Table S2: Individual performance_frailty, Table S3: Individual performance_TILs, Table S4: Individual performance_CD3 infiltration whole tumor, Table S5: Individual performance_CD3 infiltration invasive front, Table S6: Individual performance_CD8 infiltration whole tumor, Table S7: Individual performance_CD8 infiltration invasive front.
