# Supplementary material for: Blood Immunosenescence Signatures Reflecting Age, Frailty and Tumor Immune Infiltrate in Patients with Early Luminal Breast Cancer
Source: Cancers (Basel). 2021 May 2;13(9):2185. doi: 10.3390/cancers13092185 (PMC8125302; doi:10.3390/cancers13092185)
Supplement: Supplementary file 1 [file cancers-13-02185-s001.zip › Table S1 - Individual performance_age.pdf]

Table S1: Individual performances of biomarkers for the classification of patients in the two different age groups: young: 35-45/55-65 years and old: ≥70 years. The table reports the number of patients (N) for which the biomarkers could be measured, as well as the area under the curve (AUC) via receiver operating characteristics (ROC), P-value (Wilcox rank-sum test) and log fold change (FC) for each biomarker. The log FC compared case vs. control. A positive log FC indicates that the measurement is higher than its reference while a negative measurement indicates that is smaller. For each of these statistics ranking scores were computed reflecting the ability of the statistic to classify the patient in the correct age group. The final score combines the 3 scores, where AUC weighted double. The biomarkers are ranked based on their final score.

	Blood markers	N	AUC	*P*-value	log FC	AUC score	*P*-value score	log FC score	Final score
1	miR-326	65	0.887	< 0.001		1	1	1	1.00
2	miR-155	65	0.765	< 0.001	-1.011	5	6	8	6.00
3	miR-18a	65	0.218	< 0.001	0.633	3	3	18	6.75
4	T-cell *P16^INK4a^*	43	0.760	0.004	-1.870	7	9	6	7.25
5	IL-1α	65	0.797	< 0.001	-0.522	2	2	24	7.50
6	miR-19b	65	0.222	< 0.001	0.617	4	4	20	8.00
7	Gal-9	65	0.762	< 0.001	-0.614	6	5	21	9.50
8	Naive $CD8^+CD27^+$ cells	57	0.291	0.007	0.850	10	10	11	10.25
9	Naive $CD8^+CD27^+CD28^+$ cells	57	0.297	0.008	0.840	11	11	12	11.25
10	IL-17A	65	0.313	0.010	2.194	15	12	5	11.75
11	IP-10	65	0.758	< 0.001	-0.427	8	7	29	13.00
12	Naive $CD8^+$ cells	57	0.308	0.013	0.742	13	14	15	13.75
13	Naive $CD8^+CD28^+$ cells	57	0.308	0.013	0.784	14	15	14	14.25
14	IGF-1	65	0.286	0.003	0.372	9	8	35	15.25
15	$CD8^+CD27^+$ cells	57	0.300	0.010	0.461	12	13	25	15.50
16	miR-195	65	0.333	0.021	0.638	17	18	17	17.25
17	miR-9	65	0.356	0.022	1.363	23	19	7	18.00
18	4-1BB	65	0.361	0.019	2.417	28	16	4	19.00
19	$CD8^+CD27^+CD28^+$ cells	57	0.319	0.020	0.444	16	17	27	19.00
20	$CD56^{bright}CD16^-$ NK-cells	57	0.340	0.040	0.595	18	21	22	19.75
21	miR-19a	65	0.357	0.049	0.389	24	23	32	25.75
22	miR-20a	65	0.342	0.029	0.283	19	20	50	27.00
23	miR-125b	65	0.358	0.050	0.348	25	24	36	27.50
24	Intermediate monocytes	57	0.646	0.059	-0.284	22	27	49	30.00
25	EM $CD8^+CD27^+$ cells	57	0.352	0.058	0.278	21	26	53	30.25
26	$CD8^+CD27^-CD28^-$ cells	57	0.622	0.119	-0.388	31	32	33	31.75
27	TEMRA $CD8^+CD27^-CD28^-$ cells	57	0.620	0.124	-0.426	33	33	30	32.25
28	let-7e	65	0.628	0.077	-0.302	30	29	45	33.50
29	miR-21	65	0.379	0.095	0.329	32	31	40	33.75
30	Plasmacytoid dendritic cells	57	0.364	0.079	0.249	29	30	61	37.25
31	EM $CD8^+CD27^+CD28^+$ cells	57	0.360	0.071	0.197	26	28	74	38.50
32	TEMRA $CD8^+$ cells	57	0.604	0.181	-0.322	37	39	42	38.75
33	$CD56^{dim}CD16^+$ NK-cells	57	0.654	0.047	-0.063	20	22	102	41.00
34	TEMRA $CD4^+$ cells	57	0.592	0.241	-0.332	43	46	37	42.25
35	TEMRA $CD8^+CD27^+$ cells	57	0.398	0.192	0.279	39	40	51	42.25
36	miR-126	65	0.406	0.193	0.285	41	42	48	43.00
37	MCP-1	65	0.611	0.128	-0.181	34	34	77	44.75
38	PD-L2	65	0.397	0.155	0.194	38	35	75	46.50

39	IL-8	65	0.639	0.055	-0.051	27	25	110	47.25
40	Naive CD4$^+$CD57$^+$ cells	57	0.567	0.391	-0.440	54	56	28	48.00
41	EM CD8$^+$CD27$^-$CD28$^-$ cells	57	0.582	0.294	-0.295	48	50	47	48.25
42	NK-cells	57	0.588	0.261	-0.278	46	49	52	48.25
43	CD4$^+$CD27$^+$CD28$^+$ cells	57	0.392	0.167	0.121	35	36	87	48.25
44	PD-1	65	0.432	0.351	-0.320	52	54	43	50.25
45	miR-181a	65	0.432	0.351	0.326	53	55	41	50.50
46	CRP	65	0.584	0.247	-0.250	47	48	60	50.50
47	TEMRA CD8$^+$CD57$^+$ cells	57	0.582	0.296	-0.273	49	51	55	51.00
48	CD8$^+$CD28$^+$ cells	57	0.406	0.228	0.193	42	45	76	51.25
49	PD-L1	65	0.444	0.442	0.831	64	65	13	51.50
50	sCD25	65	0.588	0.226	-0.198	45	44	72	51.50
51	Hematopoietic stem cells	57	0.421	0.307	0.276	51	52	54	52.00
52	TIM-3	65	0.562	0.399	-0.416	61	58	31	52.75
53	IL-17F	65	0.457	0.206	3.910	83	43	3	53.00
54	B-cells	57	0.435	0.412	0.318	56	60	44	54.00
55	Non-switched memory B-cells	57	0.395	0.177	0.046	36	37	113	55.50
56	TEMRA CD8$^+$CD27$^+$CD28$^+$ cells	57	0.434	0.400	0.272	55	59	56	56.25
57	CD4$^+$CD27$^+$ cells	57	0.409	0.242	0.099	44	47	93	57.00
58	Classical monocytes	57	0.398	0.193	0.052	40	41	108	57.25
59	Free active TGF-β1	65	0.471	0.181		95	38	2	57.50
60	TEMRA CD4$^+$CD27$^-$CD28$^-$ cells	57	0.551	0.516	-0.630	70	73	19	58.00
61	sCD27	65	0.563	0.392	-0.242	60	57	63	60.00
62	Naive CD4$^+$CD27$^-$CD28$^-$ cells	57	0.545	0.564	-0.868	76	79	10	60.25
63	CD4$^+$CD57$^+$ cells	57	0.552	0.509	-0.332	67	70	38	60.50
64	EM CD4$^+$CD57$^+$ cells	57	0.579	0.309	0.103	50	53	91	61.00
65	Non-classical monocytes	57	0.563	0.421	-0.208	59	63	68	62.25
66	miR-150	65	0.444	0.446	0.272	65	66	57	63.25
67	CD8$^+$CD57$^+$ cells	57	0.563	0.421	-0.179	58	62	78	64.00
68	Naive CD4$^+$CD27$^+$CD28$^+$ cells	57	0.437	0.421	0.141	57	61	83	64.50
69	CM CD4$^+$CD57$^+$ cells	57	0.547	0.553	-0.331	75	78	39	66.75
70	CD4$^+$CD27$^-$CD28$^-$ cells	57	0.543	0.586	-0.388	81	83	34	69.75
71	NK-like T-cells	57	0.553	0.501	-0.156	66	69	81	70.50
72	CM CD8$^+$CD27$^-$CD28$^-$ cells	57	0.535	0.654	-0.542	88	90	23	72.25
73	EM CD8$^+$CD57$^+$ cells	57	0.558	0.459	-0.081	63	67	99	73.00
74	EM CD8$^+$CD28$^+$ cells	57	0.439	0.440	0.059	62	64	104	73.00
75	EM CD4$^+$CD27$^+$ cells	57	0.453	0.551	0.198	73	75	73	73.50
76	EM CD4$^+$CD27$^+$CD28$^+$ cells	57	0.453	0.551	0.199	74	76	71	73.75
77	TEMRA CD4$^+$CD57$^+$ cells	57	0.531	0.695	-0.706	94	95	16	74.75
78	Naive CD8$^+$CD57$^+$ cells	57	0.456	0.575	0.254	79	82	59	74.75
79	IL-6	65	0.550	0.495	-0.108	71	68	89	74.75
80	Naive CD4$^+$CD27$^+$ cells	57	0.448	0.509	0.102	68	71	92	74.75
81	CD86	65	0.456	0.553	0.213	80	77	67	76.00
82	CTLA-4	65	0.480	0.668	-0.458	103	93	26	81.25
83	EM CD4$^+$CD27$^-$CD28$^-$ cells	57	0.551	0.511	-0.030	69	72	118	82.00

84	Naive $CD8^+CD27^-CD28^-$ cells	57	0.450	0.527	-0.045	72	74	115	83.25
85	IL12p70	65	0.535	0.631	-0.216	90	88	66	83.50
86	TNF-α	65	0.539	0.594	-0.177	85	85	79	83.50
87	Naive $CD4^+$ cells	57	0.455	0.572	0.078	77	80	101	83.75
88	CM $CD8^+CD27^+$ cells	57	0.468	0.689	0.257	92	94	58	84.00
89	miR-424	65	0.537	0.613	-0.148	86	86	82	85.00
90	Tregs	57	0.527	0.730	-0.298	99	101	46	86.25
91	Naive $CD4^+CD28^+$ cells	57	0.457	0.586	0.081	82	84	97	86.25
92	Myeloid dendritic cells	57	0.461	0.619	0.080	84	87	100	88.75
93	CM $CD8^+CD27^+CD28^+$ cells	57	0.471	0.721	0.242	98	100	62	89.50
94	$CD8^+$ cells	57	0.455	0.572	0.019	78	81	121	89.50
95	CM $CD4^+CD27^+CD28^+$ cells	57	0.463	0.642	0.050	87	89	111	93.50
96	CM $CD4^+CD27^-CD28^-$ cells	57	0.504	0.960	-0.967	123	124	9	94.75
97	Memory Tregs	57	0.465	0.662	0.055	91	92	105	94.75
98	CM $CD8^+CD57^+$ cells	57	0.482	0.822	0.236	105	106	64	95.00
99	EM $CD4^+CD28^+$ cells	57	0.471	0.721	0.118	97	99	88	95.25
100	CM $CD4^+CD27^+$ cells	57	0.465	0.654	0.048	89	91	112	95.25
101	miR-17	65	0.473	0.713	0.134	100	97	85	95.50
102	$CD4^+CD28^+$ cells	57	0.469	0.695	0.051	93	96	109	97.75
103	TEMRA $CD4^+CD28^+$ cells	57	0.516	0.841	-0.203	108	108	70	98.50
104	$CD4^+$ Tregs	57	0.529	0.718	-0.054	96	98	106	99.00
105	miR-223	65	0.517	0.818	-0.081	106	105	98	103.75
106	IL-1β	65	0.517	0.823	-0.087	107	107	96	104.25
107	TEMRA $CD4^+CD27^+CD28^+$ cells	57	0.475	0.755	0.021	101	102	120	106.00
108	miR-146a	65	0.511	0.880	0.127	114	114	86	107.00
109	miR-92a	65	0.522	0.763	0.010	102	103	125	108.00
110	CM $CD4^+$ cells	57	0.485	0.855	-0.052	109	110	107	108.75
111	EM $CD8^+$ cells	57	0.520	0.805	0.009	104	104	126	109.50
112	IL-10	65	0.504	0.958	-0.205	124	123	69	110.00
113	LAG-3	65	0.493	0.932	0.164	120	120	80	110.00
114	CD4/CD8 Ratio	57	0.504	0.968	-0.236	126	125	65	110.50
115	CM $CD4^+CD28^+$ cells	57	0.485	0.855	-0.046	110	111	114	111.25
116	IFN-γ	65	0.514	0.854	-0.041	112	109	116	112.25
117	CM $CD8^+$ cells	57	0.509	0.911	0.099	119	119	94	112.75
118	CM $CD8^+CD28^+$ cells	57	0.505	0.955	0.106	122	121	90	113.75
119	EM $CD4^+$ cells	57	0.505	0.955	0.094	121	122	95	114.75
120	IL-27	65	0.511	0.880	-0.016	113	113	122	115.25
121	$CD4^+$ cells	57	0.485	0.855	0.000	111	112	129	115.75
122	let-7i	65	0.511	0.885	-0.040	116	115	117	116.00
123	Monocytes	57	0.500	1.000	-0.136	128	128	84	117.00
124	Naive B-cells	57	0.490	0.905	0.026	118	118	119	118.25
125	TEMRA $CD4^+CD27^+$ cells	57	0.489	0.898	-0.015	117	117	123	118.50
126	$CD3^+$ cells	57	0.511	0.892	0.001	115	116	128	118.50
127	TEMRA $CD8^+CD28^+$ cells	57	0.496	0.968	-0.062	125	126	103	119.75
128	Class-switched memory B-cells	57	0.501	1.000	0.014	127	127	124	126.25

129	Naive Tregs	57	0.500	1.000	0.006	129	129	127	128.50
