# Supplementary material for: Blood Immunosenescence Signatures Reflecting Age, Frailty and Tumor Immune Infiltrate in Patients with Early Luminal Breast Cancer
Source: Cancers (Basel). 2021 May 2;13(9):2185. doi: 10.3390/cancers13092185 (PMC8125302; doi:10.3390/cancers13092185)
Supplement: Supplementary file 1 [file cancers-13-02185-s001.zip › Table S2 - Individual performance_frailty.pdf]

*Table S2: Individual performances of biomarkers classification of frailty groups (fitter older patients: G8>14 and frailer older patients: G8≤14). The table reports the number of patients (N) for which the biomarkers could be measured, as well as the area under the curve (AUC) via receiver operating characteristics (ROC), P-value (Wilcox rank-sum test) and log fold change (FC) for each biomarker. The log FC compared case vs. control. A positive log FC indicates that the measurement is higher than its reference while a negative measurement indicates that is smaller. Based on these statistics AUC, P-value, log FC scores were computed. The final score combines the 3 scores, where AUC weighted double. The biomarkers are ranked based on their final score.*

| | Blood markers | N | AUC | *P*-value | log FC | AUC score | *P*-value score | log FC score | Final score |
|---|---|---|---|---|---|---|---|---|---|
| 1 | NK-like T-cells | 25 | 0.941 | < 0.001 | -1.623 | 1 | 1 | 6 | 2.25 |
| 2 | TEMRA $CD8^+CD57^+$ cells | 25 | 0.860 | 0.003 | -0.980 | 2 | 2 | 14 | 5.00 |
| 3 | T-cell *$P16^{INK4a}$* | 20 | 0.798 | 0.041 | -1.937 | 7 | 8 | 4 | 6.50 |
| 4 | TEMRA $CD8^+CD27^-CD28^-$ cells | 25 | 0.816 | 0.011 | -0.897 | 5 | 5 | 17 | 8.00 |
| 5 | $CD8^+CD27^-CD28^-$ cells | 25 | 0.816 | 0.011 | -0.754 | 4 | 4 | 30 | 10.50 |
| 6 | $CD8^+CD57^+$ cells | 25 | 0.846 | 0.005 | -0.671 | 3 | 3 | 36 | 11.25 |
| 7 | EM $CD4^+CD57^+$ cells | 25 | 0.743 | 0.057 | -1.070 | 11 | 13 | 13 | 12.00 |
| 8 | TEMRA $CD8^+CD27^+CD28^+$ cells | 25 | 0.243 | 0.044 | 0.771 | 10 | 9 | 26 | 13.75 |
| 9 | TEMRA $CD4^+CD57^+$ cells | 25 | 0.728 | 0.076 | -1.198 | 14 | 19 | 9 | 14.00 |
| 10 | $CD8^+CD27^+CD28^+$ cells | 25 | 0.221 | 0.027 | 0.710 | 8 | 7 | 33 | 14.00 |
| 11 | $CD8^+CD28^+$ cells | 25 | 0.191 | 0.013 | 0.629 | 6 | 6 | 43 | 15.25 |
| 12 | $CD4^+CD27^-CD28^-$ cells | 25 | 0.721 | 0.086 | -1.171 | 16 | 20 | 10 | 15.50 |
| 13 | $CD8^+CD27^+$ cells | 25 | 0.243 | 0.044 | 0.652 | 9 | 10 | 38 | 16.50 |
| 14 | CM $CD8^+$ cells | 25 | 0.279 | 0.086 | 0.902 | 17 | 21 | 16 | 17.75 |
| 15 | CM $CD8^+CD27^+$ cells | 25 | 0.287 | 0.097 | 0.910 | 20 | 24 | 15 | 19.75 |
| 16 | Gal-9 | 29 | 0.716 | 0.062 | -0.656 | 18 | 15 | 37 | 22.00 |
| 17 | $CD4^+CD57^+$ cells | 25 | 0.706 | 0.110 | -1.077 | 25 | 28 | 12 | 22.50 |
| 18 | TEMRA $CD8^+CD27^+$ cells | 25 | 0.257 | 0.057 | 0.512 | 12 | 14 | 53 | 22.75 |
| 19 | Naive $CD8^+CD27^-CD28^-$ cells | 25 | 0.713 | 0.097 | -0.756 | 21 | 22 | 28 | 23.00 |
| 20 | $CD4^+$ Tregs | 25 | 0.287 | 0.097 | 0.735 | 19 | 23 | 32 | 23.25 |
| 21 | CM $CD8^+CD28^+$ cells | 25 | 0.294 | 0.109 | 0.887 | 27 | 27 | 18 | 24.75 |
| 22 | miR-9 | 29 | 0.342 | 0.056 | | 45 | 12 | 1 | 25.75 |
| 23 | EM $CD4^+CD27^-CD28^-$ cells | 25 | 0.684 | 0.153 | -1.247 | 33 | 35 | 8 | 27.25 |
| 24 | CM $CD8^+CD27^+CD28^+$ cells | 25 | 0.301 | 0.124 | 0.882 | 29 | 32 | 19 | 27.25 |
| 25 | miR-20a | 29 | 0.721 | 0.056 | -0.359 | 15 | 11 | 69 | 27.50 |
| 26 | TEMRA $CD4^+CD27^-CD28^-$ cells | 25 | 0.684 | 0.157 | -1.086 | 34 | 36 | 11 | 28.75 |
| 27 | CM $CD4^+CD27^+$ cells | 25 | 0.301 | 0.124 | 0.756 | 28 | 31 | 29 | 29.00 |
| 28 | $CD56^{bright}CD16^-$ NK-cells | 25 | 0.301 | 0.123 | 0.693 | 30 | 30 | 34 | 31.00 |
| 29 | TEMRA $CD8^+$ cells | 25 | 0.713 | 0.097 | -0.503 | 22 | 25 | 55 | 31.00 |
| 30 | sCD25 | 29 | 0.711 | 0.069 | -0.448 | 23 | 17 | 61 | 31.00 |
| 31 | CM $CD4^+CD27^+CD28^+$ cells | 25 | 0.309 | 0.140 | 0.753 | 31 | 34 | 31 | 31.75 |
| 32 | miR-195 | 29 | 0.711 | 0.069 | -0.426 | 24 | 18 | 62 | 32.00 |
| 33 | CM $CD4^+CD28^+$ cells | 25 | 0.331 | 0.194 | 0.809 | 41 | 46 | 21 | 37.25 |
| 34 | CM $CD4^+$ cells | 25 | 0.338 | 0.215 | 0.796 | 42 | 47 | 23 | 38.50 |
| 35 | Naive $CD8^+CD27^+$ cells | 25 | 0.324 | 0.175 | 0.592 | 36 | 38 | 46 | 39.00 |
| 36 | Naive $CD8^+CD28^+$ cells | 25 | 0.324 | 0.175 | 0.633 | 38 | 40 | 41 | 39.25 |
| 37 | Naive $CD8^+CD27^+CD28^+$ cells | 25 | 0.324 | 0.175 | 0.606 | 37 | 39 | 44 | 39.25 |

| | | | | | | | | | |
|---|---|---|---|---|---|---|---|---|---|
| 38 | CD4$^+$CD28$^+$ cells | 25 | 0.294 | 0.109 | 0.209 | 26 | 26 | 85 | 40.75 |
| 39 | CD56$^{dim}$CD16$^+$ NK-cells | 25 | 0.735 | 0.066 | -0.034 | 13 | 16 | 122 | 41.00 |
| 40 | miR-92a | 29 | 0.684 | 0.113 | -0.313 | 32 | 29 | 72 | 41.25 |
| 41 | TEMRA CD8$^+$CD28$^+$ cells | 25 | 0.324 | 0.175 | 0.542 | 39 | 41 | 49 | 42.00 |
| 42 | miR-17 | 29 | 0.676 | 0.130 | -0.490 | 40 | 33 | 57 | 42.50 |
| 43 | Class-switched memory B-cells | 25 | 0.676 | 0.175 | -0.405 | 35 | 37 | 66 | 43.25 |
| 44 | EM CD8$^+$CD28$^+$ cells | 25 | 0.338 | 0.215 | 0.582 | 44 | 49 | 47 | 46.00 |
| 45 | Plasmacytoid dendritic cells | 25 | 0.357 | 0.268 | 0.757 | 53 | 55 | 27 | 47.00 |
| 46 | miR-223 | 29 | 0.658 | 0.176 | -0.513 | 47 | 42 | 52 | 47.00 |
| 47 | miR-126 | 29 | 0.653 | 0.191 | -0.508 | 49 | 44 | 54 | 49.00 |
| 48 | Naive CD4$^+$CD57$^+$ cells | 25 | 0.640 | 0.288 | -0.801 | 58 | 59 | 22 | 49.25 |
| 49 | Hematopoietic stem cells | 25 | 0.357 | 0.262 | 0.636 | 52 | 53 | 40 | 49.25 |
| 50 | miR-424 | 29 | 0.347 | 0.191 | 0.502 | 50 | 45 | 56 | 50.25 |
| 51 | let-7e | 29 | 0.358 | 0.224 | 0.597 | 55 | 50 | 45 | 51.25 |
| 52 | CM CD4$^+$CD27$^-$CD28$^-$ cells | 25 | 0.621 | 0.323 | -0.827 | 64 | 64 | 20 | 53.00 |
| 53 | EM CD4$^+$ cells | 25 | 0.662 | 0.215 | -0.247 | 43 | 48 | 80 | 53.50 |
| 54 | Tregs | 25 | 0.357 | 0.268 | 0.537 | 54 | 56 | 51 | 53.75 |
| 55 | IP-10 | 29 | 0.658 | 0.179 | -0.218 | 46 | 43 | 83 | 54.50 |
| 56 | CD8$^+$ cells | 25 | 0.647 | 0.262 | -0.416 | 51 | 54 | 64 | 55.00 |
| 57 | EM CD8$^+$CD27$^+$CD28$^+$ cells | 25 | 0.371 | 0.322 | 0.677 | 62 | 63 | 35 | 55.50 |
| 58 | TEMRA CD4$^+$ cells | 25 | 0.640 | 0.288 | -0.449 | 56 | 57 | 60 | 57.25 |
| 59 | Naive CD8$^+$ cells | 25 | 0.368 | 0.315 | 0.483 | 60 | 61 | 58 | 59.75 |
| 60 | CD3$^+$ cells | 25 | 0.654 | 0.238 | -0.147 | 48 | 52 | 91 | 59.75 |
| 61 | miR-18a | 29 | 0.379 | 0.302 | 0.538 | 65 | 60 | 50 | 60.00 |
| 62 | IL-17A | 29 | 0.613 | 0.335 | -0.565 | 66 | 65 | 48 | 61.25 |
| 63 | TIM-3 | 29 | 0.611 | 0.353 | -0.630 | 69 | 69 | 42 | 62.25 |
| 64 | B-cells | 25 | 0.368 | 0.315 | 0.403 | 61 | 62 | 67 | 62.75 |
| 65 | EM CD8$^+$CD27$^-$CD28$^-$ cells | 25 | 0.640 | 0.288 | -0.249 | 57 | 58 | 79 | 62.75 |
| 66 | EM CD8$^+$CD27$^+$ cells | 25 | 0.397 | 0.440 | 0.648 | 73 | 76 | 39 | 65.25 |
| 67 | NK-cells | 25 | 0.625 | 0.344 | -0.306 | 63 | 66 | 73 | 66.25 |
| 68 | miR-155 | 29 | 0.639 | 0.233 | -0.105 | 59 | 51 | 102 | 67.75 |
| 69 | CM CD8$^+$CD57$^+$ cells | 25 | 0.426 | 0.580 | 0.791 | 82 | 84 | 24 | 68.00 |
| 70 | CRP | 29 | 0.389 | 0.353 | 0.292 | 67 | 67 | 74 | 68.75 |
| 71 | CM CD8$^+$CD27$^-$CD28$^-$ cells | 25 | 0.434 | 0.620 | 0.776 | 83 | 85 | 25 | 69.00 |
| 72 | EM CD8$^+$ cells | 25 | 0.390 | 0.406 | 0.358 | 70 | 70 | 70 | 70.00 |
| 73 | LAG-3 | 29 | 0.611 | 0.353 | -0.285 | 68 | 68 | 76 | 70.00 |
| 74 | CD4/CD8 Ratio | 25 | 0.390 | 0.406 | 0.286 | 71 | 71 | 75 | 72.00 |
| 75 | IL-17F | 29 | 0.474 | 0.514 | | 109 | 80 | 2 | 75.00 |
| 76 | miR-150 | 29 | 0.592 | 0.435 | -0.314 | 78 | 73 | 71 | 75.00 |
| 77 | CD4$^+$CD27$^+$CD28$^+$ cells | 25 | 0.397 | 0.440 | 0.210 | 72 | 75 | 84 | 75.75 |
| 78 | IL-1$\alpha$ | 29 | 0.592 | 0.435 | -0.284 | 77 | 74 | 77 | 76.25 |
| 79 | CD86 | 29 | 0.595 | 0.429 | -0.239 | 76 | 72 | 81 | 76.25 |
| 80 | 4-1BB | 29 | 0.542 | 0.566 | -0.474 | 93 | 83 | 59 | 82.00 |
| 81 | Naive Tregs | 25 | 0.596 | 0.475 | -0.108 | 75 | 78 | 100 | 82.00 |
| 82 | Naive CD8$^+$CD57$^+$ cells | 25 | 0.581 | 0.549 | -0.154 | 80 | 82 | 89 | 82.75 |

| | | | | | | | | | |
|---|---|---|---|---|---|---|---|---|---|
| 83 | miR-21 | 29 | 0.576 | 0.520 | -0.140 | 81 | 81 | 92 | 83.75 |
| 84 | PD-1 | 29 | 0.524 | 0.854 | 1.433 | 110 | 110 | 7 | 84.25 |
| 85 | miR-181a | 29 | 0.582 | 0.491 | 0.095 | 79 | 79 | 108 | 86.25 |
| 86 | Non-classical monocytes | 25 | 0.559 | 0.669 | -0.181 | 85 | 89 | 87 | 86.50 |
| 87 | EM CD8$^+$CD57$^+$ cells | 25 | 0.603 | 0.440 | -0.009 | 74 | 77 | 126 | 87.75 |
| 88 | CD4$^+$ cells | 25 | 0.434 | 0.628 | 0.107 | 84 | 86 | 101 | 88.75 |
| 89 | CTLA-4 | 29 | 0.492 | 0.931 | 2.020 | 122 | 121 | 3 | 92.00 |
| 90 | miR-19a | 29 | 0.458 | 0.731 | 0.147 | 94 | 92 | 90 | 92.50 |
| 91 | CM CD4$^+$CD57$^+$ cells | 25 | 0.551 | 0.705 | -0.098 | 88 | 90 | 107 | 93.25 |
| 92 | MCP-1 | 29 | 0.447 | 0.663 | 0.072 | 87 | 88 | 113 | 93.75 |
| 93 | sCD27 | 29 | 0.558 | 0.636 | 0.060 | 86 | 87 | 116 | 93.75 |
| 94 | IGF-1 | 29 | 0.542 | 0.735 | -0.113 | 92 | 93 | 99 | 94.00 |
| 95 | Naive CD4$^+$CD27$^-$CD28$^-$ cells | 25 | 0.507 | 0.977 | -1.714 | 125 | 123 | 5 | 94.50 |
| 96 | IL-1β | 29 | 0.545 | 0.713 | 0.092 | 89 | 91 | 109 | 94.50 |
| 97 | IL12p70 | 29 | 0.532 | 0.801 | 0.401 | 104 | 104 | 68 | 95.00 |
| 98 | EM CD4$^+$CD27$^+$ cells | 25 | 0.544 | 0.754 | -0.101 | 90 | 96 | 104 | 95.00 |
| 99 | CD4$^+$CD27$^+$ cells | 25 | 0.463 | 0.798 | 0.126 | 98 | 99 | 96 | 97.75 |
| 100 | TNF-α | 29 | 0.539 | 0.747 | 0.099 | 96 | 94 | 105 | 97.75 |
| 101 | TEMRA CD4$^+$CD27$^+$CD28$^+$ cells | 25 | 0.537 | 0.798 | -0.130 | 100 | 101 | 93 | 98.50 |
| 102 | let-7i | 29 | 0.524 | 0.854 | -0.405 | 111 | 111 | 65 | 99.50 |
| 103 | Naive CD4$^+$CD27$^+$ cells | 25 | 0.544 | 0.754 | -0.037 | 91 | 97 | 121 | 100.00 |
| 104 | EM CD4$^+$CD27$^+$CD28$^+$ cells | 25 | 0.537 | 0.798 | -0.103 | 99 | 100 | 103 | 100.25 |
| 105 | Non-switched memory B-cells | 25 | 0.529 | 0.842 | 0.228 | 106 | 109 | 82 | 100.75 |
| 106 | PD-L2 | 29 | 0.463 | 0.769 | 0.083 | 97 | 98 | 111 | 100.75 |
| 107 | Naive B-cells | 25 | 0.463 | 0.798 | 0.117 | 102 | 103 | 97 | 101.00 |
| 108 | TEMRA CD4$^+$CD27$^+$ cells | 25 | 0.533 | 0.816 | -0.127 | 103 | 105 | 94 | 101.25 |
| 109 | IL-8 | 29 | 0.461 | 0.748 | 0.010 | 95 | 95 | 124 | 102.25 |
| 110 | IL-10 | 29 | 0.518 | 0.890 | 0.425 | 116 | 116 | 63 | 102.75 |
| 111 | TEMRA CD4$^+$CD28$^+$ cells | 25 | 0.478 | 0.887 | 0.256 | 114 | 114 | 78 | 105.00 |
| 112 | Monocytes | 25 | 0.537 | 0.798 | 0.041 | 101 | 102 | 120 | 106.00 |
| 113 | Memory Tregs | 25 | 0.478 | 0.887 | 0.114 | 113 | 113 | 98 | 109.25 |
| 114 | miR-19b | 29 | 0.471 | 0.818 | -0.067 | 108 | 107 | 114 | 109.25 |
| 115 | miR-326 | 29 | 0.471 | 0.817 | -0.054 | 107 | 106 | 118 | 109.50 |
| 116 | Classical monocytes | 25 | 0.471 | 0.842 | 0.012 | 105 | 108 | 123 | 110.25 |
| 117 | IL-6 | 29 | 0.487 | 0.927 | 0.179 | 121 | 119 | 88 | 112.25 |
| 118 | EM CD4$^+$CD28$^+$ cells | 25 | 0.522 | 0.887 | -0.058 | 112 | 112 | 117 | 113.25 |
| 119 | PD-L1 | 29 | 0.516 | 0.909 | 0.099 | 118 | 118 | 106 | 115.00 |
| 120 | IL-27 | 29 | 0.505 | 0.982 | 0.196 | 126 | 127 | 86 | 116.25 |
| 121 | Naive CD4$^+$CD27$^+$CD28$^+$ cells | 25 | 0.493 | 0.977 | 0.127 | 123 | 124 | 95 | 116.25 |
| 122 | miR-125b | 29 | 0.484 | 0.909 | 0.046 | 117 | 117 | 119 | 117.50 |
| 123 | Naive CD4$^+$ cells | 25 | 0.522 | 0.887 | 0.010 | 115 | 115 | 125 | 117.50 |
| 124 | Naive CD4$^+$CD28$^+$ cells | 25 | 0.485 | 0.932 | 0.079 | 119 | 122 | 112 | 118.00 |
| 125 | Intermediate monocytes | 25 | 0.493 | 0.977 | 0.062 | 124 | 125 | 115 | 122.00 |
| 126 | Myeloid dendritic cells | 25 | 0.485 | 0.930 | -0.003 | 120 | 120 | 128 | 122.00 |
| 127 | miR-146a | 29 | 0.503 | 1.000 | -0.092 | 128 | 128 | 110 | 123.50 |

| | | | | | | | | | |
|---|---|---|---|---|---|---|---|---|---|
| 128 | IFN-γ | 29 | 0.505 | 0.982 | 0.006 | 127 | 126 | 127 | 126.75 |
