# Supplementary material for: Blood Immunosenescence Signatures Reflecting Age, Frailty and Tumor Immune Infiltrate in Patients with Early Luminal Breast Cancer
Source: Cancers (Basel). 2021 May 2;13(9):2185. doi: 10.3390/cancers13092185 (PMC8125302; doi:10.3390/cancers13092185)
Supplement: Supplementary file 1 [file cancers-13-02185-s001.zip › Table S3 - Individual performance_TILs.pdf]

Table S3: Individual performances of biomarkers correlating with sTIL infiltration (high, intermediate or low infiltration). The table reports the number of patients (N) for which the biomarkers could be measured, as well as the area under the curve (AUC) via receiver operating characteristics (ROC), P-value (Wilcox rank-sum test) and log fold change (FC) for each biomarker. The log FC compared case vs. control. A positive log FC indicates that the measurement is higher than its reference while a negative measurement indicates that is smaller. Based on these statistics AUC, P-value, log FC scores were computed. The final score combines the 3 scores, where AUC weighted double. The biomarkers are ranked based on their final score.

	Blood markers	N	AUC	*P*-value	log FC	AUC score	*P*-value score	log FC score	Final score
				**HIGH sTIL INFILTRATION**					
1	TIM-3	62	0.747	0.070	-0.908	2	24	20	12.00
2	TEMRA CD8^{+}CD27^{-}CD28^{-} cells	54	0.265	0.089	0.630	5	34	37	20.25
3	let-7i	62	0.739	0.081	-0.556	3	30	51	21.75
4	EM CD8^{+} cells	54	0.735	0.089	-0.549	4	35	55	24.50
5	Tumor grade	62	0.158	0.003	0.384	1	1	104	26.75
6	Tregs	54	0.696	0.156	-0.781	13	69	26	30.25
7	TEMRA CD8^{+} cells	54	0.269	0.095	0.480	6	38	72	30.50
8	TEMRA CD8^{+}CD27^{+} cells	54	0.271	0.098	0.495	7	39	70	30.75
9	miR-424	62	0.707	0.130	-0.587	10	59	47	31.50
10	TEMRA CD8^{+}CD57^{+} cells	54	0.282	0.116	0.514	8	47	67	32.50
11	let-7e	62	0.691	0.163	-0.606	14	72	43	35.75
12	EM CD4^{+}CD27^{-}CD28^{-} cells	54	0.324	0.205	1.466	25	89	10	37.25
13	sCD25	62	0.684	0.179	-0.591	18	80	46	40.50
14	miR-181a	62	0.679	0.192	-0.640	23	83	35	41.00
15	CTLA-4	62	0.363	0.120	1.529	68	48	9	48.25
16	miR-20a	62	0.295	0.134	0.355	12	62	115	50.25
17	TEMRA CD8^{+}CD27^{+}CD28^{+} cells	54	0.318	0.189	0.402	20	82	97	54.75
18	CD8^{+}CD27^{-}CD28^{-} cells	54	0.327	0.217	0.423	26	92	84	57.00
19	EM CD8^{+}CD28^{+} cells	54	0.665	0.241	-0.528	32	103	63	57.50
20	Hematopoietic stem cells	54	0.653	0.267	-0.750	45	117	28	58.75
21	Naive CD8^{+}CD27^{-}CD28^{-} cells	54	0.343	0.257	0.531	38	109	62	61.75
22	CD8^{+}CD28^{+} cells	54	0.669	0.229	-0.332	28	97	121	68.50
23	CD4^{+}CD57^{+} cells	54	0.359	0.321	0.789	63	132	24	70.50
24	CD86	62	0.656	0.255	-0.413	42	108	92	71.00
25	CD4^{+} Tregs	54	0.649	0.283	-0.510	50	120	68	72.00
26	CD4^{+}CD27^{-}CD28^{-} cells	54	0.363	0.325	0.988	69	134	17	72.25
27	TEMRA CD4^{+}CD27^{-}CD28^{-} cells	54	0.355	0.296	0.570	58	124	49	72.25
28	CM CD8^{+}CD57^{+} cells	54	0.333	0.227	0.308	30	96	133	72.25
29	NK-like T-cells	54	0.371	0.355	0.607	81	148	42	88.00
30	EM CD4^{+}CD28^{+} cells	54	0.645	0.306	-0.352	57	128	116	89.50
31	CD8^{+}CD57^{+} cells	54	0.347	0.279	0.270	46	119	149	90.00
32	TEMRA CD4^{+}CD57^{+} cells	54	0.376	0.371	0.552	84	155	54	94.25
33	Memory Tregs	54	0.351	0.292	0.267	51	123	155	95.00
34	Gal-9	62	0.621	0.379	-0.620	92	159	40	95.75
35	EM CD4^{+}CD57^{+} cells	54	0.384	0.403	1.136	105	170	11	97.75
36	Naive CD4^{+}CD27^{-}CD28^{-} cells	54	0.365	0.332	0.290	71	137	139	104.50
37	Naive Tregs	54	0.657	0.266	-0.147	39	114	252	111.00

38	IL12p70	62	0.611	0.423	-0.621	115	179	39	112.00
39	miR-195	62	0.393	0.438	1.116	125	189	13	113.00
40	CD4$^+$CD28$^+$ cells	54	0.645	0.296	-0.195	59	125	209	113.00
41	IL-6	62	0.614	0.408	-0.541	111	176	60	114.50
42	EM CD8$^+$CD27$^+$ cells	54	0.616	0.403	-0.452	106	171	75	114.50
43	EM CD8$^+$CD27$^+$CD28$^+$ cells	54	0.616	0.403	-0.403	107	173	95	120.50
44	IL-1α	62	0.619	0.386	-0.283	97	162	145	125.25
45	Non-switched memory B-cells	54	0.380	0.398	0.282	95	167	146	125.75
46	CM CD4$^+$CD27$^-$CD28$^-$ cells	54	0.392	0.418	0.419	122	178	90	128.00
47	CD8$^+$CD27$^+$CD28$^+$ cells	54	0.620	0.387	-0.235	94	163	179	132.50
48	CM CD8$^+$CD27$^+$ cells	54	0.335	0.233	0.023	31	98	374	133.50
49	CM CD8$^+$CD27$^+$CD28$^+$ cells	54	0.339	0.253	0.028	35	106	365	135.25
50	4-1BB	62	0.353	0.196	-0.038	53	84	354	136.00
51	Naive CD4$^+$CD57$^+$ cells	54	0.400	0.474	0.421	131	202	87	137.75
52	TEMRA CD4$^+$ cells	54	0.396	0.467	0.268	127	199	153	151.50
53	IL-17F	62	0.439	0.352	-3.561	232	147	2	153.25
54	Age	62	0.618	0.393	-0.154	100	165	248	153.25
55	miR-125b	62	0.389	0.423	0.195	117	180	208	155.50
56	MCP-1	62	0.611	0.423	-0.170	116	181	231	161.00
57	EM CD8$^+$CD57$^+$ cells	54	0.584	0.562	-0.438	168	236	78	162.50
58	Monocytes	54	0.388	0.432	0.159	113	187	242	163.75
59	Naive CD4$^+$CD27$^+$ cells	54	0.596	0.504	-0.228	139	212	183	168.25
60	Classical monocytes	54	0.367	0.350	0.018	76	146	377	168.75
61	CD8$^+$CD27$^+$ cells	54	0.600	0.474	-0.191	132	203	215	170.50
62	EM CD8$^+$CD27$^-$CD28$^-$ cells	54	0.576	0.591	-0.542	187	250	59	170.75
63	miR-155	62	0.430	0.614	0.966	202	266	19	172.25
64	miR-19a	62	0.591	0.510	-0.249	156	214	170	174.00
65	CM CD8$^+$CD28$^+$ cells	54	0.376	0.371	-0.019	85	156	375	175.25
66	NK-cells	54	0.616	0.415	-0.040	103	177	349	183.00
67	Naive CD8$^+$CD57$^+$ cells	54	0.404	0.492	0.155	141	209	246	184.25
68	Plasmacytoid dendritic cells	54	0.414	0.541	0.223	163	224	188	184.50
69	Naive CD4$^+$CD28$^+$ cells	54	0.584	0.551	-0.229	167	230	182	186.50
70	Non-classical monocytes	54	0.596	0.504	-0.142	140	213	256	187.25
71	CM CD8$^+$ cells	54	0.384	0.403	-0.019	104	172	376	189.00
72	Naive CD8$^+$CD28$^+$ cells	54	0.567	0.633	-0.517	215	273	65	192.00
73	miR-92a	62	0.423	0.578	0.260	182	244	160	192.00
74	miR-223	62	0.430	0.614	0.394	203	265	100	192.75
75	Naive CD4$^+$ cells	54	0.580	0.581	-0.236	177	245	178	194.25
76	Naive CD4$^+$CD27$^+$CD28$^+$ cells	54	0.580	0.581	-0.224	178	246	187	197.25
77	CRP	62	0.568	0.623	-0.376	209	271	108	199.25
78	Lymphnode involvement	62	0.563	0.601	-0.396	225	258	98	201.50
79	miR-126	62	0.426	0.596	0.259	194	255	163	201.50
80	CM CD4$^+$CD27$^+$CD28$^+$ cells	54	0.408	0.511	0.094	152	215	293	203.00
81	CM CD4$^+$CD27$^+$ cells	54	0.408	0.523	0.091	151	219	295	204.00
82	CD4$^+$CD27$^+$ cells	54	0.584	0.551	-0.124	164	229	267	206.00

83	CD4$^+$CD27$^+$CD28$^+$ cells	54	0.584	0.562	-0.127	165	234	265	207.25
84	EM CD4$^+$CD27$^+$ cells	54	0.576	0.602	-0.213	186	260	198	207.50
85	B-cells	54	0.592	0.523	-0.080	153	220	309	208.75
86	miR-9	62	0.547	0.694	1.057	265	292	14	209.00
87	IGF-1	62	0.582	0.552	-0.120	171	232	270	211.00
88	TNF-α	62	0.556	0.688	-0.449	245	288	77	213.75
89	miR-21	62	0.407	0.501	0.039	149	211	350	214.75
90	T-cell *P16^{INK4a}*	42	0.440	0.751	-0.476	235	318	73	215.25
91	Intermediate monocytes	54	0.592	0.523	-0.041	154	221	348	219.25
92	miR-326	62	0.561	0.607	-0.270	233	263	150	219.75
93	IL-1β	62	0.556	0.688	-0.384	244	289	105	220.50
94	EM CD4$^+$CD27$^+$CD28$^+$ cells	54	0.567	0.643	-0.222	214	276	190	223.50
95	Class-switched memory B-cells	54	0.569	0.622	-0.188	205	270	218	224.50
96	Tumor size	62	0.418	0.548	-0.059	172	228	326	224.50
97	Naive CD8$^+$CD27$^+$CD28$^+$ cells	54	0.547	0.750	-0.543	268	317	58	227.75
98	IP-10	62	0.565	0.642	-0.188	218	275	217	232.00
99	Naive CD8$^+$ cells	54	0.547	0.750	-0.422	267	316	86	234.00
100	CD56^{bright}CD16$^-$ NK-cells	54	0.557	0.687	-0.237	242	287	177	237.00
101	CM CD4$^+$ cells	54	0.416	0.562	0.012	166	235	389	239.00
102	Naive CD8$^+$CD27$^+$ cells	54	0.543	0.765	-0.525	285	324	64	239.50
103	IFN-γ	62	0.549	0.727	-0.285	263	306	144	244.00
104	Free active TGF-β1	62	0.518	0.704		351	295	1	249.50
105	sCD27	62	0.432	0.623	-0.073	210	272	313	251.25
106	CM CD4$^+$CD28$^+$ cells	54	0.424	0.602	0.011	185	259	390	254.75
107	LAG-3	62	0.530	0.836	-0.573	316	349	48	257.25
108	CD4$^+$ cells	54	0.551	0.728	-0.187	254	307	219	258.50
109	CM CD4$^+$CD57$^+$ cells	54	0.449	0.720	-0.165	255	300	237	261.75
110	IL-17A	62	0.481	0.897	-1.036	345	369	15	268.50
111	TEMRA CD4$^+$CD28$^+$ cells	54	0.555	0.707	-0.092	248	296	294	271.50
112	miR-18a	62	0.546	0.747	-0.169	278	315	232	275.75
113	PD-1	62	0.486	0.928	-0.968	363	378	18	280.50
114	miR-150	62	0.530	0.836	-0.268	318	348	152	284.00
115	miR-146a	62	0.556	0.689	-0.024	246	290	373	288.75
116	CD8$^+$ cells	54	0.465	0.817	0.176	300	342	228	292.50
117	TEMRA CD4$^+$CD27$^+$CD28$^+$ cells	54	0.547	0.743	-0.059	266	312	329	293.25
118	miR-17	62	0.460	0.776	0.059	288	332	325	308.25
119	CD56^{dim}CD16$^+$ NK-cells	54	0.457	0.772	0.042	284	327	344	309.75
120	TEMRA CD8$^+$CD28$^+$ cells	54	0.453	0.743	-0.008	269	313	395	311.50
121	PD-L1	62	0.544	0.756	0.026	281	320	369	312.75
122	IL-10	62	0.496	0.990	-0.410	392	395	93	318.00
123	miR-19b	62	0.460	0.776	-0.014	289	331	382	322.75
124	IL-27	62	0.516	0.918	-0.186	355	373	221	326.00
125	PD-L2	62	0.470	0.836	0.066	317	350	321	326.25
126	CM CD8$^+$CD27$^-$CD28$^-$ cells	54	0.467	0.823	0.038	305	345	352	326.75
127	IL-8	62	0.484	0.918	-0.157	356	374	243	332.25

128	CD4/CD8 Ratio	54	0.520	0.893	-0.062	342	367	323	343.50
129	TEMRA CD4$^+$CD27$^+$ cells	54	0.527	0.858	0.010	327	356	392	350.50
130	Naive B-cells	54	0.522	0.885	-0.032	340	365	360	351.25
131	CD3$^+$ cells	54	0.486	0.929	-0.027	360	379	366	366.25
132	EM CD4$^+$ cells	54	0.498	1.000	0.104	395	397	284	367.75
133	Myeloid dendritic cells	54	0.508	0.964	0.016	379	387	381	381.50
				**INTERMEDIATE sTIL INFILTRATION**					
1	PD-L1	62	0.313	0.018	0.704	15	5	31	16.50
2	TEMRA CD4$^+$CD57$^+$ cells	54	0.317	0.033	0.705	19	10	30	19.50
3	TEMRA CD4$^+$CD27$^-$CD28$^-$ cells	54	0.356	0.094	0.601	60	37	45	50.50
4	Intermediate monocytes	54	0.658	0.065	-0.360	36	21	111	51.00
5	CD8$^+$ cells	54	0.337	0.057	0.315	34	16	129	53.25
6	Tumor size	62	0.656	0.047	-0.351	43	13	117	54.00
7	CD4/CD8 ratio	54	0.631	0.127	-0.623	78	56	38	62.50
8	MCP-1	62	0.668	0.034	-0.228	29	11	185	63.50
9	CM CD8$^+$CD27$^+$CD28$^+$ cells	54	0.647	0.087	-0.296	54	32	136	69.00
10	Tregs	54	0.633	0.122	-0.380	75	50	107	76.75
11	EM CD8$^+$CD28$^+$ cells	54	0.633	0.123	-0.374	74	53	109	77.50
12	CM CD8$^+$CD28$^+$ cells	54	0.631	0.127	-0.374	77	55	110	79.75
13	G8 score	27	0.717	0.076	-0.104	9	29	285	83.00
14	CM CD8$^+$CD27$^+$ cells	54	0.641	0.101	-0.256	65	41	166	84.25
15	Memory Tregs	54	0.639	0.106	-0.252	67	43	168	86.25
16	miR-150	62	0.386	0.150	0.532	110	67	61	87.00
17	IGF-1	62	0.357	0.072	0.208	61	27	201	87.50
18	NK-like T-cells	54	0.381	0.165	0.429	98	74	81	87.75
19	CM CD8$^+$ cells	54	0.627	0.139	-0.307	83	65	134	91.25
20	miR-195	62	0.380	0.130	0.325	96	58	127	94.25
21	miR-9	62	0.408	0.178	0.729	155	78	29	104.25
22	T-cell $P16^{INK4a}$	42	0.369	0.162	0.196	79	71	206	108.75
23	Monocytes	54	0.372	0.137	0.183	82	64	224	113.00
24	CD4$^+$CD57$^+$ cells	54	0.390	0.201	0.301	119	87	135	115.00
25	miR-146a	62	0.398	0.201	0.288	129	85	142	121.25
26	CD56^{bright}CD16$^-$ NK-cells	54	0.390	0.202	0.265	120	88	157	121.25
27	Gal-9	62	0.594	0.239	-0.386	147	102	102	124.50
28	Naive CD4$^+$CD27$^-$CD28$^-$ cells	54	0.417	0.337	0.808	170	138	23	125.25
29	IL-6	62	0.590	0.259	-0.452	158	110	76	125.50
30	CD4$^+$ cells	54	0.610	0.201	-0.232	118	86	181	125.75
31	IL-17A	62	0.425	0.346	1.953	191	145	7	133.50
32	PD-1	62	0.402	0.219	0.245	135	93	173	134.00
33	EM CD4$^+$CD27$^-$CD28$^-$ cells	54	0.422	0.366	-0.647	181	153	33	137.00
34	CRP	62	0.589	0.264	-0.351	159	113	118	137.25
35	4-1BB	62	0.432	0.305	2.204	212	126	5	138.75
36	IL-8	62	0.609	0.170	-0.161	121	77	240	139.75
37	EM CD4$^+$CD27$^+$CD28$^+$ cells	54	0.404	0.267	0.216	138	115	195	146.50
38	TEMRA CD4$^+$ cells	54	0.413	0.318	0.286	161	130	143	148.75

39	EM $CD4^+CD27^+$ cells	54	0.405	0.275	0.216	145	118	194	150.50
40	$CD8^+CD28^+$ cells	54	0.598	0.259	-0.153	134	111	250	157.25
41	Age	62	0.594	0.237	-0.154	148	100	249	161.25
42	$CD4^+CD28^+$ cells	54	0.618	0.168	-0.024	99	75	372	161.25
43	$CD4^+CD27^-CD28^-$ cells	54	0.404	0.264	0.115	137	112	276	165.50
44	let-7i	62	0.432	0.395	0.392	213	166	101	173.25
45	LAG-3	62	0.436	0.424	0.431	221	182	79	175.75
46	IL-1α	62	0.582	0.306	-0.174	175	127	229	176.50
47	Classical monocytes	54	0.399	0.243	0.047	130	104	342	176.50
48	EM $CD4^+CD28^+$ cells	54	0.418	0.346	0.186	174	144	220	178.00
49	TIM-3	62	0.564	0.424	-0.421	222	183	88	178.75
50	IP-10	62	0.573	0.358	-0.226	195	149	186	181.25
51	TEMRA $CD8^+CD57^+$ cells	54	0.418	0.346	0.156	173	143	245	183.50
52	EM $CD4^+CD57^+$ cells	54	0.417	0.337	-0.121	169	140	268	186.50
53	Naive Tregs	54	0.412	0.309	0.088	160	129	299	187.00
54	TEMRA $CD8^+CD27^-CD28^-$ cells	54	0.424	0.376	0.169	183	157	233	189.00
55	miR-424	62	0.563	0.429	-0.288	226	186	140	194.50
56	$CD8^+CD27^-CD28^-$ cells	54	0.431	0.427	0.218	206	184	193	197.25
57	EM $CD8^+CD27^+CD28^+$ cells	54	0.562	0.473	-0.308	231	201	132	198.75
58	EM $CD8^+$ cells	54	0.572	0.406	-0.164	198	174	238	202.00
59	Plasmacytoid dendritic cells	54	0.552	0.545	-0.420	251	225	89	204.00
60	TEMRA $CD8^+$ cells	54	0.434	0.445	0.147	217	191	251	219.00
61	CM $CD8^+CD27^-CD28^-$ cells	54	0.454	0.595	0.487	276	254	71	219.25
62	Tumor grade	62	0.435	0.338	0.085	219	141	303	220.50
63	miR-126	62	0.442	0.465	0.194	238	198	210	221.00
64	NK-cells	54	0.428	0.406	0.048	199	175	338	227.75
65	TNF-α	62	0.430	0.381	-0.045	204	160	343	227.75
66	IL-1β	62	0.433	0.398	0.041	216	168	347	236.75
67	IL-17F	62	0.520	0.599	1.006	344	256	16	240.00
68	miR-21	62	0.568	0.391	-0.016	208	164	380	240.00
69	Naive $CD8^+CD27^-CD28^-$ cells	54	0.441	0.497	0.112	237	210	278	240.50
70	CM $CD8^+CD57^+$ cells	54	0.549	0.570	0.195	260	241	207	242.00
71	Naive $CD8^+CD57^+$ cells	54	0.436	0.462	-0.054	224	197	332	244.25
72	Free active TGF-β1	62	0.486	0.588	2.572	364	247	3	244.50
73	EM $CD8^+CD27^+$ cells	54	0.544	0.615	-0.268	283	267	151	246.00
74	TEMRA $CD8^+CD27^+$ cells	54	0.451	0.570	0.180	261	243	226	247.75
75	miR-223	62	0.445	0.488	0.096	247	207	291	248.00
76	IL-10	62	0.443	0.479	-0.057	243	204	330	255.00
77	$CD8^+CD57^+$ cells	54	0.453	0.593	0.166	274	253	234	258.75
78	Naive $CD4^+CD28^+$ cells	54	0.547	0.589	-0.155	270	248	247	258.75
79	CM $CD4^+$ cells	54	0.550	0.567	-0.097	256	239	290	260.25
80	CM $CD4^+CD28^+$ cells	54	0.550	0.567	-0.096	257	240	292	261.50
81	IL-27	62	0.537	0.646	-0.209	297	278	199	267.75
82	Non-classical monocytes	54	0.547	0.593	-0.108	272	252	281	269.25
83	$CD4^+$ Tregs	54	0.553	0.539	-0.037	249	223	356	269.25

84	Naive B-cells	54	0.547	0.593	-0.102	271	251	287	270.00
85	miR-92a	62	0.546	0.562	-0.083	275	237	305	273.00
86	EM CD4$^+$ cells	54	0.453	0.589	0.059	273	249	327	280.50
87	CTLA-4	62	0.511	0.832	-0.641	372	347	34	281.25
88	IL12p70	62	0.468	0.690	-0.182	308	291	225	283.00
89	Naive CD4$^+$ cells	54	0.539	0.658	-0.121	292	282	269	283.75
90	TEMRA CD8$^+$CD27$^+$CD28$^+$ cells	54	0.463	0.668	0.120	296	284	271	286.75
91	TEMRA CD4$^+$CD27$^+$ cells	54	0.451	0.570	0.005	259	242	398	289.50
92	Naive CD4$^+$CD27$^+$ cells	54	0.533	0.712	-0.132	307	299	260	293.25
93	Naive CD4$^+$CD57$^+$ cells	54	0.467	0.703	0.115	303	294	275	293.75
94	CD56^{dim}CD16$^+$ NK-cells	54	0.545	0.605	-0.028	280	262	364	296.50
95	EM CD8$^+$CD27$^-$CD28$^-$ cells	54	0.486	0.882	0.386	365	364	103	299.25
96	CD4$^+$CD27$^+$ cells	54	0.539	0.655	-0.033	291	281	359	305.50
97	miR-181a	62	0.528	0.729	-0.102	323	309	288	310.75
98	CM CD4$^+$CD27$^-$CD28$^-$ cells	54	0.496	0.969	-0.430	389	390	80	312.00
99	TEMRA CD4$^+$CD27$^+$CD28$^+$ cells	54	0.460	0.648	-0.009	290	279	393	313.00
100	Naive CD4$^+$CD27$^+$CD28$^+$ cells	54	0.526	0.768	-0.119	328	326	273	313.75
101	Myeloid dendritic cells	54	0.525	0.773	-0.130	333	328	262	314.00
102	let-7e	62	0.474	0.752	0.113	330	319	277	314.00
103	CM CD4$^+$CD27$^+$ cells	54	0.531	0.726	-0.052	311	304	335	315.25
104	TEMRA CD8$^+$CD28$^+$ cells	54	0.530	0.730	0.060	314	310	324	315.50
105	miR-19b	62	0.529	0.723	-0.068	322	302	318	316.00
106	miR-155	62	0.507	0.934	-0.328	380	380	126	316.50
107	CD4$^+$CD27$^+$CD28$^+$ cells	54	0.533	0.712	-0.027	306	298	368	319.50
108	miR-125b	62	0.532	0.695	0.027	310	293	367	320.00
109	CM CD4$^+$CD27$^+$CD28$^+$ cells	54	0.529	0.744	-0.052	321	314	334	322.50
110	CM CD4$^+$CD57$^+$ cells	54	0.494	0.955	0.288	384	385	141	323.50
111	TEMRA CD4$^+$CD28$^+$ cells	54	0.469	0.726	-0.018	312	305	378	326.75
112	CD8$^+$CD27$^+$ cells	54	0.527	0.759	-0.048	325	322	340	328.00
113	Class-switched memory B-cells	54	0.474	0.766	0.054	329	325	331	328.50
114	CD8$^+$CD27$^+$CD28$^+$ cells	54	0.523	0.794	-0.078	336	337	311	330.00
115	Hematopoietic stem cells	54	0.509	0.925	-0.198	376	377	204	333.25
116	miR-20a	62	0.528	0.729	-0.017	324	308	379	333.75
117	CD3$^+$ cells	54	0.529	0.737	-0.005	319	311	399	337.00
118	miR-19a	62	0.519	0.815	-0.069	347	341	317	338.00
119	CD86	62	0.483	0.840	-0.087	353	353	301	340.00
120	B-cells	54	0.518	0.839	-0.070	350	351	315	341.50
121	IFN-γ	62	0.480	0.803	-0.041	343	339	346	342.75
122	EM CD8$^+$CD57$^+$ cells	54	0.501	1.000	0.228	399	399	184	345.25
123	Naive CD8$^+$ cells	54	0.490	0.912	0.129	374	372	263	345.75
124	miR-326	62	0.516	0.819	0.039	354	343	351	350.50
125	Non-switched memory B-cells	54	0.504	0.971	0.160	390	391	241	353.00
126	Naive CD8$^+$CD27$^+$CD28$^+$ cells	54	0.494	0.956	0.138	385	386	257	353.25
127	Lymph node involvement	62	0.512	0.869	-0.067	368	361	319	354.00
128	sCD27	62	0.492	0.923	-0.089	378	375	298	357.25

129	miR-18a	62	0.514	0.862	-0.036	359	357	357	358.00
130	PD-L2	62	0.512	0.887	-0.051	369	366	337	360.25
131	Naive CD8$^+$CD27$^+$ cells	54	0.498	0.985	0.132	394	393	261	360.50
132	sCD25	62	0.501	0.994	-0.147	397	396	254	361.00
133	Naive CD8$^+$CD28$^+$ cells	54	0.499	1.000	0.135	398	398	258	363.00
134	miR-17	62	0.510	0.910	0.007	375	371	397	379.50
**LOW sTIL INFILTRATION**									
1	TEMRA CD4$^+$CD57$^+$ cells	54	0.707	0.011	-0.879	11	3	21	11.50
2	TEMRA CD4$^+$CD27$^-$CD28$^-$ cells	54	0.679	0.027	-0.785	22	7	25	19.00
3	Tregs	54	0.313	0.021	0.567	17	6	50	22.50
4	PD-L1	62	0.657	0.038	-0.674	40	12	32	31.00
5	EM CD8$^+$CD28$^+$ cells	54	0.324	0.029	0.496	24	8	69	31.25
6	NK-like T-cells	54	0.651	0.062	-0.637	47	19	36	37.25
7	miR-195	62	0.642	0.060	-0.758	62	18	27	42.25
8	CD4$^+$CD57$^+$ cells	54	0.648	0.068	-0.606	52	23	44	42.75
9	Intermediate monocytes	54	0.327	0.032	0.328	27	9	125	47.00
10	MCP-1	62	0.313	0.013	0.255	16	4	167	50.75
11	CD8$^+$ cells	54	0.658	0.051	-0.350	37	14	119	51.75
12	TEMRA CD8$^+$CD27$^-$CD28$^-$ cells	54	0.650	0.065	-0.408	48	20	94	52.50
13	TIM-3	62	0.365	0.075	0.609	72	28	41	53.25
14	TEMRA CD8$^+$CD57$^+$ cells	54	0.649	0.066	-0.343	49	22	120	60.00
15	CD4$^+$CD27$^-$CD28$^-$ cells	54	0.634	0.100	-0.544	73	40	57	60.75
16	EM CD8$^+$ cells	54	0.354	0.071	0.315	56	26	130	67.00
17	Tumor grade	62	0.664	0.010	-0.208	33	2	202	67.50
18	Naive CD4$^+$CD27$^-$CD28$^-$ cells	54	0.621	0.137	-0.869	93	63	22	67.75
19	CD4/CD8 ratio	54	0.376	0.126	0.555	86	54	52	69.50
20	Gal-9	62	0.377	0.105	0.515	88	42	66	71.00
21	4-1BB	62	0.607	0.089	-2.274	124	33	4	71.25
22	TEMRA CD8$^+$ cells	54	0.639	0.086	-0.320	66	31	128	72.75
23	Monocytes	54	0.653	0.058	-0.223	44	17	189	73.50
24	IL-6	62	0.383	0.123	0.554	101	52	53	76.75
25	CD8$^+$CD28$^+$ cells	54	0.354	0.071	0.244	55	25	174	77.25
26	miR-424	62	0.379	0.110	0.419	91	46	91	79.75
27	TEMRA CD8$^+$CD27$^+$ cells	54	0.624	0.128	-0.356	87	57	114	86.25
28	CD8$^+$CD27$^-$CD28$^-$ cells	54	0.622	0.133	-0.360	89	61	112	87.75
29	EM CD4$^+$CD57$^+$ cells	54	0.614	0.159	-0.426	109	70	82	92.50
30	EM CD4$^+$CD27$^-$CD28$^-$ cells	54	0.631	0.107	-0.232	80	44	180	96.00
31	Tumor size	62	0.384	0.121	0.331	108	49	123	97.00
32	CD4$^+$CD28$^+$ cells	54	0.344	0.054	0.086	41	15	302	99.75
33	G8 score	27	0.321	0.132	0.085	21	60	304	101.50
34	TEMRA CD4$^+$ cells	54	0.614	0.163	-0.359	112	73	113	102.50
35	CRP	62	0.398	0.179	0.422	128	79	85	105.00
36	CD4$^+$ cells	54	0.384	0.153	0.268	102	68	154	106.50
37	Age	62	0.378	0.108	0.185	90	45	223	112.00
38	Naive CD8$^+$CD27$^-$CD28$^-$ cells	54	0.607	0.187	-0.311	123	81	131	114.50

39	miR-150	62	0.595	0.212	-0.425	144	90	83	115.25
40	IL-1α	62	0.389	0.143	0.239	114	66	176	117.50
41	EM $CD4^+CD27^+CD28^+$ cells	54	0.404	0.238	0.402	142	101	96	120.25
42	T-cell *P16*^{INK4a}	42	0.641	0.123	-0.079	64	51	310	122.25
43	Classical monocytes	54	0.636	0.093	-0.048	70	36	339	128.75
44	IL-17A	62	0.574	0.329	-1.716	192	136	8	132.00
45	TEMRA $CD8^+CD27^+CD28^+$ cells	54	0.597	0.235	-0.261	136	99	159	132.50
46	miR-9	62	0.569	0.291	-1.123	207	122	12	137.00
47	$CD8^+CD57^+$ cells	54	0.595	0.244	-0.250	143	105	169	140.00
48	IGF-1	62	0.604	0.170	-0.156	126	76	244	143.00
49	$CD4^+$ Tregs	54	0.401	0.221	0.189	133	95	216	144.25
50	IP-10	62	0.414	0.254	0.260	162	107	162	148.25
51	IL-8	62	0.406	0.214	0.192	146	91	214	149.25
52	EM $CD8^+CD27^+$ cells	54	0.420	0.329	0.381	179	135	106	149.75
53	miR-181a	62	0.419	0.288	0.260	176	121	161	158.50
54	miR-126	62	0.576	0.319	-0.263	184	131	158	164.25
55	CM $CD8^+CD28^+$ cells	54	0.426	0.365	0.332	193	152	122	165.00
56	miR-146a	62	0.575	0.322	-0.259	190	133	164	169.25
57	CM $CD8^+$ cells	54	0.427	0.369	0.275	196	154	148	173.50
58	CM $CD8^+CD27^+CD28^+$ cells	54	0.425	0.358	0.249	188	150	171	174.25
59	miR-223	62	0.572	0.344	-0.222	200	142	192	183.50
60	$CD56^{bright}CD16^-$ NK-cells	54	0.578	0.337	-0.166	180	139	235	183.50
61	Naive $CD4^+CD28^+$ cells	54	0.429	0.384	0.213	201	161	197	190.00
62	Hematopoietic stem cells	54	0.439	0.452	0.396	234	196	99	190.75
63	Naive $CD4^+CD57^+$ cells	54	0.565	0.428	-0.266	220	185	156	195.25
64	Non-classical monocytes	54	0.425	0.358	0.144	189	151	255	196.00
65	CM $CD8^+CD27^+$ cells	54	0.432	0.403	0.216	211	169	196	196.75
66	CM $CD8^+CD27^-CD28^-$ cells	54	0.553	0.520	-0.463	250	217	74	197.75
67	PD-1	62	0.593	0.220	-0.003	150	94	400	198.50
68	sCD25	62	0.442	0.450	0.291	241	195	138	203.75
69	Naive $CD8^+CD57^+$ cells	54	0.590	0.267	-0.008	157	116	394	206.00
70	Memory Tregs	54	0.428	0.377	0.118	197	158	274	206.50
71	Naive $CD4^+CD27^+$ cells	54	0.438	0.447	0.193	229	194	213	216.25
72	Naive $CD4^+$ cells	54	0.438	0.447	0.185	228	193	222	217.75
73	$CD8^+CD27^+CD28^+$ cells	54	0.438	0.444	0.147	230	190	253	225.75
74	LAG-3	62	0.549	0.521	-0.256	262	218	165	226.75
75	EM $CD8^+CD27^+CD28^+$ cells	54	0.563	0.447	-0.125	227	192	266	228.00
76	IL-10	62	0.552	0.491	0.166	252	208	236	237.00
77	$CD4^+CD27^+$ cells	54	0.436	0.433	0.071	223	188	314	237.00
78	EM $CD4^+CD27^+$ cells	54	0.558	0.479	-0.128	240	206	264	237.50
79	$CD8^+CD27^+$ cells	54	0.441	0.470	0.107	236	200	282	238.50
80	Naive $CD4^+CD27^+CD28^+$ cells	54	0.449	0.535	0.180	253	222	227	238.75
81	$CD4^+CD27^+CD28^+$ cells	54	0.442	0.479	0.067	239	205	320	250.75
82	TNF-α	62	0.546	0.546	0.164	277	226	239	254.75
83	IL-27	62	0.462	0.615	0.244	293	268	175	257.25

84	Naive B-cells	54	0.450	0.547	0.102	258	227	289	258.00
85	CM CD4$^+$CD27$^-$CD28$^-$ cells	54	0.541	0.601	0.200	287	257	203	258.50
86	Non-switched memory B-cells	54	0.538	0.644	-0.248	294	277	172	259.25
87	CTLA-4	62	0.532	0.516	-0.194	309	216	212	261.50
88	miR-19a	62	0.455	0.551	0.135	279	231	259	262.00
89	B-cells	54	0.452	0.558	0.090	264	233	297	264.50
90	Free active TGF-β1	62	0.507	0.779	-2.067	382	333	6	275.75
91	CD86	62	0.467	0.669	0.194	304	285	211	276.00
92	IL-1β	62	0.544	0.565	0.069	282	238	316	279.50
93	EM CD4$^+$ cells	54	0.543	0.604	-0.090	286	261	296	282.25
94	Lymph node involvement	62	0.470	0.648	0.171	313	280	230	284.00
95	miR-20a	62	0.538	0.621	-0.105	295	269	283	285.50
96	Plasmacytoid dendritic cells	54	0.483	0.840	0.277	352	352	147	300.75
97	let-7e	62	0.464	0.641	0.053	298	274	333	300.75
98	CM CD4$^+$CD57$^+$ cells	54	0.523	0.785	-0.209	337	334	200	302.00
99	miR-326	62	0.466	0.609	0.041	302	264	345	303.25
100	CM CD8$^+$CD57$^+$ cells	54	0.514	0.867	-0.291	361	359	137	304.50
101	sCD27	62	0.529	0.711	0.103	320	297	286	305.75
102	EM CD8$^+$CD57$^+$ cells	54	0.470	0.720	-0.074	315	301	312	310.75
103	IL-17F	62	0.501	1.000	-0.544	401	401	56	314.75
104	miR-18a	62	0.473	0.725	0.083	326	303	306	315.25
105	Myeloid dendritic cells	54	0.474	0.758	0.109	332	321	279	316.00
106	TEMRA CD4$^+$CD27$^+$ cells	54	0.535	0.673	-0.008	299	286	396	320.00
107	IL12p70	62	0.495	0.954	0.330	387	384	124	320.50
108	miR-21	62	0.466	0.662	0.003	301	283	401	321.50
109	EM CD8$^+$CD27$^-$CD28$^-$ cells	54	0.486	0.867	-0.197	362	360	205	322.25
110	let-7i	62	0.488	0.880	-0.222	370	362	191	323.25
111	EM CD4$^+$CD28$^+$ cells	54	0.523	0.787	-0.059	338	336	328	335.00
112	miR-155	62	0.515	0.846	-0.108	358	354	280	337.50
113	CM CD4$^+$CD28$^+$ cells	54	0.482	0.827	0.081	348	346	308	337.50
114	Naive Tregs	54	0.524	0.773	-0.030	334	329	361	339.50
115	NK-cells	54	0.524	0.773	-0.030	335	330	362	340.50
116	Naive CD8$^+$CD28$^+$ cells	54	0.477	0.785	0.033	339	335	358	342.75
117	CM CD4$^+$ cells	54	0.484	0.855	0.082	357	355	307	344.00
118	CD56^{dim}CD16$^+$ NK-cells	54	0.474	0.760	0.010	331	323	391	344.00
119	TEMRA CD4$^+$CD27$^+$CD28$^+$ cells	54	0.519	0.819	0.029	346	344	363	349.75
120	CD3$^+$ cells	54	0.479	0.799	0.014	341	338	383	350.75
121	miR-19b	62	0.486	0.863	0.066	366	358	322	353.00
122	miR-92a	62	0.482	0.813	-0.012	349	340	388	356.50
123	IFN-γ	62	0.503	0.971	0.119	393	392	272	362.50
124	Naive CD8$^+$CD27$^+$ cells	54	0.487	0.881	0.038	367	363	353	362.50
125	TEMRA CD8$^+$CD28$^+$ cells	54	0.489	0.902	-0.051	373	370	336	363.00
126	miR-125b	62	0.505	0.948	-0.087	386	383	300	363.75
127	Naive CD8$^+$CD27$^+$CD28$^+$ cells	54	0.489	0.896	0.037	371	368	355	366.25
128	TEMRA CD4$^+$CD28$^+$ cells	54	0.509	0.924	0.048	377	376	341	367.75

129	Naive $CD8^+$ cells	54	0.493	0.937	0.013	381	381	386	382.25
130	CM $CD4^+CD27^+CD28^+$ cells	54	0.506	0.944	0.013	383	382	387	383.75
131	miR-17	62	0.504	0.966	-0.025	391	389	371	385.50
132	CM $CD4^+CD27^+$ cells	54	0.504	0.965	0.013	388	388	384	387.00
133	PD-L2	62	0.498	0.989	0.025	396	394	370	389.00
134	Class-switched memory B-cells	54	0.499	1.000	0.013	400	400	385	396.25
