# Supplementary material for: Blood Immunosenescence Signatures Reflecting Age, Frailty and Tumor Immune Infiltrate in Patients with Early Luminal Breast Cancer
Source: Cancers (Basel). 2021 May 2;13(9):2185. doi: 10.3390/cancers13092185 (PMC8125302; doi:10.3390/cancers13092185)
Supplement: Supplementary file 1 [file cancers-13-02185-s001.zip › Table S4 - Individual performance_CD3 infiltration whole tumor.pdf]

Table S4: Individual performances of biomarkers correlating with CD3 infiltration in the whole tumor (high, intermediate or low infiltration). The table reports the number of patients (N) for which the biomarkers could be measured. The area under the curve (AUC) via receiver operating characteristics (ROC), P-value (Wilcox rank-sum test) and log fold change (FC) are reported for each biomarker. The log FC compared case vs. control. A positive log FC indicates that the measurement is higher than its reference while a negative measurement indicates that is smaller. Based on these statistics AUC, P-value, log FC scores were computed. The final score combines the 3 scores, where AUC weighted double. The biomarkers are ranked based on their final score.

	Blood markers	N	AUC	*P*-value	log FC	AUC score	*P*-value score	log FC score	Final score
**HIGH CD3 INFILTRATION**									
1	Tumor grade	61	0.207	0.000	0.351	1	1	116	29.75
2	Gal-9	61	0.668	0.052	-0.707	30	27	48	33.75
3	Age	61	0.753	0.004	-0.328	3	2	127	33.75
4	miR-195	61	0.346	0.077	0.964	40	36	25	35.25
5	IL-1α	61	0.703	0.019	-0.420	16	16	96	36
6	TEMRA $CD8^+CD27^+$ cells	53	0.312	0.040	0.473	21	22	83	36.75
7	TIM-3	61	0.648	0.089	-0.542	42	41	70	48.75
8	miR-20a	61	0.324	0.043	0.342	26	24	122	49.5
9	miR-326	61	0.620	0.105	-0.921	69	48	26	53
10	TEMRA $CD8^+CD27^+CD28^+$ cells	53	0.355	0.113	0.405	44	55	102	61.25
11	miR-150	61	0.372	0.143	0.617	62	66	57	61.75
12	sCD25	61	0.635	0.122	-0.355	51	57	114	68.25
13	miR-9	61	0.398	0.173	1.553	99	76	12	71.5
14	4-1BB	61	0.401	0.172	2.494	106	74	4	72.5
15	IL-17A	61	0.394	0.224	2.293	95	102	5	74.25
16	Intermediate monocytes	53	0.658	0.085	-0.228	37	38	186	74.5
17	miR-126	61	0.386	0.189	0.469	75	82	84	79
18	EM $CD4^+CD27^-CD28^-$ cells	53	0.386	0.215	0.483	78	98	81	83.75
19	miR-424	61	0.617	0.178	-0.358	72	79	112	83.75
20	IL-6	61	0.609	0.212	-0.539	89	96	71	86.25
21	miR-223	61	0.387	0.194	0.357	80	86	113	89.75
22	miR-125b	61	0.388	0.200	0.337	83	88	124	94.5
23	miR-19b	61	0.380	0.167	0.236	67	72	175	95.25
24	Naive $CD8^+CD27^-CD28^-$ cells	53	0.368	0.149	0.186	55	67	215	98
25	TEMRA $CD4^+CD27^+$ cells	53	0.394	0.246	0.343	93	108	120	103.5
26	TEMRA $CD4^+CD27^+CD28^+$ cells	53	0.399	0.272	0.342	103	116	121	110.75
27	Naive $CD8^+CD57^+$ cells	53	0.368	0.149	0.132	56	68	271	112.75
28	IFN-γ	61	0.588	0.314	-0.454	134	133	86	121.75
29	G8 score	26	0.688	0.401	-0.139	22	188	259	122.75
30	LAG-3	61	0.417	0.348	0.602	146	149	58	124.75
31	TEMRA $CD4^+CD27^-CD28^-$ cells	53	0.412	0.338	-0.411	133	144	99	127.25
32	IL-27	61	0.586	0.323	-0.367	138	136	110	130.5
33	IGF-1	61	0.407	0.291	0.214	116	122	193	136.75
34	IL12p70	61	0.577	0.379	-0.623	165	168	55	138.25
35	PD-L1	61	0.426	0.398	0.920	173	182	27	138.75
36	$CD56^{bright}CD16^-$ NK-cells	53	0.409	0.323	0.234	121	137	180	139.75
37	EM $CD8^+$ cells	53	0.590	0.332	-0.196	127	140	208	150.5

38	CD8^{+} cells	53	0.410	0.332	0.197	128	141	207	151
39	TEMRA CD8^{+}CD57^{+} cells	53	0.414	0.352	0.232	139	152	183	153.25
40	miR-18a	61	0.417	0.340	0.223	143	145	190	155.25
41	CM CD8^{+}CD57^{+} cells	53	0.421	0.391	0.293	155	176	146	158
42	CTLA-4	61	0.438	0.270	0.402	208	115	104	158.75
43	EM CD8^{+}CD28^{+} cells	53	0.581	0.384	-0.232	148	173	182	162.75
44	Tregs	53	0.579	0.391	-0.249	157	177	164	163.75
45	IP-10	61	0.580	0.357	-0.200	149	155	203	164
46	Monocytes	53	0.407	0.312	0.108	115	132	296	164.5
47	TNF-α	61	0.565	0.455	-0.598	198	210	60	166.5
48	EM CD4^{+}CD27^{+}CD28^{+} cells	53	0.421	0.396	0.224	154	181	188	169.25
49	CD4^{+}CD28^{+} cells	53	0.608	0.238	-0.009	90	106	393	169.75
50	EM CD4^{+}CD27^{+} cells	53	0.423	0.407	0.221	164	190	191	177.25
51	miR-19a	61	0.422	0.375	0.171	160	165	231	179
52	Hematopoietic stem cells	53	0.565	0.478	-0.408	200	224	101	181.25
53	Classical monocytes	53	0.407	0.312	0.024	114	131	366	181.25
54	MCP-1	61	0.580	0.357	-0.118	150	154	285	184.75
55	let-7e	61	0.564	0.466	-0.331	204	220	126	188.5
56	TEMRA CD4^{+}CD57^{+} cells	53	0.439	0.506	-0.423	213	235	94	188.75
57	CM CD4^{+}CD27^{-}CD28^{-} cells	53	0.429	0.417	-0.197	180	192	206	189.5
58	TEMRA CD8^{+} cells	53	0.421	0.391	0.130	156	178	276	191.5
59	PD-1	61	0.440	0.492	0.386	219	229	105	193
60	NK-like T-cells	53	0.446	0.558	0.689	241	254	49	196.25
61	miR-92a	61	0.436	0.461	0.251	203	216	163	196.25
62	IL-17F	61	0.464	0.398	1.595	296	184	11	196.75
63	sCD27	61	0.562	0.481	-0.247	209	225	167	202.5
64	Tumor size	61	0.567	0.437	-0.169	193	203	232	205.25
65	CD8^{+}CD57^{+} cells	53	0.432	0.466	0.160	191	219	239	210
66	miR-17	61	0.436	0.466	0.192	205	221	210	210.25
67	Free active TGF-β1	61	0.477	0.400	3.118	334	187	3	214.5
68	miR-146a	61	0.434	0.451	0.111	197	207	292	223.25
69	CD4^{+}CD27^{-}CD28^{-} cells	53	0.423	0.402	0.016	163	189	380	223.75
70	CM CD4^{+}CD27^{+}CD28^{+} cells	53	0.442	0.532	0.202	228	245	201	225.5
71	CM CD4^{+}CD27^{+} cells	53	0.445	0.556	0.201	239	253	202	233.25
72	TEMRA CD8^{+}CD27^{-}CD28^{-} cells	53	0.444	0.545	0.176	234	249	222	234.75
73	CD4/CD8 ratio	53	0.545	0.628	-0.280	261	270	151	235.75
74	CM CD8^{+}CD27^{+} cells	53	0.458	0.650	0.316	274	281	135	241
75	Naive CD4^{+}CD28^{+} cells	53	0.555	0.552	-0.157	238	252	243	242.75
76	Class-switched memory B-cells	53	0.444	0.545	0.139	235	250	262	245.5
77	miR-21	61	0.456	0.615	0.205	263	264	200	247.5
78	CD86	61	0.542	0.637	-0.240	275	274	169	248.25
79	CD8^{+}CD28^{+} cells	53	0.559	0.529	-0.100	226	243	302	249.25
80	CM CD8^{+}CD27^{-}CD28^{-} cells	53	0.438	0.499	-0.043	210	232	351	250.75
81	CM CD8^{+}CD27^{+}CD28^{+} cells	53	0.462	0.683	0.287	288	300	148	256
82	Naive CD4^{+}CD27^{+}CD28^{+} cells	53	0.546	0.625	-0.147	257	268	252	258.5

83	Naive $CD4^+$ cells	53	0.546	0.625	-0.137	256	267	263	260.5
84	Non-classical monocytes	53	0.560	0.516	-0.011	217	238	389	265.25
85	TEMRA $CD4^+CD28^+$ cells	53	0.462	0.683	0.198	286	298	205	268.75
86	CRP	61	0.539	0.660	-0.173	282	285	228	269.25
87	Naive $CD4^+CD27^-CD28^-$ cells	53	0.447	0.565	-0.048	242	256	347	271.75
88	Myeloid dendritic cells	53	0.460	0.664	0.155	278	289	244	272.25
89	B-cells	53	0.553	0.569	-0.050	243	258	345	272.25
90	Naive $CD4^+CD27^+$ cells	53	0.544	0.639	-0.117	266	275	286	273.25
91	CM $CD8^+CD28^+$ cells	53	0.464	0.701	0.182	292	306	218	277
92	$CD4^+$ cells	53	0.544	0.639	-0.095	267	276	307	279.25
93	IL-10	61	0.527	0.763	-0.249	323	327	165	284.5
94	Naive B-cells	53	0.544	0.639	-0.061	268	277	334	286.75
95	$CD8^+CD27^-CD28^-$ cells	53	0.462	0.683	0.112	287	299	290	290.75
96	T-cell $P16^{INK4a}$	41	0.508	0.952	-0.756	376	380	43	293.75
97	PD-L2	61	0.538	0.673	-0.083	290	291	315	296.5
98	Lymph node involvement	61	0.528	0.721	-0.161	319	312	238	297
99	$CD4^+CD27^+$ cells	53	0.462	0.679	0.063	285	296	332	299.5
100	IL-8	61	0.539	0.657	-0.036	283	283	356	301.25
101	CM $CD8^+$ cells	53	0.476	0.801	0.200	333	342	204	303
102	NK-cells	53	0.538	0.683	-0.061	289	301	333	303
103	IL-1β	61	0.514	0.873	-0.341	366	362	123	304.25
104	EM $CD8^+CD27^+$ cells	53	0.467	0.724	-0.108	306	313	294	304.75
105	$CD4^+$ Tregs	53	0.540	0.664	0.011	279	288	388	308.5
106	CM $CD4^+$ cells	53	0.471	0.757	0.116	313	323	287	309
107	CM $CD4^+CD28^+$ cells	53	0.474	0.788	0.119	330	335	284	319.75
108	$CD4^+CD27^+CD28^+$ cells	53	0.473	0.772	0.050	321	330	343	328.75
109	let-7i	61	0.529	0.744	-0.023	315	318	369	329.25
110	$CD4^+CD57^+$ cells	53	0.473	0.772	-0.050	322	331	344	329.75
111	miR-155	61	0.497	0.980	0.253	391	392	162	334
112	EM $CD4^+CD57^+$ cells	53	0.499	1.000	0.313	400	400	137	334.25
113	EM $CD8^+CD27^+CD28^+$ cells	53	0.482	0.850	-0.123	352	355	280	334.75
114	Plasmacytoid dendritic cells	53	0.501	1.000	-0.275	401	401	154	339.25
115	Naive $CD4^+CD57^+$ cells	53	0.478	0.817	-0.053	339	346	341	341.25
116	$CD8^+CD27^+$ cells	53	0.482	0.848	0.076	351	354	320	344
117	EM $CD8^+CD57^+$ cells	53	0.474	0.788	-0.014	331	336	381	344.75
118	EM $CD4^+$ cells	53	0.485	0.880	0.106	363	366	297	347.25
119	$CD8^+CD27^+CD28^+$ cells	53	0.480	0.832	0.035	347	351	358	350.75
120	Non-switched memory B-cells	53	0.478	0.819	-0.013	341	348	383	353.25
121	TEMRA $CD8^+CD28^+$ cells	53	0.514	0.888	-0.091	368	371	310	354.25
122	$CD56^{dim}CD16^+$ NK-cells	53	0.522	0.819	-0.005	340	347	398	356.25
123	EM $CD8^+CD27^-CD28^-$ cells	53	0.506	0.952	-0.116	380	381	288	357.25
124	EM $CD4^+CD28^+$ cells	53	0.518	0.850	0.022	353	356	371	358.25
125	CM $CD4^+CD57^+$ cells	53	0.484	0.864	-0.018	357	360	377	362.75
126	Naive $CD8^+CD27^+$ cells	53	0.495	0.960	-0.097	383	384	303	363.25
127	TEMRA $CD4^+$ cells	53	0.493	0.944	-0.071	377	377	324	363.75

128	Naive $CD8^+CD27^+CD28^+$ cells	53	0.496	0.976	-0.110	388	389	293	364.5
129	Naive $CD8^+$ cells	53	0.507	0.944	-0.057	378	378	339	368.25
130	Naive $CD8^+CD28^+$ cells	53	0.504	0.976	-0.083	389	388	313	369.75
131	Memory Tregs	53	0.489	0.913	0.031	374	375	362	371.25
132	$CD3^+$ cells	53	0.493	0.944	0.009	379	376	391	381.25
133	Naive Tregs	53	0.505	0.960	-0.006	384	386	397	387.75
134	miR-181a	61	0.499	0.993	0.033	397	399	360	388.25
	**INTERMEDIATE CD3 INFILTRATION**								
1	Naive $CD8^+CD57^+$ cells	53	0.720	0.006	-1.093	8	3	20	9.75
2	CM $CD8^+CD27^+CD28^+$ cells	53	0.712	0.007	-1.048	11	7	23	13
3	CM $CD8^+CD27^+$ cells	53	0.711	0.009	-1.048	12	10	24	14.5
4	Naive $CD8^+CD27^-CD28^-$ cells	53	0.717	0.007	-0.757	9	5	42	16.25
5	CM $CD8^+CD28^+$ cells	53	0.710	0.009	-0.900	13	11	31	17
6	T-cell *P16*^{INK4a}	41	0.306	0.034	1.532	18	21	14	17.75
7	CM $CD8^+$ cells	53	0.705	0.011	-0.879	14	13	32	18.25
8	CM $CD8^+CD57^+$ cells	53	0.713	0.008	-0.746	10	8	45	18.25
9	miR-92a	61	0.679	0.017	-0.496	24	14	79	35.25
10	CM $CD4^+CD27^+CD28^+$ cells	53	0.635	0.093	-0.596	50	43	61	51
11	CM $CD4^+CD27^+$ cells	53	0.634	0.096	-0.595	54	46	62	54
12	CM $CD4^+$ cells	53	0.628	0.112	-0.554	59	53	67	59.5
13	CM $CD4^+CD28^+$ cells	53	0.628	0.112	-0.553	60	54	68	60.5
14	miR-181a	61	0.626	0.093	-0.440	63	44	88	64.5
15	miR-326	61	0.403	0.125	0.825	108	58	37	77.75
16	CM $CD4^+CD57^+$ cells	53	0.610	0.173	-0.562	87	77	64	78.75
17	CM $CD8^+CD27^-CD28^-$ cells	53	0.635	0.094	-0.240	52	45	170	79.75
18	miR-17	61	0.614	0.128	-0.325	77	59	129	85.5
19	miR-223	61	0.610	0.141	-0.343	86	64	119	88.75
20	PD-1	61	0.404	0.201	0.618	110	90	56	91.5
21	IL-1α	61	0.372	0.086	0.190	58	39	212	91.75
22	Gal-9	61	0.386	0.129	0.237	76	60	174	96.5
23	miR-150	61	0.596	0.202	-0.499	111	91	78	97.75
24	$CD56^{bright}CD16^-$ NK-cells	53	0.606	0.188	-0.318	94	81	132	100.25
25	CRP	61	0.600	0.183	-0.346	104	80	118	101.5
26	Tumor grade	61	0.613	0.074	-0.148	81	33	250	111.25
27	IL-27	61	0.381	0.111	0.140	70	50	257	111.75
28	Naive $CD4^+CD57^+$ cells	53	0.588	0.274	-0.519	131	118	75	113.75
29	CD86	61	0.410	0.230	0.385	124	105	106	114.75
30	miR-146a	61	0.591	0.223	-0.323	118	101	130	116.75
31	CTLA-4	61	0.567	0.169	-1.402	196	73	16	120.25
32	EM $CD4^+CD57^+$ cells	53	0.401	0.220	0.225	107	100	187	125.25
33	EM $CD8^+CD27^+$ cells	53	0.610	0.173	-0.140	88	78	258	128
34	NK-cells	53	0.409	0.261	0.234	119	114	179	132.75
35	IFN-γ	61	0.410	0.227	0.232	123	104	181	132.75
36	Naive B-cells	53	0.593	0.253	-0.215	117	110	192	134
37	Age	61	0.388	0.135	0.093	82	63	309	134

38	Lymph node involvement	61	0.426	0.257	0.422	175	111	95	139
39	IL-17F	61	0.553	0.141	-7.536	245	65	2	139.25
40	PD-L1	61	0.415	0.257	-0.239	140	112	171	140.75
41	TEMRA $CD4^+CD57^+$ cells	53	0.429	0.378	0.761	181	167	41	142.5
42	EM $CD8^+CD27^+CD28^+$ cells	53	0.603	0.205	-0.120	98	93	283	143
43	$CD8^+CD27^+$ cells	53	0.588	0.274	-0.211	132	119	194	144.25
44	$CD4^+CD27^+$ cells	53	0.591	0.259	-0.153	120	113	248	150.25
45	TIM-3	61	0.394	0.156	-0.029	92	70	363	154.25
46	TEMRA $CD8^+CD27^+$ cells	53	0.582	0.310	-0.209	147	129	195	154.5
47	let-7e	61	0.574	0.327	-0.291	174	139	147	158.5
48	miR-424	61	0.424	0.309	0.223	166	128	189	162.25
49	$CD8^+CD27^+CD28^+$ cells	53	0.580	0.323	-0.186	151	138	214	163.5
50	$CD4^+CD27^+CD28^+$ cells	53	0.584	0.301	-0.145	142	124	255	165.75
51	IL-10	61	0.428	0.337	0.248	178	143	166	166.25
52	IL12p70	61	0.431	0.356	0.268	188	153	155	171
53	TEMRA $CD4^+CD27^-CD28^-$ cells	53	0.440	0.460	0.806	222	214	39	174.25
54	CD4/CD8 ratio	53	0.570	0.388	-0.235	187	175	178	181.75
55	EM $CD8^+CD27^-CD28^-$ cells	53	0.439	0.455	0.429	216	209	91	183
56	$CD8^+$ cells	53	0.423	0.344	0.133	162	148	270	185.5
57	miR-155	61	0.559	0.436	-0.433	227	202	89	186.25
58	miR-20a	61	0.573	0.334	-0.142	177	142	256	188
59	TEMRA $CD8^+CD28^+$ cells	53	0.435	0.423	0.237	201	197	173	193
60	miR-21	61	0.562	0.411	-0.257	211	191	160	193.25
61	miR-126	61	0.560	0.423	-0.255	218	196	161	198.25
62	IL-1β	61	0.433	0.370	0.153	194	162	247	199.25
63	IGF-1	61	0.567	0.378	-0.147	195	166	253	202.25
64	4-1BB	61	0.540	0.528	-1.661	281	241	9	203
65	TNF-α	61	0.430	0.351	0.104	186	150	298	205
66	$CD4^+CD27^-CD28^-$ cells	53	0.449	0.533	0.431	250	246	90	209
67	Naive $CD4^+CD28^+$ cells	53	0.432	0.398	0.126	189	183	278	209.75
68	TEMRA $CD4^+$ cells	53	0.449	0.531	0.408	249	244	100	210.5
69	Non-switched memory B-cells	53	0.440	0.463	0.236	224	218	177	210.75
70	EM $CD4^+CD27^+CD28^+$ cells	53	0.557	0.485	-0.239	233	228	172	216.5
71	Monocytes	53	0.561	0.453	-0.171	214	208	230	216.5
72	Plasmacytoid dendritic cells	53	0.447	0.516	0.303	244	237	142	216.75
73	EM $CD4^+CD27^+$ cells	53	0.556	0.496	-0.229	236	230	185	221.75
74	$CD4^+$ Tregs	53	0.560	0.460	-0.168	220	213	234	221.75
75	Tumor size	61	0.435	0.386	-0.037	202	174	353	232.75
76	sCD25	61	0.432	0.370	0.005	192	160	400	236
77	IP-10	61	0.437	0.399	0.042	207	186	352	238
78	Naive $CD4^+$ cells	53	0.440	0.463	0.104	221	217	300	239.75
79	TEMRA $CD8^+CD27^+CD28^+$ cells	53	0.560	0.460	-0.090	223	215	311	243
80	miR-18a	61	0.551	0.502	-0.162	251	234	237	243.25
81	Naive $CD4^+CD27^+CD28^+$ cells	53	0.443	0.485	0.121	232	227	282	243.25
82	Naive $CD4^+CD27^+$ cells	53	0.443	0.485	0.096	231	226	306	248.5

83	let-7i	61	0.561	0.419	0.021	215	194	375	249.75
84	CD4$^+$CD57$^+$ cells	53	0.466	0.678	0.362	301	295	111	252
85	CM CD4$^+$CD27$^-$CD28$^-$ cells	53	0.522	0.780	-1.057	338	332	22	257.5
86	Naive CD4$^+$CD27$^-$CD28$^-$ cells	53	0.552	0.522	-0.096	246	240	305	259.25
87	miR-19a	61	0.544	0.559	-0.147	265	255	254	259.75
88	MCP-1	61	0.555	0.466	-0.056	240	222	340	260.5
89	Class-switched memory B-cells	53	0.454	0.575	0.104	259	259	299	269
90	CD56^{dim}CD16$^+$ NK-cells	53	0.444	0.496	0.022	237	231	373	269.5
91	Memory Tregs	53	0.547	0.566	-0.083	254	257	314	269.75
92	miR-9	61	0.517	0.795	-0.855	355	337	35	270.5
93	EM CD4$^+$CD27$^-$CD28$^-$ cells	53	0.450	0.539	0.067	252	248	330	270.5
94	CD8$^+$CD27$^-$CD28$^-$ cells	53	0.460	0.627	0.132	280	269	273	275.5
95	TEMRA CD4$^+$CD27$^+$CD28$^+$ cells	53	0.534	0.676	-0.191	302	294	211	277.25
96	Tregs	53	0.533	0.682	-0.184	304	297	217	280.5
97	miR-125b	61	0.534	0.649	-0.139	300	280	261	285.25
98	TEMRA CD4$^+$CD27$^+$ cells	53	0.532	0.695	-0.175	308	304	225	286.25
99	EM CD4$^+$CD28$^+$ cells	53	0.456	0.590	0.036	264	262	357	286.75
100	EM CD8$^+$ cells	53	0.464	0.665	0.132	294	290	272	287.5
101	EM CD8$^+$CD57$^+$ cells	53	0.530	0.718	0.158	311	311	241	293.5
102	sCD27	61	0.529	0.704	-0.154	314	307	246	295.25
103	Free active TGF-β1	61	0.501	0.981	-1.549	399	394	13	301.25
104	IL-17A	61	0.498	0.988	-1.227	396	397	18	301.75
105	CD4$^+$CD28$^+$ cells	53	0.536	0.663	-0.057	293	287	338	302.75
106	IL-8	61	0.537	0.629	-0.023	291	271	370	305.75
107	LAG-3	61	0.495	0.949	-0.415	385	379	97	311.5
108	G8 score	26	0.542	0.729	-0.009	271	314	392	312
109	NK-like T-cells	53	0.511	0.901	-0.317	375	373	133	314
110	miR-195	61	0.511	0.885	-0.261	373	370	159	318.75
111	TEMRA CD8$^+$ cells	53	0.474	0.756	0.093	327	322	308	321
112	TEMRA CD8$^+$CD27$^-$CD28$^-$ cells	53	0.474	0.749	0.080	324	320	317	321.25
113	Naive CD8$^+$CD27$^+$CD28$^+$ cells	53	0.521	0.798	0.136	344	339	266	323.25
114	Myeloid dendritic cells	53	0.484	0.852	0.167	359	357	236	327.75
115	Naive CD8$^+$CD27$^+$ cells	53	0.521	0.803	0.126	346	343	277	328
116	CD3$^+$ cells	53	0.474	0.749	0.045	325	319	348	329.25
117	PD-L2	61	0.530	0.694	0.006	310	303	396	329.75
118	Naive CD8$^+$CD28$^+$ cells	53	0.523	0.783	0.069	336	333	328	333.25
119	Non-classical monocytes	53	0.474	0.757	-0.037	329	326	355	334.75
120	IL-6	61	0.502	0.983	0.245	394	395	168	337.75
121	Naive CD8$^+$ cells	53	0.526	0.757	0.016	326	324	379	338.75
122	B-cells	53	0.526	0.757	0.018	328	325	376	339.25
123	CD8$^+$CD57$^+$ cells	53	0.523	0.784	-0.031	335	334	361	341.25
124	EM CD8$^+$CD28$^+$ cells	53	0.480	0.812	0.072	349	345	323	341.5
125	CD4$^+$ cells	53	0.521	0.798	-0.058	345	340	337	341.75
126	EM CD4$^+$ cells	53	0.479	0.796	0.044	343	338	349	343.25
127	CD8$^+$CD28$^+$ cells	53	0.520	0.812	-0.044	348	344	350	347.5

128	TEMRA CD4$^+$CD28$^+$ cells	53	0.484	0.853	0.060	358	358	336	352.5
129	Hematopoietic stem cells	53	0.502	0.986	0.175	395	396	227	353.25
130	miR-19b	61	0.485	0.846	0.029	362	353	364	360.25
131	Intermediate monocytes	53	0.500	1.000	-0.121	402	402	281	371.75
132	Naive Tregs	53	0.487	0.881	0.014	370	367	382	372.25
133	Classical monocytes	53	0.497	0.979	0.017	393	391	378	388.75
134	TEMRA CD8$^+$CD57$^+$ cells	53	0.503	0.979	-0.002	392	390	401	393.75
**LOW CD3 INFILTRATION**									
1	T-cell *P16*^{INK4a}	41	0.755	0.017	-1.754	2	15	8	6.75
2	CM CD8$^+$CD27$^+$CD28$^+$ cells	53	0.254	0.007	0.900	5	6	30	11.5
3	CM CD8$^+$ cells	53	0.248	0.007	0.819	4	4	38	12.5
4	CM CD8$^+$CD28$^+$ cells	53	0.254	0.008	0.855	6	9	36	14.25
5	CM CD8$^+$CD27$^+$ cells	53	0.260	0.010	0.874	7	12	33	14.75
6	PD-1	61	0.689	0.029	-1.757	19	18	7	15.75
7	PD-L1	61	0.688	0.030	-0.868	20	19	34	23.25
8	CM CD8$^+$CD57$^+$ cells	53	0.295	0.028	0.599	15	17	59	26.5
9	EM CD4$^+$CD27$^-$CD28$^-$ cells	53	0.687	0.046	-0.732	23	26	46	29.5
10	TEMRA CD4$^+$CD27$^-$CD28$^-$ cells	53	0.673	0.064	-0.769	28	31	40	31.75
11	Naive CD8$^+$CD57$^+$ cells	53	0.341	0.090	1.076	36	42	21	33.75
12	CRP	61	0.326	0.045	0.577	27	25	63	35.5
13	CD8$^+$ cells	53	0.698	0.033	-0.425	17	20	93	36.75
14	let-7e	61	0.337	0.061	0.635	33	30	54	37.5
15	TEMRA CD4$^+$CD57$^+$ cells	53	0.661	0.086	-0.679	35	40	51	40.25
16	miR-181a	61	0.332	0.053	0.512	31	28	76	41.5
17	Naive CD8$^+$CD27$^-$CD28$^-$ cells	53	0.346	0.100	0.710	39	47	47	43
18	miR-195	61	0.638	0.112	-0.907	49	52	29	44.75
19	miR-92a	61	0.323	0.042	0.378	25	23	107	45
20	CD4$^+$CD27$^-$CD28$^-$ cells	53	0.649	0.111	-0.678	41	51	52	46.25
21	Intermediate monocytes	53	0.335	0.077	0.371	32	35	108	51.75
22	CM CD4$^+$CD28$^+$ cells	53	0.354	0.119	0.555	43	56	66	52
23	Naive B-cells	53	0.329	0.067	0.333	29	32	125	53.75
24	CM CD4$^+$ cells	53	0.358	0.130	0.560	45	61	65	54
25	EM CD4$^+$CD57$^+$ cells	53	0.635	0.151	-0.749	53	69	44	54.75
26	CD4/CD8 ratio	53	0.359	0.131	0.547	46	62	69	55.75
27	CM CD4$^+$CD57$^+$ cells	53	0.369	0.163	0.687	57	71	50	58.75
28	Tregs	53	0.372	0.172	0.463	61	75	85	70.5
29	IL-17A	61	0.608	0.215	-1.934	91	99	6	71.75
30	CM CD4$^+$CD27$^+$ cells	53	0.377	0.192	0.524	65	84	73	71.75
31	miR-19b	61	0.641	0.106	-0.302	48	49	143	72
32	CM CD4$^+$CD27$^+$CD28$^+$ cells	53	0.378	0.193	0.524	66	85	72	72.25
33	MCP-1	61	0.346	0.076	0.185	38	34	216	81.5
34	Tumor grade	61	0.641	0.054	-0.178	47	29	221	86
35	CM CD8$^+$CD27$^-$CD28$^-$ cells	53	0.384	0.215	0.347	74	97	117	90.5
36	Class-switched memory B-cells	53	0.620	0.200	-0.306	68	87	140	90.75
37	EM CD8$^+$CD27$^+$CD28$^+$ cells	53	0.381	0.206	0.275	71	94	153	97.25

38	TEMRA CD8$^+$ cells	53	0.617	0.211	-0.280	73	95	152	98.25
39	Naive CD4$^+$CD57$^+$ cells	53	0.404	0.306	0.671	109	126	53	99.25
40	CD4$^+$ Tregs	53	0.377	0.189	0.208	64	83	196	101.75
41	EM CD8$^+$CD27$^+$ cells	53	0.387	0.227	0.286	79	103	149	102.5
42	sCD27	61	0.399	0.247	0.414	102	109	98	102.75
43	CD4$^+$CD28$^+$ cells	53	0.338	0.084	0.086	34	37	312	104.25
44	Non-switched memory B-cells	53	0.604	0.272	-0.322	97	117	131	110.5
45	miR-9	61	0.579	0.293	-0.915	153	123	28	114.25
46	TEMRA CD8$^+$CD27$^-$CD28$^-$ cells	53	0.594	0.316	-0.317	113	134	134	123.5
47	CD8$^+$CD27$^-$CD28$^-$ cells	53	0.594	0.320	-0.315	112	135	136	123.75
48	IL-6	61	0.388	0.200	0.155	84	89	245	125.5
49	Age	61	0.398	0.241	0.179	100	107	219	131.5
50	CD8$^+$CD27$^+$ cells	53	0.400	0.287	0.196	105	121	209	135
51	miR-17	61	0.410	0.303	0.230	126	125	184	140.25
52	TEMRA CD8$^+$CD27$^+$ cells	53	0.587	0.358	-0.265	135	156	156	145.5
53	TEMRA CD8$^+$CD57$^+$ cells	53	0.587	0.362	-0.262	136	158	158	147
54	TEMRA CD4$^+$ cells	53	0.577	0.419	-0.523	161	193	74	147.25
55	Lymph node involvement	61	0.572	0.343	-0.442	179	147	87	148
56	CD86	61	0.580	0.365	-0.301	152	159	145	152
57	EM CD8$^+$CD27$^-$CD28$^-$ cells	53	0.575	0.426	-0.502	170	198	77	153.75
58	NK-cells	53	0.583	0.384	-0.265	144	172	157	154.25
59	CD4$^+$CD57$^+$ cells	53	0.575	0.431	-0.477	169	199	82	154.75
60	CD8$^+$CD27$^+$CD28$^+$ cells	53	0.413	0.358	0.206	137	157	198	157.25
61	miR-155	61	0.424	0.384	0.301	168	171	144	162.75
62	CD8$^+$CD28$^+$ cells	53	0.412	0.351	0.158	129	151	242	162.75
63	let-7i	61	0.389	0.203	-0.005	85	92	399	165.25
64	Classical monocytes	53	0.602	0.282	-0.049	101	120	346	167
65	LAG-3	61	0.590	0.306	-0.108	125	127	295	168
66	TEMRA CD8$^+$CD27$^+$CD28$^+$ cells	53	0.571	0.451	-0.353	182	206	115	171.25
67	miR-20a	61	0.578	0.370	-0.176	159	161	223	175.5
68	CD4$^+$CD27$^+$CD28$^+$ cells	53	0.415	0.373	0.139	141	163	260	176.25
69	G8 score	26	0.395	0.391	0.051	96	179	342	178.25
70	IL-8	61	0.412	0.311	0.066	130	130	331	180.25
71	CD4$^+$CD27$^+$ cells	53	0.417	0.380	0.136	145	169	264	180.75
72	TEMRA CD8$^+$CD28$^+$ cells	53	0.573	0.438	-0.236	176	204	176	183
73	Myeloid dendritic cells	53	0.563	0.502	-0.426	206	233	92	184.25
74	B-cells	53	0.410	0.341	0.027	122	146	365	188.75
75	CD4$^+$ cells	53	0.425	0.431	0.172	171	200	229	192.75
76	IL-27	61	0.575	0.393	0.150	172	180	249	193.25
77	4-1BB	61	0.546	0.534	-1.457	258	247	15	194.5
78	IL-17F	61	0.464	0.398	1.606	297	185	10	197.25
79	IL-1β	61	0.576	0.383	0.112	167	170	291	198.75
80	TEMRA CD4$^+$CD28$^+$ cells	53	0.562	0.520	-0.313	212	239	138	200.25
81	CM CD4$^+$CD27$^-$CD28$^-$ cells	53	0.545	0.619	1.304	260	265	17	200.5
82	PD-L2	61	0.422	0.374	0.072	158	164	322	200.5

83	Hematopoietic stem cells	53	0.429	0.449	0.159	183	205	240	202.75
84	miR-125b	61	0.565	0.456	-0.176	199	211	224	208.25
85	miR-146a	61	0.443	0.514	0.305	230	236	141	209.25
86	Plasmacytoid dendritic cells	53	0.570	0.457	-0.136	185	212	265	211.75
87	TEMRA $CD4^+CD27^+$ cells	53	0.568	0.469	-0.148	190	223	251	213.5
88	IL-10	61	0.570	0.421	-0.097	184	195	304	216.75
89	Free active TGF-β1	61	0.522	0.431		342	201	1	221.5
90	NK-like T-cells	53	0.542	0.657	-0.402	272	282	103	232.25
91	CTLA-4	61	0.472	0.631	1.138	320	272	19	232.75
92	sCD25	61	0.457	0.625	0.310	269	266	139	235.75
93	TEMRA $CD4^+CD27^+CD28^+$ cells	53	0.560	0.528	-0.125	225	242	279	242.75
94	EM $CD8^+CD28^+$ cells	53	0.442	0.546	0.134	229	251	267	244
95	$CD56^{bright}CD16^-$ NK-cells	53	0.452	0.613	0.168	253	263	235	251
96	EM $CD4^+$ cells	53	0.544	0.642	-0.178	262	278	220	255.5
97	TNF-α	61	0.529	0.743	0.370	316	317	109	264.5
98	Memory Tregs	53	0.448	0.588	0.079	248	261	318	268.75
99	$CD8^+CD57^+$ cells	53	0.540	0.675	-0.133	276	292	268	278
100	$CD56^{dim}CD16^+$ NK-cells	53	0.552	0.588	-0.024	247	260	367	280.25
101	miR-21	61	0.461	0.657	0.130	284	284	274	281.5
102	$CD3^+$ cells	53	0.543	0.649	-0.071	270	279	327	286.5
103	miR-126	61	0.533	0.706	-0.169	305	308	233	287.75
104	EM $CD8^+$ cells	53	0.454	0.631	0.021	255	273	374	289.25
105	EM $CD4^+CD28^+$ cells	53	0.540	0.675	-0.073	277	293	321	292
106	Naive $CD8^+$ cells	53	0.458	0.661	0.037	273	286	354	296.5
107	miR-223	61	0.464	0.688	0.082	295	302	316	302
108	IFN-γ	61	0.534	0.699	0.103	303	305	301	303
109	IL-1α	61	0.470	0.738	0.130	312	316	275	303.75
110	miR-326	61	0.512	0.882	-0.327	371	368	128	309.5
111	TIM-3	61	0.496	0.967	0.489	387	387	80	310.25
112	Gal-9	61	0.486	0.875	0.285	364	364	150	310.5
113	Tumor size	61	0.520	0.826	0.207	350	350	197	311.75
114	IL12p70	61	0.517	0.854	0.188	356	359	213	321
115	Naive $CD4^+CD28^+$ cells	53	0.535	0.718	-0.009	298	309	390	323.75
116	Non-classical monocytes	53	0.471	0.767	0.060	318	329	335	325
117	Naive $CD8^+CD28^+$ cells	53	0.465	0.718	-0.007	299	310	395	325.75
118	EM $CD8^+CD57^+$ cells	53	0.487	0.894	-0.206	369	372	199	327.25
119	Naive $CD4^+CD27^-CD28^-$ cells	53	0.486	0.885	0.175	367	369	226	332.25
120	Naive $CD8^+CD27^+CD28^+$ cells	53	0.475	0.799	-0.071	332	341	325	332.5
121	Naive $CD4^+$ cells	53	0.533	0.736	0.001	307	315	402	332.75
122	Naive $CD4^+CD27^+$ cells	53	0.531	0.752	-0.009	309	321	394	333.25
123	Naive $CD8^+CD27^+$ cells	53	0.478	0.820	-0.071	337	349	326	337.25
124	Naive $CD4^+CD27^+CD28^+$ cells	53	0.529	0.767	-0.013	317	328	387	337.25
125	Monocytes	53	0.515	0.878	0.113	360	365	289	343.5
126	IP-10	61	0.505	0.960	0.133	386	385	269	356.5
127	miR-19a	61	0.518	0.841	0.022	354	352	372	358

128	miR-424	61	0.486	0.874	0.035	365	363	359	363
129	EM CD4$^+$CD27$^+$CD28$^+$ cells	53	0.506	0.959	0.076	382	383	319	366.5
130	miR-18a	61	0.515	0.867	-0.013	361	361	386	367.25
131	EM CD4$^+$CD27$^+$ cells	53	0.506	0.959	0.068	381	382	329	368.25
132	Naive Tregs	53	0.512	0.911	-0.013	372	374	385	375.75
133	IGF-1	61	0.503	0.980	-0.024	390	393	368	385.25
134	miR-150	61	0.499	0.993	-0.013	398	398	384	394.5
