# Supplementary material for: Blood Immunosenescence Signatures Reflecting Age, Frailty and Tumor Immune Infiltrate in Patients with Early Luminal Breast Cancer
Source: Cancers (Basel). 2021 May 2;13(9):2185. doi: 10.3390/cancers13092185 (PMC8125302; doi:10.3390/cancers13092185)
Supplement: Supplementary file 1 [file cancers-13-02185-s001.zip › Table S5 - Individual performance_CD3 infiltration invasive front.pdf]

Table S5: Individual performances of biomarkers correlating with CD3 infiltration in the invasive front (high, intermediate or low infiltration). The table reports the number of patients (N) for which the biomarkers could be measured. The area under the curve (AUC) via receiver operating characteristics (ROC), P-value (Wilcox rank-sum test) and log fold change (FC) were computed for each biomarker. The log FC compared case vs. control. A positive log FC indicates that the measurement is higher than its reference while a negative measurement indicates that is smaller. Based on these statistics AUC, P-value, log FC scores were calculated. The final score combines the 3 scores, where AUC weighted double. The biomarkers are ranked based on their final score.

	Blood markers	N	AUC	*P*-value	log FC	AUC score	*P*-value score	log FC score	Final score
	**HIGH CD3 INFILTRATION**								
1	miR-195	61	0.304	0.024	1.097	17	17	23	18.5
2	miR-150	61	0.311	0.029	0.710	22	21	39	26
3	Tumor grade	61	0.163	0.000	0.399	1	1	103	26.5
4	miR-424	61	0.683	0.036	-0.633	25	28	48	31.5
5	EM $CD4^+CD27^-CD28^-$ cells	53	0.324	0.054	0.563	31	36	60	39.5
6	Gal-9	61	0.643	0.099	-0.661	55	55	44	52.25
7	NK-like T-cells	53	0.358	0.120	0.983	58	69	26	52.75
8	Age	61	0.701	0.021	-0.251	14	13	182	55.75
9	miR-20a	61	0.332	0.053	0.334	36	35	135	60.5
10	IL-1α	61	0.651	0.082	-0.320	42	45	142	67.75
11	miR-125b	61	0.361	0.110	0.374	61	62	113	74.25
12	TEMRA $CD8^+CD57^+$ cells	53	0.357	0.119	0.361	57	66	119	74.75
13	TEMRA $CD8^+CD27^+CD28^+$ cells	53	0.364	0.138	0.388	64	78	110	79
14	Hematopoietic stem cells	53	0.621	0.185	-0.551	86	95	62	82.25
15	miR-126	61	0.376	0.154	0.485	82	85	80	82.25
16	TEMRA $CD8^+CD27^+$ cells	53	0.369	0.152	0.369	72	83	115	85.5
17	G8 score	26	0.746	0.174	-0.145	4	92	264	91
18	TIM-3	61	0.614	0.191	-0.470	95	102	86	94.5
19	IL-17A	61	0.401	0.258	2.255	125	124	6	95
20	TEMRA $CD4^+CD27^-CD28^-$ cells	53	0.357	0.118	-0.189	56	65	231	102
21	TEMRA $CD8^+CD27^-CD28^-$ cells	53	0.380	0.190	0.311	87	100	145	104.75
22	CD86	61	0.607	0.221	-0.344	104	109	130	111.75
23	4-1BB	61	0.418	0.261	1.945	158	126	7	112.25
24	$CD4^+CD27^-CD28^-$ cells	53	0.361	0.128	0.158	60	74	256	112.5
25	Naive $CD8^+CD27^-CD28^-$ cells	53	0.370	0.155	0.180	73	86	239	117.75
26	TEMRA $CD4^+CD57^+$ cells	53	0.373	0.164	-0.183	76	88	233	118.25
27	TEMRA $CD4^+CD27^+$ cells	53	0.396	0.254	0.337	113	120	131	119.25
28	Monocytes	53	0.375	0.175	0.170	81	94	246	125.5
29	TEMRA $CD8^+$ cells	53	0.385	0.207	0.210	93	105	216	126.75
30	TEMRA $CD4^+CD27^+CD28^+$ cells	53	0.401	0.280	0.336	122	137	133	128.5
31	$CD4^+CD28^+$ cells	53	0.648	0.104	-0.029	49	60	369	131.75
32	$CD8^+$ cells	53	0.394	0.249	0.222	108	119	194	132.25
33	miR-223	61	0.404	0.273	0.327	131	134	136	133
34	sCD25	61	0.597	0.269	-0.303	128	131	149	134
35	IFN-γ	61	0.586	0.322	-0.434	150	158	96	138.5

36	let-7i	61	0.604	0.231	-0.213	115	117	207	138.5
37	CM $CD4^+CD27^-CD28^-$ cells	53	0.391	0.213	-0.172	102	107	245	139
38	let-7e	61	0.587	0.319	-0.379	148	155	112	140.75
39	IL-6	61	0.583	0.344	-0.453	155	167	90	141.75
40	$CD8^+CD57^+$ cells	53	0.397	0.266	0.218	119	129	202	142.25
41	EM $CD4^+CD57^+$ cells	53	0.424	0.408	0.476	168	184	84	151
42	EM $CD4^+CD27^+CD28^+$ cells	53	0.408	0.322	0.259	140	157	174	152.75
43	Lymph node involvement	61	0.572	0.343	-0.442	177	166	94	153.5
44	miR-9	61	0.438	0.413	1.451	208	188	17	155.25
45	EM $CD4^+CD27^+$ cells	53	0.410	0.332	0.256	142	162	176	155.5
46	$CD8^+CD27^-CD28^-$ cells	53	0.407	0.312	0.221	138	148	198	155.5
47	Intermediate monocytes	53	0.604	0.258	-0.122	114	123	285	159
48	B-cells	53	0.595	0.303	-0.193	135	145	230	161.25
49	miR-155	61	0.430	0.426	0.421	186	192	99	165.75
50	Naive $CD8^+CD57^+$ cells	53	0.388	0.222	0.036	98	111	359	166.5
51	IL12p70	61	0.564	0.466	-0.554	198	210	61	166.75
52	Class-switched memory B-cells	53	0.415	0.353	0.198	152	172	224	175
53	$CD4^+CD57^+$ cells	53	0.407	0.312	0.125	137	147	284	176.25
54	CRP	61	0.571	0.421	-0.271	180	189	166	178.75
55	MCP-1	61	0.588	0.315	-0.133	146	150	279	180.25
56	IL-27	61	0.564	0.461	-0.317	194	205	144	184.25
57	$CD8^+CD28^+$ cells	53	0.588	0.342	-0.130	145	165	282	184.25
58	IL-17F	61	0.464	0.398	1.595	274	182	13	185.75
59	Tumor size	61	0.575	0.384	-0.182	169	177	234	187.25
60	CD4/CD8 ratio	53	0.563	0.493	-0.336	200	218	132	187.5
61	Naive $CD4^+CD27^-CD28^-$ cells	53	0.397	0.263	0.008	118	127	394	189.25
62	miR-92a	61	0.434	0.451	0.255	190	202	177	189.75
63	Tregs	53	0.571	0.437	-0.208	178	198	217	192.75
64	PD-1	61	0.457	0.627	-0.785	260	264	34	204.5
65	NK-cells	53	0.570	0.454	-0.150	184	203	261	208
66	miR-326	61	0.561	0.412	0.194	209	187	229	208.5
67	Free active TGF-β1	61	0.522	0.431		321	196	3	210.25
68	Naive $CD4^+CD28^+$ cells	53	0.564	0.486	-0.181	196	214	237	210.75
69	TNF-α	61	0.544	0.615	-0.489	253	262	77	211.25
70	T-cell $P16^{INK4a}$	41	0.456	0.680	-0.490	255	279	76	216.25
71	EM $CD4^+$ cells	53	0.441	0.525	0.208	219	228	218	221
72	PD-L1	61	0.449	0.563	0.270	238	244	168	222
73	Memory Tregs	53	0.436	0.491	0.112	197	215	293	225.5
74	IGF-1	61	0.438	0.481	0.097	202	213	300	229.25
75	Naive $CD4^+$ cells	53	0.559	0.529	-0.162	220	229	253	230.5
76	Naive $CD4^+CD27^+CD28^+$ cells	53	0.557	0.543	-0.177	225	237	240	231.75
77	Naive B-cells	53	0.562	0.503	-0.111	205	224	294	232
78	miR-21	61	0.451	0.575	0.221	243	250	197	233.25
79	EM $CD8^+$ cells	53	0.559	0.529	-0.143	221	230	267	234.75
80	Naive $CD4^+CD27^+$ cells	53	0.557	0.543	-0.148	224	236	263	236.75

81	Classical monocytes	53	0.434	0.478	0.012	191	212	388	245.5
82	PD-L2	61	0.555	0.534	-0.104	227	233	298	246.25
83	TEMRA CD4$^+$CD28$^+$ cells	53	0.456	0.639	0.210	254	266	214	247
84	IL-8	61	0.559	0.497	-0.061	217	221	336	247.75
85	TEMRA CD4$^+$ cells	53	0.438	0.503	0.030	204	223	368	249.75
86	Naive Tregs	53	0.559	0.529	-0.046	222	231	350	256.25
87	CM CD8$^+$ cells	53	0.532	0.732	-0.278	284	298	163	257.25
88	sCD27	61	0.538	0.673	-0.205	267	276	220	257.5
89	miR-19b	61	0.451	0.575	0.095	242	249	301	258.5
90	IL-1β	61	0.523	0.795	-0.373	314	316	114	264.5
91	EM CD8$^+$CD28$^+$ cells	53	0.542	0.654	-0.142	262	270	270	266
92	IP-10	61	0.537	0.675	-0.143	271	278	265	271.25
93	CD56^{dim}CD16$^+$ NK-cells	53	0.451	0.597	0.031	241	254	366	275.5
94	EM CD8$^+$CD27$^-$CD28$^-$ cells	53	0.460	0.664	-0.067	263	273	328	281.75
95	CD4$^+$ cells	53	0.537	0.697	-0.083	272	282	312	284.5
96	Non-classical monocytes	53	0.548	0.611	0.006	244	260	396	286
97	CM CD8$^+$CD27$^+$CD28$^+$ cells	53	0.520	0.834	-0.273	325	330	165	286.25
98	Plasmacytoid dendritic cells	53	0.516	0.872	-0.334	338	341	134	287.75
99	CM CD8$^+$CD27$^+$ cells	53	0.518	0.848	-0.293	330	335	156	287.75
100	Myeloid dendritic cells	53	0.472	0.762	0.143	296	308	268	292
101	EM CD4$^+$CD28$^+$ cells	53	0.471	0.757	0.131	292	307	281	293
102	CD56^{bright}CD16$^-$ NK-cells	53	0.466	0.716	0.061	277	290	335	294.75
103	EM CD8$^+$CD27$^+$CD28$^+$ cells	53	0.469	0.742	-0.091	288	303	303	295.5
104	CM CD8$^+$CD57$^+$ cells	53	0.492	0.936	-0.386	363	365	111	300.5
105	LAG-3	61	0.474	0.772	-0.134	307	311	277	300.5
106	Naive CD4$^+$CD57$^+$ cells	53	0.465	0.709	-0.025	275	288	374	303
107	CD8$^+$CD27$^+$ cells	53	0.529	0.755	-0.068	293	306	326	304.5
108	CD4$^+$CD27$^+$CD28$^+$ cells	53	0.535	0.712	-0.021	276	289	380	305.25
109	CTLA-4	61	0.477	0.687	0.076	315	281	319	307.5
110	CD3$^+$ cells	53	0.469	0.739	0.041	289	301	354	308.25
111	CM CD8$^+$CD28$^+$ cells	53	0.512	0.904	-0.257	352	356	175	308.75
112	EM CD8$^+$CD27$^+$ cells	53	0.478	0.817	-0.135	318	323	276	308.75
113	miR-181a	61	0.531	0.725	-0.021	287	293	378	311.25
114	miR-19a	61	0.473	0.763	0.065	304	310	331	312.25
115	IL-10	61	0.514	0.880	-0.199	343	347	223	314
116	Non-switched memory B-cells	53	0.467	0.727	0.001	281	295	400	314.25
117	CM CD4$^+$CD57$^+$ cells	53	0.471	0.754	0.002	291	305	399	321.5
118	CM CD4$^+$CD28$^+$ cells	53	0.520	0.834	-0.082	324	329	314	322.75
119	CM CD8$^+$CD27$^-$CD28$^-$ cells	53	0.500	1.000	-0.431	401	401	97	325
120	miR-18a	61	0.479	0.815	0.058	322	322	338	326
121	CD8$^+$CD27$^+$CD28$^+$ cells	53	0.520	0.832	-0.066	326	328	330	327.5
122	CM CD4$^+$ cells	53	0.516	0.865	-0.084	337	338	310	330.5
123	CD4$^+$CD27$^+$ cells	53	0.524	0.801	-0.008	312	318	393	333.75
124	miR-17	61	0.488	0.900	0.122	354	354	286	337
125	CD4$^+$ Tregs	53	0.518	0.848	0.040	331	334	356	338

126	Naive CD8$^+$ cells	53	0.509	0.929	-0.079	360	362	316	349.5
127	EM CD8$^+$CD57$^+$ cells	53	0.487	0.897	-0.039	348	352	358	351.5
128	Naive CD8$^+$CD28$^+$ cells	53	0.507	0.944	-0.084	369	372	311	355.25
129	Naive CD8$^+$CD27$^+$ cells	53	0.502	0.992	-0.129	392	391	283	364.5
130	TEMRA CD8$^+$CD28$^+$ cells	53	0.505	0.968	-0.067	382	383	329	369
131	Naive CD8$^+$CD27$^+$CD28$^+$ cells	53	0.500	1.000	-0.117	402	402	291	374.25
132	CM CD4$^+$CD27$^+$ cells	53	0.502	0.992	-0.053	391	392	341	378.75
133	miR-146a	61	0.503	0.980	-0.045	389	389	351	379.5
134	CM CD4$^+$CD27$^+$CD28$^+$ cells	53	0.499	1.000	-0.051	394	394	342	381
**INTERMEDIATE CD3 INFILTRATION**									
1	NK-cells	53	0.304	0.014	0.502	16	9	73	28.5
2	CD86	61	0.322	0.016	0.533	29	12	69	34.75
3	T-cell *P16*^{INK4a}	41	0.341	0.084	1.336	38	46	18	35
4	CM CD8$^+$CD27$^+$CD28$^+$ cells	53	0.673	0.031	-0.575	32	22	57	35.75
5	CM CD8$^+$CD28$^+$ cells	53	0.676	0.028	-0.542	30	19	67	36.5
6	CM CD8$^+$CD27$^+$ cells	53	0.669	0.036	-0.532	35	30	70	42.5
7	CM CD8$^+$ cells	53	0.669	0.036	-0.493	34	27	75	42.5
8	Lymph node involvement	61	0.361	0.032	0.815	59	23	32	43.25
9	CM CD4$^+$CD27$^+$CD28$^+$ cells	53	0.650	0.063	-0.482	44	39	82	52.25
10	CM CD4$^+$ cells	53	0.649	0.065	-0.488	46	40	78	52.5
11	miR-92a	61	0.649	0.046	-0.456	45	34	88	53
12	CM CD4$^+$CD27$^+$ cells	53	0.649	0.065	-0.481	47	41	83	54.5
13	Hematopoietic stem cells	53	0.356	0.074	0.540	54	43	68	54.75
14	CM CD4$^+$CD28$^+$ cells	53	0.646	0.070	-0.484	52	42	81	56.75
15	PD-1	61	0.389	0.139	1.564	101	79	14	73.75
16	Naive CD4$^+$CD28$^+$ cells	53	0.364	0.091	0.348	63	49	126	75.25
17	Naive CD4$^+$ cells	53	0.367	0.100	0.323	70	56	140	84
18	sCD27	61	0.627	0.090	-0.324	77	48	139	85.25
19	Naive CD4$^+$CD27$^+$CD28$^+$ cells	53	0.374	0.120	0.349	79	68	124	87.5
20	PD-L1	61	0.382	0.114	0.369	88	64	116	89
21	CRP	61	0.615	0.125	-0.428	94	72	98	89.5
22	Naive CD4$^+$CD27$^+$ cells	53	0.374	0.120	0.322	78	67	141	91
23	miR-150	61	0.607	0.153	-0.526	106	84	71	91.75
24	EM CD8$^+$CD27$^+$CD28$^+$ cells	53	0.639	0.086	-0.217	62	47	203	93.5
25	CM CD4$^+$CD57$^+$ cells	53	0.606	0.187	-0.545	107	97	66	94.25
26	miR-155	61	0.603	0.168	-0.573	116	90	58	95
27	EM CD8$^+$CD27$^+$ cells	53	0.633	0.099	-0.230	69	54	191	95.75
28	Gal-9	61	0.377	0.102	0.271	83	58	167	97.75
29	miR-223	61	0.611	0.137	-0.349	99	76	123	99.25
30	Naive CD8$^+$CD57$^+$ cells	53	0.599	0.222	-0.585	124	112	55	103.75
31	CD4$^+$ Tregs	53	0.606	0.190	-0.361	110	101	120	110.25
32	CTLA-4	61	0.570	0.149	-1.717	183	81	9	114
33	CM CD4$^+$CD27$^-$CD28$^-$ cells	53	0.581	0.297	-1.524	161	140	15	119.25
34	Tumor grade	61	0.613	0.074	-0.148	96	44	262	124.5
35	Naive CD8$^+$CD27$^-$CD28$^-$ cells	53	0.589	0.273	-0.344	143	135	129	137.5

36	TEMRA CD8$^+$CD28$^+$ cells	53	0.418	0.310	0.307	156	146	147	151.25
37	EM CD4$^+$CD27$^-$CD28$^-$ cells	53	0.562	0.444	-1.316	206	200	19	157.75
38	miR-181a	61	0.573	0.330	-0.348	175	160	125	158.75
39	EM CD4$^+$ cells	53	0.581	0.314	-0.260	159	149	173	160
40	Free active TGF-β1	61	0.468	0.168		283	89	1	164
41	miR-424	61	0.425	0.316	0.252	171	151	181	168.5
42	IL-27	61	0.427	0.333	0.254	176	163	179	173.5
43	miR-19a	61	0.575	0.316	-0.211	170	152	211	175.75
44	CM CD8$^+$CD27$^-$CD28$^-$ cells	53	0.597	0.229	-0.062	127	116	334	176
45	CM CD8$^+$CD57$^+$ cells	53	0.599	0.222	-0.022	123	110	376	183
46	miR-17	61	0.569	0.356	-0.227	187	173	192	184.75
47	IL-1β	61	0.442	0.444	0.412	223	201	101	187
48	EM CD4$^+$CD27$^+$ cells	53	0.570	0.391	-0.213	182	180	208	188
49	Age	61	0.416	0.260	0.070	153	125	324	188.75
50	PD-L2	61	0.580	0.291	-0.122	165	138	288	189
51	IFN-γ	61	0.438	0.410	0.288	207	185	158	189.25
52	CD8$^+$ cells	53	0.420	0.326	0.140	163	159	272	189.25
53	Tregs	53	0.563	0.438	-0.286	201	199	160	190.25
54	EM CD4$^+$CD27$^+$CD28$^+$ cells	53	0.566	0.421	-0.214	192	190	206	195
55	IL-1α	61	0.425	0.319	0.102	172	156	299	199.75
56	TEMRA CD4$^+$CD28$^+$ cells	53	0.440	0.463	0.243	212	207	184	203.75
57	Intermediate monocytes	53	0.560	0.463	-0.233	214	208	190	206.5
58	EM CD4$^+$CD57$^+$ cells	53	0.537	0.650	-1.127	269	268	22	207
59	miR-21	61	0.555	0.462	-0.262	226	206	171	207.25
60	EM CD8$^+$ cells	53	0.567	0.411	-0.138	188	186	273	208.75
61	MCP-1	61	0.571	0.345	-0.088	181	168	305	208.75
62	Class-switched memory B-cells	53	0.564	0.428	-0.153	195	193	259	210.5
63	IL12p70	61	0.458	0.573	0.485	261	248	79	212.25
64	LAG-3	61	0.441	0.434	0.197	218	197	226	214.75
65	TEMRA CD8$^+$ cells	53	0.440	0.460	0.168	213	204	248	219.5
66	IL-17A	61	0.528	0.707	-1.305	294	286	20	223.5
67	miR-9	61	0.473	0.675	-1.170	298	277	21	223.5
68	CD4/CD8 ratio	53	0.551	0.533	-0.219	239	232	200	227.5
69	sCD25	61	0.434	0.385	0.049	193	179	345	227.5
70	Memory Tregs	53	0.560	0.463	-0.106	215	209	295	233.5
71	Monocytes	53	0.554	0.507	-0.169	232	225	247	234
72	TNF-α	61	0.467	0.665	0.389	282	274	109	236.75
73	EM CD8$^+$CD57$^+$ cells	53	0.553	0.519	-0.164	235	227	251	237
74	TEMRA CD4$^+$ cells	53	0.469	0.704	0.438	286	284	95	237.75
75	CD4$^+$CD57$^+$ cells	53	0.547	0.566	-0.194	245	245	228	240.75
76	Non-switched memory B-cells	53	0.457	0.602	0.222	258	257	195	242
77	EM CD8$^+$CD27$^-$CD28$^-$ cells	53	0.537	0.650	-0.249	270	269	183	248
78	CD4$^+$CD27$^-$CD28$^-$ cells	53	0.543	0.599	-0.181	259	255	236	252.25
79	let-7i	61	0.462	0.614	0.206	266	261	219	253
80	TEMRA CD4$^+$CD57$^+$ cells	53	0.479	0.803	0.612	323	319	51	254

81	miR-20a	61	0.545	0.549	-0.106	248	239	296	257.75
82	IL-17F	61	0.519	0.606	-0.357	328	258	121	258.75
83	miR-326	61	0.449	0.429	0.031	240	194	367	260.25
84	EM CD8$^+$CD28$^+$ cells	53	0.547	0.566	-0.082	246	246	313	262.75
85	CD8$^+$CD27$^+$ cells	53	0.544	0.587	-0.088	251	252	304	264.5
86	IL-6	61	0.526	0.729	0.293	305	297	155	265.5
87	TEMRA CD8$^+$CD57$^+$ cells	53	0.457	0.602	0.105	257	256	297	266.75
88	TEMRA CD8$^+$CD27$^-$CD28$^-$ cells	53	0.461	0.630	0.133	264	265	278	267.75
89	TIM-3	61	0.474	0.736	-0.266	308	300	170	271.5
90	Naive CD4$^+$CD57$^+$ cells	53	0.527	0.742	-0.222	300	302	196	274.5
91	Plasmacytoid dendritic cells	53	0.469	0.708	0.184	290	287	232	274.75
92	CD4$^+$CD28$^+$ cells	53	0.456	0.587	0.032	250	251	364	278.75
93	miR-146a	61	0.527	0.724	-0.204	303	291	221	279.5
94	Non-classical monocytes	53	0.456	0.590	-0.029	252	253	370	281.75
95	Myeloid dendritic cells	53	0.519	0.817	-0.274	327	324	164	285.5
96	CD8$^+$CD27$^+$CD28$^+$ cells	53	0.531	0.702	-0.087	285	283	306	289.75
97	4-1BB	61	0.504	0.952	-1.022	383	379	25	292.5
98	TEMRA CD4$^+$CD27$^-$CD28$^-$ cells	53	0.494	0.950	0.655	376	376	45	293.25
99	IGF-1	61	0.533	0.662	-0.046	279	272	349	294.75
100	miR-195	61	0.512	0.874	-0.325	351	344	138	296
101	miR-126	61	0.522	0.773	-0.173	317	313	243	297.5
102	IL-10	61	0.488	0.874	0.294	350	343	153	299
103	CD56^{dim}CD16$^+$ NK-cells	53	0.536	0.665	-0.013	273	275	387	302
104	miR-18a	61	0.526	0.729	-0.081	306	296	315	305.75
105	IP-10	61	0.527	0.724	-0.049	302	292	344	310
106	NK-like T-cells	53	0.505	0.957	-0.411	381	380	102	311
107	Naive CD4$^+$CD27$^-$CD28$^-$ cells	53	0.528	0.735	0.025	297	299	373	316.5
108	TEMRA CD4$^+$CD27$^+$ cells	53	0.476	0.776	0.063	313	314	332	318
109	Naive CD8$^+$ cells	53	0.486	0.867	0.162	341	340	254	319
110	miR-19b	61	0.478	0.773	0.058	316	312	337	320.25
111	Naive Tregs	53	0.473	0.744	0.021	301	304	379	321.25
112	Naive CD8$^+$CD27$^+$ cells	53	0.492	0.929	0.224	365	363	193	321.5
113	G8 score	26	0.473	0.831	0.021	299	327	381	326.5
114	Naive CD8$^+$CD28$^+$ cells	53	0.491	0.922	0.182	362	358	235	329.25
115	Tumor size	61	0.488	0.878	-0.140	353	345	271	330.5
116	Naive CD8$^+$CD27$^+$CD28$^+$ cells	53	0.494	0.951	0.214	377	377	205	334
117	TEMRA CD4$^+$CD27$^+$CD28$^+$ cells	53	0.483	0.838	0.049	334	331	346	336.25
118	Classical monocytes	53	0.481	0.825	0.028	329	326	372	339
119	CD4$^+$ cells	53	0.513	0.881	-0.067	344	348	327	340.75
120	EM CD4$^+$CD28$^+$ cells	53	0.513	0.881	-0.062	345	349	333	343
121	CD4$^+$CD27$^+$CD28$^+$ cells	53	0.483	0.839	0.016	333	332	383	345.25
122	TEMRA CD8$^+$CD27$^+$CD28$^+$ cells	53	0.514	0.866	0.036	342	339	361	346
123	CD8$^+$CD57$^+$ cells	53	0.517	0.839	-0.011	335	333	390	348.25
124	IL-8	61	0.494	0.937	0.085	373	368	308	355.5
125	CD56^{bright}CD16$^-$ NK-cells	53	0.493	0.936	0.071	371	367	323	358

126	miR-125b	61	0.494	0.942	-0.073	375	370	321	360.25
127	$CD4^{+}CD27^{+}$ cells	53	0.489	0.901	0.007	355	355	395	365
128	let-7e	61	0.506	0.937	-0.047	374	369	348	366.25
129	$CD3^{+}$ cells	53	0.495	0.957	0.033	380	381	363	376
130	B-cells	53	0.500	1.000	0.077	399	399	318	378.75
131	$CD8^{+}CD28^{+}$ cells	53	0.506	0.951	-0.011	378	378	391	381.25
132	$CD8^{+}CD27^{-}CD28^{-}$ cells	53	0.499	0.993	0.035	393	393	362	385.25
133	TEMRA $CD8^{+}CD27^{+}$ cells	53	0.499	1.000	-0.015	395	395	385	392.5
134	Naive B-cells	53	0.500	1.000	-0.029	400	400	371	392.75
	**LOW CD3 INFILTRATION**								
1	T-cell *P16*^{INK4a}	41	0.807	0.008	-2.267	2	4	5	3.25
2	CM $CD8^{+}CD27^{+}CD28^{+}$ cells	53	0.258	0.007	0.871	5	3	28	10.25
3	CM $CD8^{+}$ cells	53	0.251	0.006	0.793	3	2	33	10.25
4	CM $CD8^{+}CD28^{+}$ cells	53	0.262	0.009	0.826	6	6	31	12.25
5	CM $CD8^{+}CD27^{+}$ cells	53	0.266	0.010	0.843	7	7	29	12.5
6	PD-L1	61	0.710	0.016	-0.930	9	11	27	14
7	CRP	61	0.274	0.008	0.741	8	5	38	14.75
8	PD-1	61	0.692	0.027	-1.767	20	18	8	16.5
9	CM $CD4^{+}$ cells	53	0.293	0.022	0.636	12	15	46	21.25
10	sCD27	61	0.291	0.015	0.578	11	10	56	22
11	CM $CD4^{+}CD28^{+}$ cells	53	0.293	0.022	0.630	13	16	49	22.75
12	TEMRA $CD8^{+}CD57^{+}$ cells	53	0.698	0.029	-0.571	15	20	59	27.25
13	miR-195	61	0.679	0.039	-1.037	28	31	24	27.75
14	CM $CD4^{+}CD27^{+}$ cells	53	0.308	0.034	0.602	18	24	53	28.25
15	CM $CD4^{+}CD27^{+}CD28^{+}$ cells	53	0.309	0.036	0.602	21	29	52	30.75
16	$CD8^{+}$ cells	53	0.709	0.021	-0.443	10	14	93	31.75
17	NK-cells	53	0.681	0.046	-0.548	27	33	64	37.75
18	TEMRA $CD8^{+}$ cells	53	0.692	0.035	-0.469	19	26	87	37.75
19	TEMRA $CD8^{+}CD27^{-}CD28^{-}$ cells	53	0.670	0.062	-0.548	33	38	65	42.25
20	TEMRA $CD4^{+}CD57^{+}$ cells	53	0.654	0.092	-0.684	40	52	41	43.25
21	TEMRA $CD4^{+}CD27^{-}CD28^{-}$ cells	53	0.650	0.100	-0.747	43	57	37	45
22	Intermediate monocytes	53	0.319	0.046	0.392	26	32	106	47.5
23	Tregs	53	0.348	0.096	0.523	41	53	72	51.75
24	$CD4^{+}$ Tregs	53	0.346	0.092	0.392	39	51	107	59
25	Tumor grade	61	0.685	0.012	-0.236	23	8	188	60.5
26	NK-like T-cells	53	0.636	0.138	-0.677	65	77	42	62.25
27	EM $CD8^{+}CD27^{+}$ cells	53	0.352	0.104	0.399	48	59	104	64.75
28	MCP-1	61	0.317	0.035	0.238	24	25	187	65
29	PD-L2	61	0.338	0.061	0.255	37	37	178	72.25
30	Lymph node involvement	61	0.616	0.124	-0.762	92	71	35	72.5
31	EM $CD8^{+}CD27^{+}CD28^{+}$ cells	53	0.353	0.109	0.347	51	61	127	72.5
32	miR-125b	61	0.647	0.091	-0.317	50	50	143	73.25
33	TEMRA $CD4^{+}CD27^{+}$ cells	53	0.635	0.141	-0.473	67	80	85	74.75
34	CD4/CD8 ratio	53	0.372	0.161	0.550	75	87	63	75
35	CD86	61	0.633	0.126	-0.416	68	73	100	77.25

36	miR-181a	61	0.370	0.136	0.454	74	75	89	78
37	EM $CD8^{+}$ cells	53	0.355	0.114	0.301	53	63	151	80
38	TEMRA $CD4^{+}CD28^{+}$ cells	53	0.621	0.189	-0.587	85	99	54	80.75
39	TEMRA $CD8^{+}CD27^{+}$ cells	53	0.632	0.149	-0.399	71	82	105	82.25
40	miR-92a	61	0.365	0.121	0.326	66	70	137	84.75
41	CM $CD8^{+}CD27^{-}CD28^{-}$ cells	53	0.375	0.173	0.448	80	91	92	85.75
42	TEMRA $CD4^{+}CD27^{+}CD28^{+}$ cells	53	0.621	0.186	-0.450	84	96	91	88.75
43	TEMRA $CD8^{+}CD27^{+}CD28^{+}$ cells	53	0.617	0.200	-0.501	91	104	74	90
44	CM $CD4^{+}CD57^{+}$ cells	53	0.393	0.242	0.623	105	118	50	94.5
45	CM $CD8^{+}CD57^{+}$ cells	53	0.382	0.197	0.369	89	103	117	99.5
46	TEMRA $CD4^{+}$ cells	53	0.603	0.266	-0.668	120	130	43	103.25
47	EM $CD4^{+}CD27^{-}CD28^{-}$ cells	53	0.596	0.294	0.829	129	139	30	106.75
48	CTLA-4	61	0.429	0.208	1.646	179	106	10	118.5
49	Naive $CD4^{+}CD28^{+}$ cells	53	0.611	0.226	-0.285	100	114	161	118.75
50	Naive $CD4^{+}$ cells	53	0.612	0.225	-0.269	97	113	169	119
51	4-1BB	61	0.576	0.297	-1.459	167	141	16	122.75
52	TEMRA $CD8^{+}CD28^{+}$ cells	53	0.601	0.272	-0.350	121	132	122	124
53	let-7e	61	0.404	0.273	0.390	130	133	108	125.25
54	Naive $CD4^{+}CD27^{+}CD28^{+}$ cells	53	0.604	0.258	-0.291	112	122	157	125.75
55	Naive $CD4^{+}CD27^{+}$ cells	53	0.604	0.258	-0.282	111	121	162	126.25
56	miR-20a	61	0.607	0.218	-0.219	103	108	201	128.75
57	miR-9	61	0.599	0.188	-0.261	126	98	172	130.5
58	TIM-3	61	0.420	0.365	0.706	164	175	40	135.75
59	EM $CD8^{+}CD28^{+}$ cells	53	0.397	0.266	0.235	117	128	189	137.75
60	miR-126	61	0.594	0.280	-0.309	136	136	146	138.5
61	$CD8^{+}CD27^{-}CD28^{-}$ cells	53	0.595	0.303	-0.286	132	142	159	141.25
62	Non-switched memory B-cells	53	0.588	0.342	-0.301	144	164	150	150.5
63	LAG-3	61	0.606	0.227	-0.138	109	115	274	151.75
64	IL-6	61	0.382	0.175	0.023	90	93	375	162
65	IL-17A	61	0.560	0.492	-1.615	211	217	11	162.5
66	$CD8^{+}CD28^{+}$ cells	53	0.405	0.303	0.138	133	143	275	171
67	miR-424	61	0.419	0.353	0.215	160	171	204	173.75
68	$CD8^{+}CD57^{+}$ cells	53	0.581	0.384	-0.220	162	178	199	175.25
69	$CD8^{+}CD27^{+}$ cells	53	0.414	0.348	0.175	151	169	241	178
70	miR-155	61	0.430	0.426	0.298	185	191	152	178.25
71	Age	61	0.413	0.319	0.142	147	154	269	179.25
72	B-cells	53	0.405	0.303	0.087	134	144	307	179.75
73	miR-17	61	0.418	0.348	0.175	157	170	242	181.5
74	miR-19a	61	0.425	0.393	0.211	173	181	212	184.75
75	miR-19b	61	0.579	0.366	-0.180	166	176	238	186.5
76	IP-10	61	0.427	0.402	0.199	174	183	222	188.25
77	Tumor size	61	0.441	0.494	0.345	216	219	128	194.75
78	Classical monocytes	53	0.590	0.332	-0.048	141	161	347	197.5
79	$CD4^{+}CD27^{-}CD28^{-}$ cells	53	0.584	0.359	0.068	154	174	325	201.75
80	$CD4^{+}CD28^{+}$ cells	53	0.408	0.318	-0.012	139	153	389	205

81	Free active TGF-β1	61	0.522	0.431		319	195	2	208.75
82	Hematopoietic stem cells	53	0.563	0.491	-0.197	199	216	225	209.75
83	G8 score	26	0.413	0.494	0.043	149	220	352	217.5
84	EM CD8$^+$CD57$^+$ cells	53	0.445	0.556	0.240	228	240	186	220.5
85	CD8$^+$CD27$^+$CD28$^+$ cells	53	0.440	0.512	0.173	210	226	244	222.5
86	Naive B-cells	53	0.438	0.503	0.143	203	222	266	223.5
87	IL-1β	61	0.554	0.535	-0.211	230	234	213	226.75
88	EM CD4$^+$CD57$^+$ cells	53	0.528	0.762	0.752	295	309	36	233.75
89	Naive CD4$^+$CD27$^-$CD28$^-$ cells	53	0.567	0.467	-0.040	189	211	357	236.5
90	miR-146a	61	0.461	0.657	0.304	265	271	148	237.25
91	CD4$^+$ cells	53	0.447	0.569	0.163	233	247	252	241.25
92	IL-1α	61	0.449	0.563	0.160	236	243	255	242.5
93	miR-223	61	0.446	0.535	0.122	231	235	287	246
94	let-7i	61	0.447	0.547	-0.073	234	238	322	257
95	CD56^{bright}CD16$^-$ NK-cells	53	0.543	0.643	-0.157	256	267	257	259
96	IL-17F	61	0.510	0.819	-2.426	356	325	4	260.25
97	EM CD4$^+$ cells	53	0.454	0.621	0.115	247	263	292	262.25
98	Monocytes	53	0.555	0.556	0.041	229	241	355	263.5
99	IL-8	61	0.449	0.563	-0.055	237	242	339	263.75
100	miR-150	61	0.545	0.609	-0.091	249	259	302	264.75
101	Naive CD8$^+$CD57$^+$ cells	53	0.485	0.880	0.634	339	346	47	267.75
102	CD3$^+$ cells	53	0.538	0.687	-0.085	268	280	309	281.25
103	CD4$^+$CD57$^+$ cells	53	0.533	0.727	0.119	280	294	290	286
104	CM CD4$^+$CD27$^-$CD28$^-$ cells	53	0.505	0.958	1.602	379	382	12	288
105	Gal-9	61	0.522	0.810	0.212	320	321	210	292.75
106	Naive CD8$^+$CD27$^-$CD28$^-$ cells	53	0.516	0.864	0.242	336	337	185	298.5
107	IL-27	61	0.533	0.706	-0.042	278	285	353	298.5
108	miR-21	61	0.475	0.776	0.120	309	315	289	305.5
109	EM CD8$^+$CD27$^-$CD28$^-$ cells	53	0.493	0.944	0.362	367	371	118	305.75
110	Plasmacytoid dendritic cells	53	0.524	0.801	0.078	311	317	317	314
111	miR-326	61	0.507	0.929	-0.254	370	364	180	321
112	sCD25	61	0.491	0.927	0.213	361	359	209	322.5
113	Naive Tregs	53	0.476	0.803	0.019	310	320	382	330.5
114	Naive CD8$^+$CD27$^+$ cells	53	0.508	0.936	-0.167	364	366	249	335.75
115	Naive CD4$^+$CD57$^+$ cells	53	0.500	1.000	0.293	397	397	154	336.25
116	Naive CD8$^+$ cells	53	0.509	0.929	-0.132	358	360	280	339
117	Naive CD8$^+$CD27$^+$CD28$^+$ cells	53	0.507	0.944	-0.165	368	374	250	340
118	IGF-1	61	0.517	0.849	-0.036	332	336	360	340
119	miR-18a	61	0.486	0.874	0.050	340	342	343	341.25
120	IL-10	61	0.503	0.980	-0.210	387	388	215	344.25
121	IL12p70	61	0.493	0.947	-0.151	372	375	260	344.75
122	Myeloid dendritic cells	53	0.504	0.976	0.197	386	384	227	345.75
123	EM CD4$^+$CD28$^+$ cells	53	0.513	0.897	-0.055	349	353	340	347.75
124	Naive CD8$^+$CD28$^+$ cells	53	0.504	0.976	-0.155	384	385	258	352.75
125	Memory Tregs	53	0.487	0.897	0.021	347	351	377	355.5

126	$CD4^+CD27^+CD28^+$ cells	53	0.487	0.897	0.000	346	350	401	360.75
127	Non-classical monocytes	53	0.509	0.929	0.032	359	361	365	361
128	$CD4^+CD27^+$ cells	53	0.490	0.920	0.000	357	357	402	368.25
129	EM $CD4^+CD27^+CD28^+$ cells	53	0.507	0.944	0.002	366	373	398	375.75
130	TNF-α	61	0.500	1.000	-0.074	398	398	320	378.5
131	$CD56^{dim}CD16^+$ NK-cells	53	0.504	0.976	-0.016	385	386	384	385
132	IFN-γ	61	0.497	0.980	0.014	388	387	386	387.25
133	Class-switched memory B-cells	53	0.503	0.984	-0.009	390	390	392	390.5
134	EM $CD4^+CD27^+$ cells	53	0.500	1.000	0.005	396	396	397	396.25
