# Supplementary material for: Blood Immunosenescence Signatures Reflecting Age, Frailty and Tumor Immune Infiltrate in Patients with Early Luminal Breast Cancer
Source: Cancers (Basel). 2021 May 2;13(9):2185. doi: 10.3390/cancers13092185 (PMC8125302; doi:10.3390/cancers13092185)
Supplement: Supplementary file 1 [file cancers-13-02185-s001.zip › Table S6 - Individual performance_CD8 infiltration whole tumor.pdf]

Table S6: Individual performances of biomarkers correlating with CD8 infiltration in the whole tumor (high, intermediate or low infiltration). The table reports the number of patients (N) for which the biomarkers could be measured. The area under the curve (AUC) via receiver operating characteristics (ROC), P-value (Wilcox rank-sum test) and log fold change (FC) are reported for each biomarker. The log FC compared case vs. control. A positive log FC indicates that the measurement is higher than its reference while a negative measurement indicates that is smaller. Based on these statistics AUC, P-value, log FC scores were computed. The final score combines the 3 scores, where AUC weighted double. The biomarkers are ranked based on their final score.

	Blood markers	N	AUC	*P*-value	log FC	AUC score	*P*-value score	log FC score	Final score
					**HIGH CD8 INFILTRATION**				
1	TEMRA CD8$^+$CD27$^+$CD28$^+$ cells	54	0.268	0.009	0.646	10	14	59	23.25
2	TEMRA CD8$^+$CD27$^+$ cells	54	0.281	0.014	0.565	17	19	69	30.5
3	Tumor grade	62	0.200	0.000	0.357	2	1	126	32.75
4	TEMRA CD4$^+$CD27$^+$ cells	54	0.306	0.029	0.767	29	31	47	34
5	IL-1α	62	0.723	0.009	-0.455	16	13	91	34
6	miR-195	62	0.319	0.033	1.000	39	33	32	35.75
7	TEMRA CD4$^+$CD27$^+$CD28$^+$ cells	54	0.315	0.037	0.771	35	37	46	38.25
8	Age	62	0.728	0.007	-0.296	13	11	163	50
9	Gal-9	62	0.652	0.073	-0.658	60	55	55	57.5
10	TEMRA CD4$^+$CD28$^+$ cells	54	0.356	0.105	0.776	65	77	45	63
11	TIM-3	62	0.648	0.081	-0.537	63	61	71	64.5
12	miR-150	62	0.358	0.094	0.610	67	70	61	66.25
13	miR-424	62	0.643	0.093	-0.474	66	69	83	71
14	miR-125b	62	0.369	0.122	0.357	77	85	127	91.5
15	sCD25	62	0.626	0.138	-0.381	87	93	114	95.25
16	B-cells	54	0.634	0.133	-0.276	74	90	176	103.5
17	EM CD4$^+$CD27$^-$CD28$^-$ cells	54	0.391	0.220	0.375	113	130	119	118.75
18	4-1BB	62	0.417	0.241	2.115	172	140	5	122.25
19	Class-switched memory B-cells	54	0.384	0.192	0.257	104	119	186	128.25
20	Monocytes	54	0.374	0.160	0.164	89	99	260	134.25
21	IFN-γ	62	0.591	0.284	-0.486	154	160	80	137
22	IP-10	62	0.607	0.210	-0.243	118	129	199	141
23	CD86	62	0.598	0.253	-0.324	138	149	142	141.75
24	Intermediate monocytes	54	0.619	0.185	-0.160	100	114	265	144.75
25	TEMRA CD8$^+$CD28$^+$ cells	54	0.396	0.243	0.252	123	144	190	145
26	IL-6	62	0.586	0.315	-0.460	167	171	87	148
27	TEMRA CD4$^+$ cells	54	0.402	0.274	0.258	137	156	184	153.5
28	miR-20a	62	0.400	0.240	0.232	134	139	211	154.5
29	TEMRA CD4$^+$CD27$^-$CD28$^-$ cells	54	0.417	0.354	-0.426	171	186	100	157
30	TNF-α	62	0.573	0.393	-0.652	191	197	57	159
31	EM CD4$^+$CD27$^+$CD28$^+$ cells	54	0.403	0.283	0.243	141	159	198	159.75
32	miR-223	62	0.412	0.299	0.282	159	166	173	164.25
33	IL-27	62	0.579	0.355	-0.355	179	187	128	168.25
34	EM CD4$^+$CD27$^+$ cells	54	0.409	0.310	0.240	153	170	202	169.5
35	miR-326	62	0.579	0.275	-0.257	178	157	187	175
36	IL12p70	62	0.565	0.449	-0.606	210	219	63	175.5
37	TEMRA CD4$^+$CD57$^+$ cells	54	0.433	0.457	-0.474	203	223	82	177.75

38	Naive Tregs	54	0.602	0.258	-0.085	130	153	319	183
39	Naive B-cells	54	0.590	0.319	-0.165	155	173	259	185.5
40	CM CD8^{+} cells	54	0.568	0.451	-0.371	202	221	120	186.25
41	CD8^{+} cells	54	0.412	0.328	0.156	162	175	266	191.25
42	TEMRA CD8^{+} cells	54	0.414	0.334	0.154	164	178	267	193.25
43	Naive CD4^{+}CD27^{-}CD28^{-} cells	54	0.394	0.235	-0.001	119	137	400	193.75
44	miR-126	62	0.437	0.459	0.349	218	224	131	197.75
45	miR-9	62	0.453	0.522	1.358	265	251	22	200.75
46	CM CD4^{+}CD28^{+} cells	54	0.573	0.421	-0.226	194	206	216	202.5
47	MCP-1	62	0.583	0.330	-0.128	174	176	289	203.25
48	Memory Tregs	54	0.421	0.378	0.141	177	193	278	206.25
49	CM CD4^{+} cells	54	0.569	0.444	-0.227	200	216	214	207.5
50	Naive CD4^{+}CD27^{+} cells	54	0.571	0.433	-0.197	197	208	238	210
51	miR-19a	62	0.426	0.385	0.154	190	195	269	211
52	Naive CD4^{+}CD28^{+} cells	54	0.569	0.440	-0.192	201	211	241	213.5
53	IL-17F	62	0.469	0.443	1.502	314	214	14	214
54	CM CD4^{+}CD27^{-}CD28^{-} cells	54	0.439	0.481	-0.296	229	240	164	215.5
55	NK-like T-cells	54	0.450	0.582	0.462	257	269	85	217
56	CM CD8^{+}CD28^{+} cells	54	0.556	0.530	-0.354	243	257	130	218.25
57	IL-17A	62	0.460	0.641	2.086	287	294	6	218.5
58	PD-1	62	0.457	0.612	-0.844	275	285	39	218.5
59	miR-92a	62	0.438	0.469	0.240	224	230	200	219.5
60	Free active TGF-β1	62	0.522	0.415		341	203	2	221.75
61	EM CD4^{+} cells	54	0.436	0.475	0.198	212	233	233	222.5
62	CD4^{+}CD28^{+} cells	54	0.588	0.325	0.001	161	174	401	224.25
63	CM CD8^{+}CD27^{+}CD28^{+} cells	54	0.550	0.579	-0.358	254	267	125	225
64	Naive CD4^{+}CD27^{+}CD28^{+} cells	54	0.564	0.479	-0.198	214	238	237	225.75
65	CD4^{+}CD27^{-}CD28^{-} cells	54	0.422	0.385	-0.042	180	194	358	228
66	Naive CD4^{+} cells	54	0.564	0.479	-0.174	213	237	255	229.5
67	EM CD4^{+}CD57^{+} cells	54	0.454	0.609	0.404	268	282	104	230.5
68	Classical monocytes	54	0.422	0.389	0.018	181	196	381	234.75
69	CM CD8^{+}CD27^{+} cells	54	0.545	0.616	-0.376	271	287	117	236.5
70	CM CD4^{+}CD27^{+} cells	54	0.557	0.528	-0.198	237	252	235	240.25
71	IL-8	62	0.565	0.449	-0.075	211	220	325	241.75
72	miR-17	62	0.443	0.504	0.181	241	246	248	244
73	EM CD4^{+}CD28^{+} cells	54	0.443	0.528	0.163	238	253	262	247.75
74	CM CD4^{+}CD27^{+}CD28^{+} cells	54	0.554	0.549	-0.196	247	260	240	248.5
75	CD56^{dim}CD16^{+} NK-cells	54	0.434	0.467	0.038	207	228	362	251
76	Naive CD8^{+}CD57^{+} cells	54	0.438	0.487	-0.085	223	242	318	251.5
77	TEMRA CD8^{+}CD57^{+} cells	54	0.443	0.528	0.137	239	254	281	253.25
78	Tregs	54	0.559	0.511	-0.111	233	249	298	253.25
79	miR-146a	62	0.437	0.459	0.047	219	225	351	253.5
80	Myeloid dendritic cells	54	0.449	0.569	0.143	252	265	276	261.25
81	EM CD8^{+}CD27^{-}CD28^{-} cells	54	0.436	0.475	-0.007	215	234	390	263.5
82	CD3^{+} cells	54	0.440	0.505	0.053	231	247	349	264.5

83	IL-1β	62	0.529	0.741	-0.439	322	329	96	267.25
84	miR-18a	62	0.453	0.584	0.139	264	271	280	269.75
85	Hematopoietic stem cells	54	0.533	0.713	-0.319	308	318	146	270
86	miR-155	62	0.466	0.693	0.273	304	310	180	274.5
87	CRP	62	0.546	0.593	-0.128	266	276	290	274.5
88	PD-L2	62	0.546	0.593	-0.102	267	277	305	279
89	Tumor size	62	0.541	0.626	-0.147	283	291	270	281.75
90	EM CD8$^+$CD57$^+$ cells	54	0.451	0.592	0.046	259	275	353	286.5
91	Non-classical monocytes	54	0.552	0.566	-0.015	250	264	386	287.5
92	CD4$^+$CD57$^+$ cells	54	0.451	0.592	-0.028	258	274	374	291
93	sCD27	62	0.457	0.616	-0.065	276	288	341	295.25
94	TEMRA CD8$^+$CD27$^-$CD28$^-$ cells	54	0.461	0.664	0.096	289	302	307	296.75
95	CD4$^+$ Tregs	54	0.545	0.616	0.029	272	286	372	300.5
96	CD8$^+$CD57$^+$ cells	54	0.462	0.674	0.088	291	306	316	301
97	IL-10	62	0.520	0.815	-0.276	345	347	175	303
98	miR-21	62	0.537	0.670	0.089	298	303	314	303.25
99	CM CD8$^+$CD27$^-$CD28$^-$ cells	54	0.511	0.908	-0.476	378	378	81	303.75
100	CD4$^+$CD27$^+$ cells	54	0.542	0.643	-0.034	278	295	366	304.25
101	CD4$^+$CD27$^+$CD28$^+$ cells	54	0.542	0.646	-0.033	279	296	368	305.5
102	IGF-1	62	0.462	0.661	0.061	293	300	348	308.5
103	CM CD8$^+$CD57$^+$ cells	54	0.511	0.908	-0.431	379	379	99	309
104	CD4/CD8 ratio	54	0.526	0.779	-0.183	330	338	245	310.75
105	T-cell *P16*^{INK4a}	42	0.500	1.000	-0.704	402	402	49	313.75
106	let-7i	62	0.533	0.705	-0.083	310	315	322	314.25
107	Naive CD8$^+$CD27$^-$CD28$^-$ cells	54	0.467	0.714	-0.061	307	319	345	319.5
108	miR-19b	62	0.470	0.729	0.073	319	325	328	322.75
109	PD-L1	62	0.482	0.841	0.198	353	354	236	324
110	Plasmacytoid dendritic cells	54	0.479	0.817	-0.163	343	349	261	324
111	Naive CD8$^+$ cells	54	0.525	0.789	-0.124	334	339	295	325.5
112	CD8$^+$CD27$^-$CD28$^-$ cells	54	0.472	0.760	0.065	323	332	340	329.5
113	Naive CD8$^+$CD27$^+$ cells	54	0.515	0.877	-0.179	361	363	251	334
114	EM CD8$^+$CD27$^+$CD28$^+$ cells	54	0.482	0.847	-0.127	351	355	291	337
115	Naive CD4$^+$CD57$^+$ cells	54	0.476	0.794	-0.069	336	341	337	337.5
116	let-7e	62	0.514	0.879	-0.144	364	365	274	341.75
117	EM CD8$^+$CD27$^+$ cells	54	0.486	0.885	-0.140	363	369	279	343.5
118	CD8$^+$CD28$^+$ cells	54	0.523	0.804	-0.039	339	345	360	345.75
119	miR-181a	62	0.523	0.791	-0.020	338	340	380	349
120	Naive CD8$^+$CD27$^+$CD28$^+$ cells	54	0.509	0.924	-0.168	381	383	258	350.75
121	Naive CD8$^+$CD28$^+$ cells	54	0.513	0.892	-0.117	368	373	296	351.25
122	LAG-3	62	0.503	0.981	-0.208	396	397	224	353.25
123	CD8$^+$CD27$^+$CD28$^+$ cells	54	0.515	0.877	-0.071	360	362	332	353.5
124	CD8$^+$CD27$^+$ cells	54	0.515	0.870	-0.062	358	358	344	354.5
125	CTLA-4	62	0.483	0.763	-0.017	355	334	384	357
126	NK-cells	54	0.518	0.849	-0.022	350	356	378	358.5
127	Non-switched memory B-cells	54	0.485	0.879	-0.043	359	366	357	360.25

128	G8 score	27	0.479	0.937	-0.028	344	387	373	362
129	CD56^{bright}CD16$^-$ NK-cells	54	0.512	0.900	-0.063	373	377	343	366.5
130	Lymph node involvement	62	0.511	0.889	-0.061	380	370	346	369
131	CM CD4$^+$CD57$^+$ cells	54	0.497	0.977	-0.040	394	395	359	385.5
132	CD4$^+$ cells	54	0.491	0.924	0.004	382	384	396	386
133	EM CD8$^+$ cells	54	0.504	0.970	-0.027	392	392	375	387.75
134	EM CD8$^+$CD28$^+$ cells	54	0.494	0.954	-0.016	388	390	385	387.75
	**INTERMEDIATE CD8 INFILTRATION**								
1	CD4$^+$ cells	54	0.769	0.001	-0.607	4	3	62	18.25
2	CD4/CD8 ratio	54	0.701	0.012	-0.682	24	18	51	29.25
3	Memory Tregs	54	0.724	0.005	-0.369	15	7	121	39.5
4	EM CD4$^+$CD57$^+$ cells	54	0.338	0.043	0.510	48	39	75	52.5
5	Tregs	54	0.657	0.050	-0.596	54	42	66	54
6	TEMRA CD8$^+$CD27$^+$CD28$^+$ cells	54	0.669	0.035	-0.409	43	35	103	56
7	CM CD8$^+$CD28$^+$ cells	54	0.651	0.060	-0.559	62	45	70	59.75
8	TEMRA CD4$^+$CD27$^+$CD28$^+$ cells	54	0.640	0.080	-0.696	69	60	50	62
9	CD4$^+$CD57$^+$ cells	54	0.368	0.100	0.863	76	75	38	66.25
10	TEMRA CD4$^+$CD27$^+$ cells	54	0.637	0.088	-0.658	72	67	54	66.25
11	T-cell *P16^{INK4a}*	42	0.372	0.159	1.329	83	98	23	71.75
12	CM CD8$^+$ cells	54	0.633	0.096	-0.492	75	72	78	75
13	Naive Tregs	54	0.290	0.008	0.168	20	12	257	77.25
14	CD3$^+$ cells	54	0.685	0.021	-0.202	37	24	229	81.75
15	CM CD8$^+$CD27$^+$ cells	54	0.628	0.111	-0.420	85	79	102	87.75
16	CM CD8$^+$CD27$^+$CD28$^+$ cells	54	0.625	0.120	-0.458	91	83	88	88.25
17	CD86	62	0.374	0.090	0.387	88	68	109	88.25
18	CD8$^+$CD27$^-$CD28$^-$ cells	54	0.372	0.112	0.382	84	80	113	90.25
19	miR-19a	62	0.629	0.083	-0.319	82	63	145	93
20	CD8$^+$CD28$^+$ cells	54	0.644	0.071	-0.250	64	54	191	93.25
21	CD4$^+$CD27$^-$CD28$^-$ cells	54	0.392	0.180	0.914	115	112	34	94
22	TEMRA CD4$^+$CD57$^+$ cells	54	0.395	0.192	1.292	122	117	26	96.75
23	CD8$^+$CD57$^+$ cells	54	0.374	0.116	0.317	86	82	148	100.5
24	EM CD4$^+$CD27$^-$CD28$^-$ cells	54	0.388	0.161	0.458	108	101	89	101.5
25	Naive CD8$^+$CD28$^+$ cells	54	0.622	0.128	-0.339	94	88	134	102.5
26	sCD27	62	0.623	0.098	-0.314	93	73	152	102.75
27	TEMRA CD4$^+$CD28$^+$ cells	54	0.607	0.185	-0.528	116	113	72	104.25
28	Naive CD8$^+$CD27$^+$CD28$^+$ cells	54	0.624	0.124	-0.311	92	87	155	106.5
29	TEMRA CD8$^+$CD27$^+$ cells	54	0.620	0.134	-0.321	99	91	144	108.25
30	Hematopoietic stem cells	54	0.391	0.173	0.421	114	106	101	108.75
31	Naive CD8$^+$CD27$^+$ cells	54	0.620	0.134	-0.298	98	92	161	112.25
32	MCP-1	62	0.635	0.069	-0.174	73	51	254	112.75
33	CD8$^+$CD27$^+$CD28$^+$ cells	54	0.622	0.130	-0.274	95	89	179	114.5
34	4-1BB	62	0.585	0.169	-1.475	169	104	18	115
35	Gal-9	62	0.382	0.113	0.270	101	81	182	116.25
36	Naive CD4$^+$CD57$^+$ cells	54	0.404	0.233	0.680	142	133	52	117.25
37	G8 score	27	0.686	0.098	-0.069	34	74	335	119.25

38	IFN-γ	62	0.403	0.189	0.391	139	116	107	125.25
39	TEMRA CD4$^+$CD27$^-$CD28$^-$ cells	54	0.414	0.284	1.300	166	161	25	129.5
40	TEMRA CD8$^+$CD27$^-$CD28$^-$ cells	54	0.404	0.233	0.382	143	134	111	132.75
41	CD8$^+$CD27$^+$ cells	54	0.612	0.164	-0.226	110	102	215	134.25
42	Naive CD8$^+$ cells	54	0.604	0.196	-0.287	126	121	169	135.5
43	TEMRA CD8$^+$CD57$^+$ cells	54	0.401	0.222	0.316	135	131	149	137.5
44	Tumor grade	62	0.610	0.081	-0.145	112	62	273	139.75
45	Lymph node involvement	62	0.419	0.207	0.456	176	128	90	142.5
46	CTLA-4	62	0.561	0.202	-1.576	228	123	10	147.25
47	NK-cells	54	0.404	0.235	0.275	144	138	178	151
48	miR-92a	62	0.595	0.202	-0.254	147	124	189	151.75
49	CD4$^+$CD28$^+$ cells	54	0.611	0.166	-0.136	111	103	284	152.25
50	CD4$^+$CD27$^+$CD28$^+$ cells	54	0.604	0.196	-0.173	125	120	256	156.5
51	IL-17F	62	0.548	0.175	-7.396	261	108	4	158.5
52	Monocytes	54	0.417	0.303	0.341	170	168	133	160.25
53	IL-1α	62	0.399	0.176	0.135	132	109	285	164.5
54	CD4$^+$CD27$^+$ cells	54	0.597	0.226	-0.146	140	132	271	170.75
55	miR-326	62	0.435	0.307	0.431	209	169	98	171.25
56	Free active TGF-β1	62	0.467	0.147		306	95	1	177
57	miR-126	62	0.405	0.202	0.132	148	125	287	177
58	CD56^{bright}CD16$^-$ NK-cells	54	0.575	0.352	-0.296	188	184	165	181.25
59	EM CD8$^+$CD28$^+$ cells	54	0.592	0.257	-0.136	152	151	283	184.5
60	miR-9	62	0.548	0.449	-1.481	260	218	16	188.5
61	TNF-α	62	0.434	0.374	0.301	205	192	159	190.25
62	TEMRA CD8$^+$ cells	54	0.424	0.347	0.217	185	183	219	193
63	Age	62	0.411	0.234	0.071	158	135	333	196
64	NK-like T-cells	54	0.428	0.370	0.235	195	189	207	196.5
65	TEMRA CD8$^+$CD28$^+$ cells	54	0.586	0.284	-0.125	165	162	294	196.5
66	Naive B-cells	54	0.576	0.346	-0.179	183	182	250	199.5
67	miR-150	62	0.556	0.455	-0.355	244	222	129	209.75
68	EM CD8$^+$CD27$^-$CD28$^-$ cells	54	0.446	0.503	0.391	246	245	108	211.25
69	Plasmacytoid dendritic cells	54	0.442	0.470	0.284	235	232	172	218.5
70	LAG-3	62	0.439	0.413	0.208	226	202	223	219.25
71	B-cells	54	0.438	0.442	0.202	220	213	230	220.75
72	EM CD8$^+$CD27$^+$CD28$^+$ cells	54	0.575	0.352	-0.067	187	185	338	224.25
73	CD8$^+$ cells	54	0.440	0.463	0.205	232	226	227	229.25
74	Non-classical monocytes	54	0.556	0.495	-0.245	245	244	197	232.75
75	IL-1β	62	0.453	0.530	0.307	263	258	158	235.5
76	EM CD4$^+$CD27$^+$CD28$^+$ cells	54	0.551	0.528	-0.213	251	256	221	244.75
77	IL-17A	62	0.472	0.714	-1.075	327	320	29	250.75
78	IP-10	62	0.442	0.438	0.066	236	209	339	255
79	PD-1	62	0.478	0.767	1.499	340	335	15	257.5
80	IL-8	62	0.557	0.447	-0.072	242	217	330	257.75
81	EM CD4$^+$CD27$^+$ cells	54	0.546	0.575	-0.198	269	266	234	259.5
82	Naive CD4$^+$CD27$^+$CD28$^+$ cells	54	0.550	0.540	-0.154	256	259	268	259.75

83	PD-L1	62	0.463	0.622	0.285	296	290	171	263.25
84	miR-21	62	0.449	0.494	-0.104	253	243	304	263.25
85	Non-switched memory B-cells	54	0.458	0.610	0.234	281	284	210	264
86	Naive $CD4^+CD27^-CD28^-$ cells	54	0.481	0.821	1.145	349	350	28	269
87	EM $CD8^+CD57^+$ cells	54	0.469	0.710	0.292	318	316	166	279.5
88	let-7i	62	0.543	0.564	0.090	277	263	310	281.75
89	EM $CD8^+CD27^+$ cells	54	0.553	0.508	0.004	248	248	397	285.25
90	IL12p70	62	0.459	0.587	0.107	285	273	302	286.25
91	Naive $CD4^+CD28^+$ cells	54	0.538	0.638	-0.132	292	293	286	290.75
92	CM $CD4^+CD57^+$ cells	54	0.473	0.741	0.246	328	328	194	294.5
93	Free active TGF-β1	54	0.539	0.635	-0.091	290	292	309	295.25
94	sCD25	62	0.458	0.580	0.035	282	268	364	299
95	miR-20a	62	0.459	0.583	0.039	284	270	361	299.75
96	EM $CD8^+$ cells	54	0.544	0.586	0.017	274	272	382	300.5
97	CM $CD8^+CD57^+$ cells	54	0.512	0.889	0.463	374	371	84	300.75
98	CM $CD4^+CD27^-CD28^-$ cells	54	0.483	0.827	0.317	354	351	147	301.5
99	Classical monocytes	54	0.458	0.610	0.034	280	283	365	302
100	CM $CD4^+$ cells	54	0.536	0.660	-0.089	300	299	312	302.75
101	Naive $CD4^+CD27^+$ cells	54	0.535	0.672	-0.107	303	304	303	303.25
102	CM $CD4^+CD27^+CD28^+$ cells	54	0.540	0.620	-0.045	286	289	354	303.75
103	$CD4^+$ Tregs	54	0.536	0.657	-0.084	299	298	320	304
104	CM $CD8^+CD27^-CD28^-$ cells	54	0.487	0.875	0.359	367	359	124	304.25
105	IL-27	62	0.468	0.672	0.110	313	305	300	307.75
106	Naive $CD4^+$ cells	54	0.532	0.698	-0.109	312	311	301	309
107	CM $CD4^+CD27^+$ cells	54	0.538	0.647	-0.046	294	297	352	309.25
108	Class-switched memory B-cells	54	0.467	0.682	0.074	305	308	326	311
109	IL-10	62	0.460	0.597	0.003	288	279	398	313.25
110	miR-146a	62	0.481	0.800	0.203	347	344	228	316.5
111	Tumor size	62	0.526	0.727	-0.089	329	324	313	323.75
112	miR-195	62	0.486	0.855	-0.212	362	357	222	325.75
113	TEMRA $CD4^+$ cells	54	0.494	0.952	0.334	389	389	137	326
114	Naive $CD8^+CD57^+$ cells	54	0.499	1.000	0.398	401	401	105	327
115	let-7e	62	0.524	0.751	-0.079	335	331	323	331
116	Myeloid dendritic cells	54	0.472	0.734	-0.050	326	327	350	332.25
117	Naive $CD8^+CD27^-CD28^-$ cells	54	0.503	0.972	0.312	393	394	154	333.5
118	PD-L2	62	0.528	0.711	-0.031	324	317	370	333.75
119	TIM-3	62	0.503	0.972	-0.312	395	393	153	334
120	miR-181a	62	0.488	0.877	0.188	372	361	242	336.75
121	IL-6	62	0.482	0.816	0.096	352	348	306	339.5
122	EM $CD4^+$ cells	54	0.475	0.761	0.026	333	333	377	344
123	IGF-1	62	0.519	0.807	-0.034	348	346	367	352.25
124	miR-125b	62	0.512	0.877	-0.093	371	360	308	352.5
125	CRP	62	0.511	0.883	-0.125	376	368	293	353.25
126	miR-18a	62	0.480	0.794	0.000	346	343	402	359.25
127	Intermediate monocytes	54	0.489	0.897	-0.089	377	375	311	360

128	miR-19b	62	0.484	0.838	-0.003	357	353	399	366.5
129	EM $CD4^{+}CD28^{+}$ cells	54	0.508	0.924	-0.069	385	385	336	372.75
130	miR-223	62	0.492	0.921	0.043	386	382	356	377.5
131	miR-424	62	0.491	0.910	0.027	384	380	376	381
132	miR-155	62	0.491	0.910	-0.020	383	381	379	381.5
133	miR-17	62	0.497	0.978	-0.012	397	396	388	394.5
134	$CD56^{dim}CD16^{+}$ NK-cells	54	0.501	0.993	-0.006	399	399	391	397
				**LOW CD8 INFILTRATION**					
1	EM $CD4^{+}CD57^{+}$ cells	54	0.745	0.006	-1.361	8	9	21	11.5
2	CD4/CD8 ratio	54	0.227	0.002	0.879	3	4	37	11.75
3	EM $CD4^{+}CD27^{-}CD28^{-}$ cells	54	0.748	0.005	-1.195	7	8	27	12.25
4	$CD4^{+}$ cells	54	0.178	0.000	0.656	1	2	56	15
5	CM $CD8^{+}CD28^{+}$ cells	54	0.258	0.006	0.893	9	10	36	16
6	Tregs	54	0.248	0.005	0.741	6	6	48	16.5
7	CM $CD8^{+}$ cells	54	0.268	0.009	0.843	11	15	40	19.25
8	$CD4^{+}CD27^{-}CD28^{-}$ cells	54	0.710	0.018	-1.404	19	21	20	19.75
9	$CD4^{+}CD57^{+}$ cells	54	0.711	0.016	-1.321	18	20	24	20
10	Monocytes	54	0.728	0.009	-0.663	12	16	53	23.25
11	TEMRA $CD4^{+}CD57^{+}$ cells	54	0.696	0.028	-1.481	28	29	17	25.5
12	miR-195	62	0.699	0.019	-1.066	25	22	30	25.5
13	CM $CD8^{+}CD27^{+}CD28^{+}$ cells	54	0.296	0.020	0.801	21	23	41	26.5
14	TEMRA $CD4^{+}CD27^{-}CD28^{-}$ cells	54	0.689	0.034	-1.578	32	34	9	26.75
15	CM $CD8^{+}CD27^{+}$ cells	54	0.297	0.023	0.776	22	25	44	28.25
16	T-cell $P16^{INK4a}$	42	0.690	0.086	-1.508	31	66	13	35.25
17	MCP-1	62	0.241	0.002	0.334	5	5	138	38.25
18	miR-126	62	0.687	0.027	-0.603	33	28	64	39.5
19	$CD8^{+}CD57^{+}$ cells	54	0.694	0.028	-0.527	30	30	73	40.75
20	$CD8^{+}CD27^{-}CD28^{-}$ cells	54	0.685	0.036	-0.596	36	36	65	43.25
21	TEMRA $CD8^{+}CD57^{+}$ cells	54	0.679	0.044	-0.587	41	41	68	47.75
22	TEMRA $CD8^{+}$ cells	54	0.679	0.044	-0.461	40	40	86	51.5
23	$CD8^{+}CD28^{+}$ cells	54	0.299	0.023	0.327	23	26	141	53.25
24	Naive $CD8^{+}CD28^{+}$ cells	54	0.337	0.067	0.490	46	47	79	54.5
25	Naive $CD8^{+}CD27^{+}$ cells	54	0.338	0.068	0.499	47	48	77	54.75
26	TIM-3	62	0.348	0.073	0.796	61	56	42	55
27	TEMRA $CD8^{+}CD27^{-}CD28^{-}$ cells	54	0.657	0.077	-0.637	52	57	60	55.25
28	Naive $CD8^{+}CD27^{+}CD28^{+}$ cells	54	0.338	0.069	0.504	49	49	76	55.75
29	Naive B-cells	54	0.316	0.038	0.361	38	38	123	59.25
30	$CD8^{+}$ cells	54	0.662	0.069	-0.445	51	50	92	61
31	$CD8^{+}CD27^{+}CD28^{+}$ cells	54	0.336	0.065	0.382	45	46	112	62
32	Naive $CD4^{+}CD57^{+}$ cells	54	0.642	0.111	-0.909	68	78	35	62.25
33	Memory Tregs	54	0.304	0.027	0.289	27	27	168	62.25
34	NK-like T-cells	54	0.638	0.120	-0.922	71	84	33	64.75
35	Naive $CD4^{+}CD27^{-}CD28^{-}$ cells	54	0.629	0.147	-2.077	80	96	7	65.75
36	Class-switched memory B-cells	54	0.657	0.077	-0.382	53	58	110	68.5
37	Naive $CD8^{+}$ cells	54	0.347	0.086	0.441	59	65	94	69.25

38	CD4$^+$CD28$^+$ cells	54	0.275	0.011	0.162	14	17	264	77.25
39	CD8$^+$CD27$^+$ cells	54	0.347	0.086	0.321	58	64	143	80.75
40	miR-20a	62	0.654	0.070	-0.307	57	53	157	81
41	EM CD8$^+$CD27$^-$CD28$^-$ cells	54	0.631	0.142	-0.522	79	94	74	81.5
42	CD4$^+$CD27$^+$CD28$^+$ cells	54	0.330	0.055	0.236	42	43	205	83
43	miR-424	62	0.369	0.122	0.377	78	86	116	89.5
44	CD3$^+$ cells	54	0.332	0.058	0.188	44	44	243	93.75
45	CD4$^+$CD27$^+$ cells	54	0.338	0.069	0.206	50	52	225	94.25
46	Tumor grade	62	0.656	0.030	-0.196	56	32	239	95.75
47	Naive CD4$^+$CD27$^+$CD28$^+$ cells	54	0.374	0.160	0.361	90	100	122	100.5
48	sCD27	62	0.383	0.171	0.439	102	105	97	101.5
49	TEMRA CD4$^+$ cells	54	0.605	0.242	-0.782	121	142	43	106.75
50	G8 score	27	0.302	0.096	0.088	26	71	315	109.5
51	CM CD4$^+$CD28$^+$ cells	54	0.379	0.178	0.314	96	110	150	113
52	Naive Tregs	54	0.656	0.079	-0.127	55	59	292	115.25
53	Naive CD4$^+$CD28$^+$ cells	54	0.384	0.192	0.332	103	118	139	115.75
54	Non-classical monocytes	54	0.379	0.178	0.300	97	111	160	116.25
55	Lymph node involvement	62	0.595	0.197	-0.592	145	122	67	119.75
56	IL-8	62	0.361	0.102	0.163	70	76	263	119.75
57	CM CD4$^+$ cells	54	0.386	0.205	0.314	106	126	151	122.25
58	CD56^{bright}CD16$^-$ NK-cells	54	0.396	0.243	0.397	124	143	106	124.25
59	Naive CD4$^+$CD27$^+$ cells	54	0.386	0.205	0.308	107	127	156	124.25
60	miR-125b	62	0.615	0.174	-0.269	105	107	183	125
61	miR-223	62	0.599	0.247	-0.375	136	146	118	134
62	NK-cells	54	0.600	0.266	-0.336	133	154	135	138.75
63	IL-17A	62	0.576	0.372	-1.717	184	191	8	141.75
64	Intermediate monocytes	54	0.395	0.242	0.256	120	141	188	142.25
65	Naive CD4$^+$ cells	54	0.397	0.250	0.290	129	148	167	143.25
66	Age	62	0.388	0.187	0.183	109	115	246	144.75
67	EM CD8$^+$CD57$^+$ cells	54	0.586	0.338	-0.439	163	179	95	150
68	PD-1	62	0.573	0.394	-1.469	192	198	19	150.25
69	CM CD4$^+$CD27$^+$ cells	54	0.397	0.250	0.240	127	147	201	150.5
70	CM CD4$^+$CD27$^+$CD28$^+$ cells	54	0.397	0.247	0.237	128	145	203	151
71	CTLA-4	62	0.438	0.253	1.552	222	150	11	151.25
72	Classical monocytes	54	0.629	0.149	-0.061	81	97	347	151.5
73	EM CD8$^+$CD28$^+$ cells	54	0.393	0.234	0.177	117	136	252	155.5
74	miR-146a	62	0.588	0.299	-0.331	160	167	140	156.75
75	Hematopoietic stem cells	54	0.601	0.257	-0.229	131	152	213	156.75
76	miR-19a	62	0.406	0.270	0.246	149	155	195	162
77	EM CD4$^+$ cells	54	0.595	0.288	-0.246	146	163	196	162.75
78	IL-1α	62	0.408	0.281	0.234	151	158	209	167.25
79	PD-L1	62	0.566	0.440	-0.648	206	212	58	170.5
80	Plasmacytoid dendritic cells	54	0.593	0.297	-0.200	150	165	232	174.25
81	sCD25	62	0.428	0.402	0.296	196	200	162	188.5
82	Tumor size	62	0.425	0.371	0.249	186	190	192	188.5

83	Naive CD8$^+$CD57$^+$ cells	54	0.563	0.481	-0.442	217	241	93	192
84	PD-L2	62	0.417	0.334	0.137	173	177	282	201.25
85	EM CD8$^+$CD27$^+$CD28$^+$ cells	54	0.426	0.406	0.200	189	201	231	202.5
86	CD4$^+$ Tregs	54	0.410	0.315	0.073	156	172	327	202.75
87	TEMRA CD8$^+$CD27$^+$ cells	54	0.571	0.428	-0.229	198	207	212	203.75
88	miR-150	62	0.569	0.416	-0.217	199	205	220	205.75
89	CM CD4$^+$CD27$^-$CD28$^-$ cells	54	0.582	0.339	-0.114	175	180	297	206.75
90	Non-switched memory B-cells	54	0.566	0.467	-0.258	208	229	185	207.5
91	Myeloid dendritic cells	54	0.585	0.339	-0.086	168	181	317	208.5
92	IL-6	62	0.438	0.464	0.286	221	227	170	209.75
93	let-7i	62	0.411	0.296	-0.036	157	164	363	210.25
94	CD86	62	0.567	0.439	-0.205	204	210	226	211
95	miR-18a	62	0.573	0.394	-0.146	193	199	272	214.25
96	IL-17F	62	0.469	0.443	1.513	317	215	12	215.25
97	CRP	62	0.439	0.478	0.276	227	235	177	216.5
98	LAG-3	62	0.577	0.367	-0.073	182	188	329	220.25
99	Free active TGF-β1	62	0.522	0.415		342	204	3	222.75
100	miR-17	62	0.560	0.479	-0.175	230	236	253	237.25
101	4-1BB	62	0.472	0.698	-1.016	325	312	31	248.25
102	miR-155	62	0.546	0.595	-0.271	270	278	181	249.75
103	miR-92a	62	0.438	0.469	0.079	225	231	324	251.25
104	let-7e	62	0.455	0.601	0.235	273	280	208	258.5
105	CD56^{dim}CD16$^+$ NK-cells	54	0.564	0.479	-0.030	216	239	371	260.5
106	EM CD8$^+$CD27$^+$ cells	54	0.448	0.562	0.130	249	262	288	262
107	CM CD4$^+$CD57$^+$ cells	54	0.537	0.685	-0.278	297	309	174	269.25
108	Naive CD8$^+$CD27$^-$CD28$^-$ cells	54	0.529	0.750	-0.345	321	330	132	276
109	miR-19b	62	0.550	0.557	-0.071	255	261	334	276.25
110	EM CD8$^+$ cells	54	0.441	0.515	0.006	234	250	394	278
111	B-cells	54	0.443	0.528	0.014	240	255	387	280.5
112	EM CD4$^+$CD28$^+$ cells	54	0.547	0.606	-0.083	262	281	321	281.5
113	IL-27	62	0.463	0.664	0.185	295	301	244	283.75
114	IL-10	62	0.531	0.717	0.249	315	322	193	286.25
115	IP-10	62	0.469	0.717	0.144	316	323	275	307.5
116	TEMRA CD8$^+$CD27$^+$CD28$^+$ cells	54	0.524	0.794	-0.219	337	342	218	308.5
117	IL12p70	62	0.488	0.898	0.380	375	376	115	310.25
118	CM CD8$^+$CD57$^+$ cells	54	0.474	0.779	-0.180	332	336	249	312.25
119	IFN-γ	62	0.536	0.675	-0.064	301	307	342	312.75
120	miR-326	62	0.505	0.948	-0.335	390	388	136	326
121	EM CD4$^+$CD27$^+$ cells	54	0.535	0.702	-0.006	302	313	392	327.25
122	TEMRA CD4$^+$CD28$^+$ cells	54	0.513	0.894	-0.237	370	374	204	329.5
123	miR-21	62	0.530	0.729	0.045	320	326	355	330.25
124	EM CD4$^+$CD27$^+$CD28$^+$ cells	54	0.533	0.717	0.007	309	321	389	332
125	IL-1β	62	0.533	0.705	0.006	311	314	393	332.25
126	TEMRA CD4$^+$CD27$^+$ cells	54	0.526	0.779	-0.072	331	337	331	332.5
127	TNF-α	62	0.514	0.878	0.181	365	364	247	335.25

128	miR-9	62	0.484	0.829	0.141	356	352	277	335.25
129	miR-181a	62	0.493	0.936	-0.235	387	386	206	341.5
130	Gal-9	62	0.501	0.994	0.223	400	400	217	354.25
131	TEMRA $CD4^+CD27^+CD28^+$ cells	54	0.513	0.892	-0.033	369	372	369	369.75
132	IGF-1	62	0.514	0.880	-0.017	366	367	383	370.5
133	TEMRA $CD8^+CD28^+$ cells	54	0.498	0.992	-0.110	398	398	299	373.25
134	CM $CD8^+CD27^-CD28^-$ cells	54	0.505	0.961	-0.006	391	391	395	392
