# Supplementary material for: Blood Immunosenescence Signatures Reflecting Age, Frailty and Tumor Immune Infiltrate in Patients with Early Luminal Breast Cancer
Source: Cancers (Basel). 2021 May 2;13(9):2185. doi: 10.3390/cancers13092185 (PMC8125302; doi:10.3390/cancers13092185)
Supplement: Supplementary file 1 [file cancers-13-02185-s001.zip › Table S7 - Individual performance_CD8 infiltration invasive front.pdf]

Table S4: Individual performances of biomarkers correlating with CD8 infiltration in the invasive front (high, intermediate or low infiltration). The table reports the number of patients (N) for which the biomarkers could be measured. The area under the curve (AUC) via receiver operating characteristics (ROC), P-value (Wilcox rank-sum test) and log fold change (FC) are reported for each biomarker. The log FC compared case vs. control. A positive log FC indicates that the measurement is higher than its reference while a negative measurement indicates that is smaller. Based on these statistics AUC, P-value, log FC scores were computed. The final score combines the 3 scores, where AUC weighted double. The biomarkers are ranked based on their final score.

	Blood markers	N	AUC	*P*-value	log FC	AUC score	*P*-value score	log FC score	Final score
	**HIGH CD8 INFILTRATION**								
1	TEMRA CD8$^+$CD27$^+$CD28$^+$ cells	54	0.268	0.009	0.646	4	6	45	14.75
2	TEMRA CD4$^+$CD27$^+$ cells	54	0.306	0.029	0.767	16	19	37	22
3	TEMRA CD8$^+$CD27$^+$ cells	54	0.281	0.014	0.565	10	12	56	22
4	miR-195	62	0.319	0.033	1.000	22	22	23	22.25
5	TEMRA CD4$^+$CD27$^+$CD28$^+$ cells	54	0.315	0.037	0.771	18	24	36	24
6	IL-1α	62	0.723	0.009	-0.455	9	5	81	26
7	Tumor grade	62	0.200	0.000	0.357	1	1	118	30.25
8	Gal-9	62	0.652	0.073	-0.658	33	36	42	36
9	Age	62	0.728	0.007	-0.296	6	4	141	39.25
10	TIM-3	62	0.648	0.081	-0.537	36	40	59	42.75
11	TEMRA CD4$^+$CD28$^+$ cells	54	0.356	0.105	0.776	41	55	35	43
12	miR-150	62	0.358	0.094	0.610	45	48	51	47.25
13	miR-424	62	0.643	0.093	-0.474	44	46	75	52.25
14	miR-125b	62	0.369	0.122	0.357	60	67	119	76.5
15	sCD25	62	0.626	0.138	-0.381	71	70	107	79.75
16	B-cells	54	0.634	0.133	-0.276	57	69	156	84.75
17	4-1BB	62	0.417	0.241	2.115	142	107	5	99
18	EM CD4$^+$CD27$^-$CD28$^-$ cells	54	0.391	0.220	0.375	93	101	112	99.75
19	Class-switched memory B-cells	54	0.384	0.192	0.257	86	91	163	106.5
20	IFN-γ	62	0.591	0.284	-0.486	125	126	67	110.75
21	Monocytes	54	0.374	0.160	0.164	72	82	236	115.5
22	IP-10	62	0.607	0.210	-0.243	96	98	173	115.75
23	CD86	62	0.598	0.253	-0.324	109	112	134	116
24	TEMRA CD8$^+$CD28$^+$ cells	54	0.396	0.243	0.252	99	109	167	118.5
25	TEMRA CD4$^+$ cells	54	0.402	0.274	0.258	107	119	161	123.5
26	miR-20a	62	0.400	0.240	0.232	103	106	182	123.5
27	IL-6	62	0.586	0.315	-0.460	136	143	80	123.75
28	Intermediate monocytes	54	0.619	0.185	-0.160	84	89	240	124.25
29	EM CD4$^+$CD27$^+$CD28$^+$ cells	54	0.403	0.283	0.243	112	124	172	130
30	TEMRA CD4$^+$CD27$^-$CD28$^-$ cells	54	0.417	0.354	-0.426	141	160	95	134.25
31	miR-223	62	0.412	0.299	0.282	130	133	153	136.5
32	EM CD4$^+$CD27$^+$ cells	54	0.409	0.310	0.240	123	140	177	140.75
33	TNF-α	62	0.573	0.393	-0.652	172	179	44	141.75
34	IL-27	62	0.579	0.355	-0.355	153	161	121	147
35	miR-326	62	0.579	0.275	-0.257	152	122	164	147.5
36	Naive Tregs	54	0.602	0.258	-0.085	102	116	304	156
37	Naive B-cells	54	0.590	0.319	-0.165	127	144	235	158.25

38	IL12p70	62	0.565	0.449	-0.606	197	211	52	164.25
39	$CD8^+$ cells	54	0.412	0.328	0.156	133	148	245	164.75
40	TEMRA $CD4^+CD57^+$ cells	54	0.433	0.457	-0.474	188	216	74	166.5
41	TEMRA $CD8^+$ cells	54	0.414	0.334	0.154	135	152	246	167
42	CM $CD8^+$ cells	54	0.568	0.451	-0.371	181	213	114	172.25
43	Naive $CD4^+CD27^-CD28^-$ cells	54	0.394	0.235	-0.001	97	105	401	175
44	MCP-1	62	0.583	0.330	-0.128	143	149	267	175.5
45	CM $CD4^+CD28^+$ cells	54	0.573	0.421	-0.226	173	195	184	181.25
46	Memory Tregs	54	0.421	0.378	0.141	151	169	256	181.75
47	CM $CD4^+$ cells	54	0.569	0.444	-0.227	176	205	183	185
48	Naive $CD4^+CD27^+$ cells	54	0.571	0.433	-0.197	175	199	208	189.25
49	miR-19a	62	0.426	0.385	0.154	169	173	247	189.5
50	miR-126	62	0.437	0.459	0.349	207	217	128	189.75
51	Naive $CD4^+CD28^+$ cells	54	0.569	0.440	-0.192	177	201	214	192.25
52	miR-9	62	0.453	0.522	1.358	262	241	17	195.5
53	CM $CD4^+CD27^-CD28^-$ cells	54	0.439	0.481	-0.296	218	227	142	201.25
54	$CD4^+CD28^+$ cells	54	0.588	0.325	0.001	132	145	402	202.75
55	miR-92a	62	0.438	0.469	0.240	214	221	175	206
56	EM $CD4^+$ cells	54	0.436	0.475	0.198	199	223	203	206
57	CM $CD8^+CD28^+$ cells	54	0.556	0.530	-0.354	228	247	123	206.5
58	Naive $CD4^+CD27^+CD28^+$ cells	54	0.564	0.479	-0.198	201	226	207	208.75
59	$CD4^+CD27^-CD28^-$ cells	54	0.422	0.385	-0.042	156	171	354	209.25
60	IL-17F	62	0.469	0.443	1.502	314	203	14	211.25
61	IL-17A	62	0.460	0.641	2.086	280	285	6	212.75
62	Naive $CD4^+$ cells	54	0.564	0.479	-0.174	200	225	227	213
63	NK-like T-cells	54	0.450	0.582	0.462	253	269	78	213.25
64	PD-1	62	0.457	0.612	-0.844	274	278	30	214
65	Classical monocytes	54	0.422	0.389	0.018	157	174	382	217.5
66	Free active TGF-β1	62	0.522	0.415		340	192	2	218.5
67	CM $CD8^+CD27^+CD28^+$ cells	54	0.550	0.579	-0.358	247	268	117	219.75
68	CM $CD4^+CD27^+$ cells	54	0.557	0.528	-0.198	223	243	205	223.5
69	miR-17	62	0.443	0.504	0.181	226	232	223	226.75
70	EM $CD4^+CD57^+$ cells	54	0.454	0.609	0.404	266	276	101	227.25
71	IL-8	62	0.565	0.449	-0.075	198	212	315	230.75
72	CM $CD8^+CD27^+$ cells	54	0.545	0.616	-0.376	269	280	111	232.25
73	EM $CD4^+CD28^+$ cells	54	0.443	0.528	0.163	224	244	238	232.5
74	CM $CD4^+CD27^+CD28^+$ cells	54	0.554	0.549	-0.196	234	257	212	234.25
75	Naive $CD8^+CD57^+$ cells	54	0.438	0.487	-0.085	211	229	303	238.5
76	TEMRA $CD8^+CD57^+$ cells	54	0.443	0.528	0.137	225	245	260	238.75
77	Tregs	54	0.559	0.511	-0.111	220	235	280	238.75
78	$CD56^{dim}CD16^+$ NK-cells	54	0.434	0.467	0.038	191	220	360	240.5
79	miR-146a	62	0.437	0.459	0.047	208	218	348	245.5
80	Myeloid dendritic cells	54	0.449	0.569	0.143	246	265	253	252.5
81	$CD3^+$ cells	54	0.440	0.505	0.053	219	233	343	253.5
82	EM $CD8^+CD27^-CD28^-$ cells	54	0.436	0.475	-0.007	202	224	395	255.75

83	Hematopoietic stem cells	54	0.533	0.713	-0.319	301	311	136	262.25
84	miR-181a	62	0.453	0.584	0.139	261	270	259	262.75
85	miR-155	62	0.466	0.693	0.273	295	304	158	263
86	IL-1β	62	0.529	0.741	-0.439	324	326	88	265.5
87	CRP	62	0.546	0.593	-0.128	264	274	268	267.5
88	Tumor size	62	0.541	0.626	-0.147	279	283	249	272.5
89	PD-L2	62	0.546	0.593	-0.102	265	275	285	272.5
90	Non-classical monocytes	54	0.552	0.566	-0.015	239	263	387	282
91	EM $CD8^+CD57^+$ cells	54	0.451	0.592	0.046	255	273	349	283
92	TEMRA $CD8^+CD27^-CD28^-$ cells	54	0.461	0.664	0.096	281	294	292	287
93	$CD4^+CD57^+$ cells	54	0.451	0.592	-0.028	254	272	371	287.75
94	sCD27	62	0.457	0.616	-0.065	275	281	328	289.75
95	$CD8^+CD57^+$ cells	54	0.462	0.674	0.088	282	299	300	290.75
96	miR-21	62	0.537	0.670	0.089	287	298	298	292.5
97	$CD4^+$ Tregs	54	0.545	0.616	0.029	271	279	368	297.25
98	IL-10	62	0.520	0.815	-0.276	346	347	155	298.5
99	IGF-1	62	0.462	0.661	0.061	283	292	336	298.5
100	$CD4^+CD27^+$ cells	54	0.542	0.643	-0.034	277	286	362	300.5
101	CM $CD8^+CD27^-CD28^-$ cells	54	0.511	0.908	-0.476	377	378	72	301
102	$CD4^+CD27^+CD28^+$ cells	54	0.542	0.646	-0.033	278	288	363	301.75
103	CD4/CD8 ratio	54	0.526	0.779	-0.183	329	338	218	303.5
104	let-7i	62	0.533	0.705	-0.083	304	310	306	306
105	CM $CD8^+CD57^+$ cells	54	0.511	0.908	-0.431	378	379	92	306.75
106	Naive $CD8^+CD27^-CD28^-$ cells	54	0.467	0.714	-0.061	300	312	334	311.5
107	T-cell $P16^{INK4a}$	42	0.500	1.000	-0.704	402	402	41	311.75
108	PD-L1	62	0.482	0.841	0.198	354	354	206	317
109	Plasmacytoid dendritic cells	54	0.479	0.817	-0.163	342	350	237	317.75
110	miR-19b	62	0.470	0.729	0.073	317	319	318	317.75
111	Naive $CD8^+$ cells	54	0.525	0.789	-0.124	331	340	271	318.25
112	$CD8^+CD27^+CD28^+$ cells	54	0.472	0.760	0.065	326	330	327	327.25
113	Naive $CD8^+CD27^+$ cells	54	0.515	0.877	-0.179	364	364	225	329.25
114	EM $CD8^+CD27^+CD28^+$ cells	54	0.482	0.847	-0.127	352	356	269	332.25
115	Naive $CD4^+CD57^+$ cells	54	0.476	0.794	-0.069	333	342	323	332.75
116	let-7e	62	0.514	0.879	-0.144	368	365	251	338
117	EM $CD8^+CD27^+$ cells	54	0.486	0.885	-0.140	366	367	257	339
118	$CD8^+CD28^+$ cells	54	0.523	0.804	-0.039	336	346	359	344.25
119	Naive $CD8^+CD27^+CD28^+$ cells	54	0.509	0.924	-0.168	383	384	232	345.5
120	Naive $CD8^+CD28^+$ cells	54	0.513	0.892	-0.117	370	370	277	346.75
121	miR-18a	62	0.523	0.791	-0.020	335	341	379	347.5
122	LAG-3	62	0.503	0.981	-0.208	399	399	197	348.5
123	$CD8^+CD27^-CD28^-$ cells	54	0.515	0.877	-0.071	363	363	321	352.5
124	$CD8^+CD27^+$ cells	54	0.515	0.870	-0.062	360	361	331	353
125	CTLA-4	62	0.483	0.763	-0.017	355	333	384	356.75
126	NK-cells	54	0.518	0.849	-0.022	351	357	377	359
127	Non-switched memory B-cells	54	0.485	0.879	-0.043	362	366	353	360.75

128	Geriatric_G8score	27	0.479	0.937	-0.028	345	388	370	362
129	CD56^{bright}CD16^{-} NK-cells	54	0.512	0.900	-0.063	373	377	330	363.25
130	Lymph node involvement	62	0.511	0.889	-0.061	379	369	335	365.5
131	CM CD4^{+}CD57^{+} cells	54	0.497	0.977	-0.040	397	398	358	387.5
132	CD4^{+} cells	54	0.491	0.924	0.004	384	385	400	388.25
133	EM CD8^{+} cells	54	0.504	0.970	-0.027	394	396	372	389
134	EM CD8^{+}CD28^{+} cells	54	0.494	0.954	-0.016	392	393	386	390.75
**INTERMEDIATE CD8 INFILTRATION**									
1	CD4/CD8 ratio	54	0.678	0.026	-0.626	25	18	50	30
2	CD4^{+} cells	54	0.689	0.017	-0.439	17	15	89	35
3	TEMRA CD8^{+}CD27^{+}CD28^{+} cells	54	0.682	0.023	-0.451	21	17	83	36
4	T-cell *P16^{INK4a}*	42	0.356	0.113	1.373	43	58	16	40
5	sCD27	62	0.674	0.018	-0.382	26	16	106	44
6	CM CD8^{+}CD28^{+} cells	54	0.638	0.085	-0.591	49	42	53	48
7	CM CD8^{+}CD27^{+}CD28^{+} cells	54	0.636	0.090	-0.572	54	44	55	52
8	CM CD8^{+}CD27^{+} cells	54	0.638	0.086	-0.537	52	43	60	52
9	TEMRA CD8^{+}CD27^{+} cells	54	0.651	0.059	-0.379	34	32	108	52
10	TEMRA CD4^{+}CD27^{+} cells	54	0.628	0.109	-0.643	68	57	46	60
11	CM CD8^{+} cells	54	0.631	0.104	-0.538	65	52	58	60
12	Memory Tregs	54	0.661	0.044	-0.271	29	27	159	61
13	TEMRA CD4^{+}CD27^{+}CD28^{+} cells	54	0.625	0.119	-0.658	74	62	43	63
14	CD86	62	0.369	0.077	0.419	59	38	98	64
15	Lymph node involvement	62	0.386	0.077	0.641	89	39	47	66
16	Tregs	54	0.624	0.121	-0.480	78	65	71	73
17	MCP-1	62	0.646	0.049	-0.190	38	29	215	80
18	miR-19a	62	0.626	0.091	-0.301	73	45	138	82
19	NK-cells	54	0.375	0.120	0.323	75	63	135	87
20	miR-150	62	0.606	0.155	-0.446	98	80	86	91
21	PD-L1	62	0.393	0.151	0.378	95	79	110	95
22	miR-92a	62	0.615	0.121	-0.291	87	64	146	96
23	Naive Tregs	54	0.353	0.066	0.119	37	35	275	96
24	TEMRA CD4^{+}CD28^{+} cells	54	0.592	0.257	-0.497	122	115	64	106
25	CRP	62	0.595	0.204	-0.399	115	96	102	107
26	CD3^{+} cells	54	0.627	0.113	-0.140	69	59	258	114
27	Tumor grade	62	0.610	0.081	-0.145	92	41	250	119
28	CD8^{+}CD27^{-}CD28^{-} cells	54	0.406	0.242	0.287	117	108	149	123
29	PD-1	62	0.427	0.327	1.588	171	147	10	125
30	CD8^{+}CD27^{+} cells	54	0.599	0.216	-0.199	104	100	201	127
31	Naive CD8^{+}CD27^{-}CD28^{-} cells	54	0.583	0.304	-0.378	144	137	109	134
32	CTLA-4	62	0.561	0.202	-1.576	217	93	11	135
33	CD8^{+}CD28^{+} cells	54	0.599	0.222	-0.169	105	103	231	136
34	CD8^{+}CD27^{+}CD28^{+} cells	54	0.590	0.265	-0.211	129	118	195	143
35	TEMRA CD8^{+}CD27^{-}CD28^{-} cells	54	0.418	0.309	0.295	146	139	144	144
36	Hematopoietic stem cells	54	0.422	0.332	0.368	155	151	115	144
37	Naive CD8^{+}CD57^{+} cells	54	0.576	0.347	-0.421	162	158	97	145

38	IFN-γ	62	0.424	0.306	0.330	161	138	131	148
39	TEMRA $CD8^+CD57^+$ cells	54	0.418	0.311	0.248	147	142	170	152
40	IL-17F	62	0.548	0.175	-7.396	259	86	4	152
41	TEMRA $CD8^+CD28^+$ cells	54	0.591	0.258	-0.130	126	117	266	159
42	Age	62	0.405	0.202	0.075	116	94	312	160
43	miR-223	62	0.577	0.304	-0.221	160	136	189	161
44	G8 score	27	0.619	0.293	-0.041	83	129	357	163
45	4-1BB	62	0.553	0.392	-1.316	235	178	18	167
46	Free active TGF-β1	62	0.467	0.147		298	76	1	168
47	miR-326	62	0.438	0.331	0.390	213	150	103	170
48	CM $CD8^+CD57^+$ cells	54	0.594	0.243	-0.062	118	110	333	170
49	miR-155	62	0.564	0.390	-0.383	204	177	105	173
50	CM $CD4^+CD27^-CD28^-$ cells	54	0.557	0.462	-0.889	227	219	28	175
51	EM $CD8^+CD27^+CD28^+$ cells	54	0.585	0.292	-0.087	137	128	302	176
52	$CD8^+CD57^+$ cells	54	0.425	0.355	0.184	165	162	217	177
53	$CD8^+$ cells	54	0.425	0.355	0.182	166	163	219	179
54	miR-17	62	0.566	0.375	-0.221	190	167	188	184
55	miR-9	62	0.548	0.449	-1.481	258	209	15	185
56	TEMRA $CD4^+CD57^+$ cells	54	0.448	0.519	0.812	241	240	33	189
57	Naive $CD4^+CD27^+$ cells	54	0.432	0.402	0.193	179	184	213	189
58	Non-switched memory B-cells	54	0.436	0.431	0.288	206	198	148	190
59	let-7e	62	0.564	0.390	-0.240	203	176	176	190
60	Gal-9	62	0.433	0.374	0.181	187	166	221	190
61	Naive $CD4^+CD28^+$ cells	54	0.433	0.403	0.185	184	187	216	193
62	B-cells	54	0.436	0.431	0.198	205	197	204	203
63	EM $CD8^+CD27^+$ cells	54	0.574	0.356	-0.068	168	165	324	206
64	$CD4^+CD57^+$ cells	54	0.447	0.517	0.355	238	238	120	209
65	EM $CD8^+CD28^+$ cells	54	0.568	0.402	-0.090	180	185	296	210
66	Naive $CD4^+$ cells	54	0.438	0.442	0.166	209	202	234	214
67	IL-1α	62	0.435	0.382	0.079	195	170	309	217
68	Naive $CD8^+CD27^+$ cells	54	0.567	0.403	-0.067	185	188	326	221
69	CM $CD4^+CD27^+CD28^+$ cells	54	0.554	0.503	-0.217	232	231	192	222
70	Naive $CD8^+CD28^+$ cells	54	0.565	0.418	-0.075	192	194	314	223
71	EM $CD4^+CD27^+CD28^+$ cells	54	0.553	0.517	-0.220	237	237	190	225
72	Naive $CD8^+CD27^+CD28^+$ cells	54	0.567	0.411	-0.056	186	191	340	226
73	CM $CD4^+CD57^+$ cells	54	0.547	0.560	-0.350	260	261	126	227
74	IL-17A	62	0.467	0.657	-1.045	299	290	22	228
75	CM $CD4^+$ cells	54	0.550	0.540	-0.252	248	252	166	229
76	TEMRA $CD4^+CD27^-CD28^-$ cells	54	0.466	0.676	0.830	294	300	31	230
77	CM $CD4^+CD27^+$ cells	54	0.551	0.528	-0.218	243	246	191	231
78	LAG-3	62	0.448	0.488	0.196	242	230	209	231
79	Naive $CD4^+CD27^+CD28^+$ cells	54	0.446	0.506	0.174	233	234	228	232
80	Plasmacytoid dendritic cells	54	0.450	0.536	0.240	251	249	178	232
81	CM $CD4^+CD28^+$ cells	54	0.549	0.551	-0.248	256	258	169	235
82	EM $CD4^+CD27^-CD28^-$ cells	54	0.439	0.449	-0.090	216	210	297	235

83	miR-20a	62	0.439	0.410	0.071	215	189	322	235
84	Naive $CD4^+CD27^-CD28^-$ cells	54	0.565	0.423	0.041	196	196	356	236
85	EM $CD4^+CD57^+$ cells	54	0.433	0.403	-0.009	183	186	392	236
86	Naive $CD8^+$ cells	54	0.558	0.473	-0.101	222	222	286	238
87	$CD4^+CD27^-CD28^-$ cells	54	0.463	0.645	0.388	285	287	104	240
88	EM $CD4^+CD27^+$ cells	54	0.549	0.551	-0.209	257	259	196	242
89	TEMRA $CD8^+$ cells	54	0.449	0.525	0.152	244	242	248	245
90	sCD25	62	0.444	0.454	0.081	229	215	308	245
91	CM $CD8^+CD27^-CD28^-$ cells	54	0.552	0.519	0.107	240	239	283	251
92	TEMRA $CD4^+$ cells	54	0.550	0.540	0.136	249	253	261	253
93	NK-like T-cells	54	0.450	0.537	0.131	250	250	265	254
94	IL-27	62	0.454	0.535	0.132	263	248	263	259
95	Monocytes	54	0.464	0.660	0.239	289	291	179	262
96	miR-146a	62	0.546	0.540	-0.126	268	254	270	265
97	Naive B-cells	54	0.546	0.575	-0.111	267	267	282	271
98	TNF-α	62	0.467	0.662	0.214	303	293	193	273
99	IL-1β	62	0.471	0.703	0.254	322	308	165	279
100	EM $CD8^+CD27^-CD28^-$ cells	54	0.476	0.774	0.350	334	335	127	283
101	Myeloid dendritic cells	54	0.532	0.695	-0.182	308	306	220	286
102	let-7i	62	0.538	0.612	0.077	284	277	310	289
103	miR-181a	62	0.544	0.554	-0.030	273	260	364	293
104	$CD4^+$ Tregs	54	0.532	0.695	-0.133	307	305	262	295
105	IL-6	62	0.542	0.573	-0.028	276	266	369	297
106	EM $CD8^+$ cells	54	0.544	0.586	0.018	272	271	383	300
107	miR-424	62	0.466	0.652	0.072	296	289	320	300
108	miR-195	62	0.477	0.762	-0.199	337	331	202	302
109	Class-switched memory B-cells	54	0.533	0.682	-0.074	297	301	316	303
110	miR-19b	62	0.468	0.667	0.073	306	295	319	307
111	$CD4^+CD28^+$ cells	54	0.535	0.670	-0.051	293	297	344	307
112	miR-126	62	0.464	0.632	-0.015	290	284	389	313
113	EM $CD4^+$ cells	54	0.528	0.728	-0.088	325	316	301	317
114	TIM-3	62	0.490	0.894	-0.283	380	373	152	321
115	miR-21	62	0.481	0.800	-0.143	349	344	252	324
116	IL-8	62	0.529	0.704	-0.030	323	309	366	330
117	EM $CD8^+CD57^+$ cells	54	0.514	0.870	0.172	365	362	230	331
118	$CD4^+CD27^+CD28^+$ cells	54	0.526	0.749	-0.055	328	328	341	331
119	EM $CD4^+CD28^+$ cells	54	0.519	0.816	-0.092	348	348	295	335
120	miR-125b	62	0.518	0.816	-0.100	353	349	287	336
121	Naive $CD4^+CD57^+$ cells	54	0.478	0.787	0.044	338	339	351	342
122	IGF-1	62	0.522	0.774	-0.030	339	336	367	345
123	$CD56^{dim}CD16^+$ NK-cells	54	0.525	0.763	-0.012	330	332	391	346
124	miR-18a	62	0.476	0.746	0.009	332	327	393	346
125	$CD4^+CD27^+$ cells	54	0.521	0.801	-0.041	344	345	355	347
126	IL12p70	62	0.479	0.778	0.030	343	337	365	347
127	IP-10	62	0.480	0.794	0.020	347	343	378	354

128	Intermediate monocytes	54	0.508	0.924	-0.119	387	386	276	359
129	Classical monocytes	54	0.483	0.843	0.025	356	355	373	360
130	Non-classical monocytes	54	0.494	0.952	-0.123	393	392	274	363
131	PD-L2	62	0.516	0.839	0.004	358	353	399	367
132	$CD56^{bright}CD16^{-}$ NK-cells	54	0.485	0.855	-0.006	361	359	397	370
133	IL-10	62	0.496	0.966	-0.083	396	394	307	373
134	Tumor size	62	0.491	0.909	-0.045	385	380	350	375
**LOW CD8 INFILTRATION**									
1	CD4/CD8 ratio	54	0.255	0.006	0.827	3	3	32	10.25
2	CM $CD8^{+}CD28^{+}$ cells	54	0.274	0.011	0.924	7	8	24	11.5
3	CM $CD8^{+}$ cells	54	0.272	0.010	0.887	5	7	29	11.5
4	CM $CD8^{+}CD27^{+}CD28^{+}$ cells	54	0.282	0.013	0.909	11	11	26	14.75
5	miR-195	62	0.711	0.013	-1.093	15	10	21	15.25
6	CM $CD8^{+}CD27^{+}$ cells	54	0.285	0.016	0.889	12	13	27	16
7	T-cell $P16^{INK4a}$	42	0.714	0.053	-1.623	13	30	9	16.25
8	Tregs	54	0.288	0.017	0.629	14	14	49	22.75
9	$CD4^{+}$ cells	54	0.277	0.011	0.489	8	9	66	22.75
10	sCD27	62	0.317	0.029	0.515	20	20	61	30.25
11	PD-L1	62	0.657	0.064	-0.808	31	34	34	32.5
12	MCP-1	62	0.226	0.001	0.352	2	2	124	32.5
13	CRP	62	0.330	0.044	0.586	28	28	54	34.5
14	Monocytes	54	0.670	0.055	-0.506	27	31	62	36.75
15	PD-1	62	0.639	0.102	-1.653	48	51	8	38.75
16	Lymph node involvement	62	0.637	0.062	-0.909	53	33	25	41
17	TEMRA $CD8^{+}CD57^{+}$ cells	54	0.658	0.075	-0.485	30	37	69	41.5
18	$CD8^{+}$ cells	54	0.680	0.042	-0.412	23	26	99	42.75
19	miR-20a	62	0.680	0.034	-0.355	24	23	122	48.25
20	EM $CD4^{+}CD27^{-}CD28^{-}$ cells	54	0.685	0.038	-0.299	19	25	140	50.75
21	TIM-3	62	0.365	0.114	0.765	56	60	38	52.5
22	$CD8^{+}CD27^{-}CD28^{-}$ cells	54	0.644	0.105	-0.455	40	54	82	54
23	TEMRA $CD8^{+}CD27^{-}CD28^{-}$ cells	54	0.640	0.115	-0.503	47	61	63	54.5
24	IL-6	62	0.359	0.097	0.431	46	49	91	58
25	TEMRA $CD8^{+}$ cells	54	0.650	0.093	-0.368	35	47	116	58.25
26	TEMRA $CD4^{+}CD57^{+}$ cells	54	0.631	0.142	-0.630	63	72	48	61.5
27	CM $CD4^{+}CD28^{+}$ cells	54	0.368	0.138	0.486	58	71	68	63.75
28	CM $CD4^{+}$ cells	54	0.369	0.143	0.491	62	74	65	65.75
29	TEMRA $CD4^{+}CD27^{-}CD28^{-}$ cells	54	0.625	0.161	-0.711	76	83	40	68.75
30	NK-cells	54	0.636	0.128	-0.405	55	68	100	69.5
31	Naive B-cells	54	0.354	0.101	0.284	39	50	151	69.75
32	IL-1α	62	0.362	0.104	0.301	50	53	139	73
33	EM $CD4^{+}CD57^{+}$ cells	54	0.629	0.147	-0.449	66	77	84	73.25
34	Tumor grade	62	0.656	0.030	-0.196	32	21	210	73.75
35	CM $CD8^{+}CD57^{+}$ cells	54	0.374	0.156	0.448	70	81	85	76.5
36	$CD8^{+}CD27^{+}$ cells	54	0.362	0.122	0.292	51	66	145	78.25
37	$CD4^{+}CD27^{-}CD28^{-}$ cells	54	0.624	0.164	-0.476	79	85	73	79

38	NK-like T-cells	54	0.611	0.213	-0.744	90	99	39	79.5
39	$CD8^+CD28^+$ cells	54	0.356	0.105	0.236	42	56	181	80.25
40	$CD8^+CD57^+$ cells	54	0.631	0.143	-0.336	64	75	129	83
41	CM $CD4^+CD27^+$ cells	54	0.379	0.178	0.430	81	88	93	85.75
42	CM $CD4^+CD27^+CD28^+$ cells	54	0.379	0.176	0.428	82	87	94	86.25
43	Intermediate monocytes	54	0.371	0.149	0.289	67	78	147	89.75
44	$CD4^+CD57^+$ cells	54	0.614	0.205	-0.445	88	97	87	90
45	$CD8^+CD27^+CD28^+$ cells	54	0.375	0.161	0.313	77	84	137	93.75
46	miR-126	62	0.610	0.195	-0.373	91	92	113	96.75
47	IL-17A	62	0.584	0.326	-1.793	139	146	7	107.75
48	let-7e	62	0.403	0.253	0.423	111	114	96	108
49	miR-125b	62	0.608	0.204	-0.258	94	95	162	111.25
50	EM $CD8^+CD27^-CD28^-$ cells	54	0.593	0.297	-0.462	119	130	79	111.75
51	miR-424	62	0.401	0.247	0.324	106	111	133	114
52	Memory Tregs	54	0.381	0.185	0.180	85	90	224	121
53	Non-switched memory B-cells	54	0.593	0.301	-0.332	121	134	130	126.5
54	Naive $CD8^+CD27^+$ cells	54	0.403	0.275	0.247	110	121	171	128
55	$CD4^+CD28^+$ cells	54	0.369	0.142	0.062	61	73	332	131.75
56	Age	62	0.396	0.221	0.178	100	102	226	132
57	Naive $CD8^+$ cells	54	0.403	0.283	0.237	113	125	180	132.75
58	CTLA-4	62	0.438	0.253	1.552	210	113	12	136.25
59	miR-19a	62	0.410	0.292	0.224	128	127	187	142.5
60	Naive $CD8^+CD28^+$ cells	54	0.407	0.297	0.201	120	131	199	142.5
61	Naive $CD8^+CD27^+CD28^+$ cells	54	0.409	0.310	0.225	124	141	185	143.5
62	IL-8	62	0.398	0.231	0.111	101	104	281	146.75
63	EM $CD8^+CD27^+CD28^+$ cells	54	0.414	0.334	0.224	134	153	186	151.75
64	$CD3^+$ cells	54	0.403	0.279	0.115	114	123	278	157.25
65	Naive $CD8^+CD27^-CD28^-$ cells	54	0.432	0.445	0.483	178	206	70	158
66	G8 score	27	0.377	0.303	0.057	80	135	338	158.25
67	CM $CD8^+CD27^-CD28^-$ cells	54	0.425	0.401	0.295	163	182	143	162.75
68	IP-10	62	0.419	0.343	0.200	148	156	200	163
69	miR-92a	62	0.412	0.299	0.124	131	132	272	166.5
70	EM $CD8^+CD27^+$ cells	54	0.422	0.385	0.214	158	172	194	170.5
71	Classical monocytes	54	0.598	0.274	-0.049	108	120	346	170.5
72	CD86	62	0.573	0.393	-0.250	170	180	168	172
73	Plasmacytoid dendritic cells	54	0.583	0.354	-0.141	140	159	255	173.5
74	$CD4^+$ Tregs	54	0.415	0.344	0.131	138	157	264	174.25
75	miR-18a	62	0.579	0.355	-0.157	154	164	242	178.5
76	Gal-9	62	0.435	0.449	0.329	193	207	132	181.25
77	Class-switched memory B-cells	54	0.575	0.401	-0.181	164	183	222	183.25
78	miR-19b	62	0.572	0.398	-0.173	174	181	229	189.5
79	EM $CD8^+CD28^+$ cells	54	0.422	0.389	0.124	159	175	273	191.5
80	miR-181a	62	0.419	0.342	0.059	149	155	337	197.5
81	Naive Tregs	54	0.579	0.378	-0.064	150	168	329	199.25
82	CM $CD4^+CD57^+$ cells	54	0.445	0.543	0.435	231	256	90	202

83	CD4+CD27+CD28+ cells	54	0.426	0.410	0.100	167	190	289	203.25
84	let-7i	62	0.418	0.334	-0.019	145	154	380	206
85	IL-17F	62	0.469	0.443	1.513	315	204	13	211.75
86	PD-L2	62	0.433	0.439	0.094	189	200	294	218
87	Free active TGF-β1	62	0.522	0.415		341	193	3	219.5
88	CD4+CD27+ cells	54	0.432	0.451	0.084	182	214	305	220.75
89	Hematopoietic stem cells	54	0.562	0.485	-0.158	212	228	241	223.25
90	sCD25	62	0.447	0.539	0.241	236	251	174	224.25
91	LAG-3	62	0.565	0.449	-0.057	194	208	339	233.75
92	IL12p70	62	0.463	0.670	0.469	288	296	77	237.25
93	TEMRA CD4+ cells	54	0.537	0.688	-0.473	286	302	76	237.5
94	miR-155	62	0.450	0.562	0.203	252	262	198	241
95	Naive CD8+CD57+ cells	54	0.469	0.735	0.547	316	324	57	253.25
96	Non-classical monocytes	54	0.455	0.619	0.162	270	282	239	265.25
97	EM CD8+ cells	54	0.441	0.515	0.005	221	236	398	269
98	B-cells	54	0.444	0.541	0.019	230	255	381	274
99	TEMRA CD4+CD28+ cells	54	0.532	0.731	-0.276	312	322	154	275
100	TNF-α	62	0.470	0.729	0.286	318	318	150	276
101	EM CD8+CD57+ cells	54	0.532	0.731	-0.271	313	323	160	277.25
102	4-1BB	62	0.514	0.854	-1.279	369	358	19	278.75
103	Naive CD4+CD57+ cells	54	0.551	0.569	0.014	245	264	390	286
104	Tumor size	62	0.470	0.727	0.196	319	315	211	291
105	CM CD4+CD27-CD28- cells	54	0.491	0.920	1.146	382	382	20	291.5
106	TEMRA CD8+CD27+ cells	54	0.532	0.721	-0.156	305	314	244	292
107	TEMRA CD4+CD27+ cells	54	0.536	0.692	-0.088	291	303	299	296
108	IL-10	62	0.484	0.859	0.351	359	360	125	300.75
109	TEMRA CD4+CD27+CD28+ cells	54	0.532	0.728	-0.076	311	317	311	312.5
110	miR-17	62	0.471	0.735	0.100	320	325	288	313.25
111	EM CD4+CD28+ cells	54	0.533	0.717	-0.054	302	313	342	314.75
112	EM CD4+ cells	54	0.529	0.750	-0.098	321	329	290	315.25
113	CD56dimCD16+ NK-cells	54	0.535	0.702	-0.024	292	307	375	316.5
114	IL-27	62	0.482	0.834	0.157	350	352	243	323.75
115	miR-9	62	0.484	0.829	0.141	357	351	254	329.75
116	EM CD4+CD27+CD28+ cells	54	0.532	0.731	0.016	310	321	385	331.5
117	Naive CD4+CD27+CD28+ cells	54	0.526	0.772	-0.050	327	334	345	333.25
118	EM CD4+CD27+ cells	54	0.532	0.731	0.006	309	320	396	333.5
119	miR-326	62	0.501	0.992	-0.276	401	401	157	340
120	TEMRA CD8+CD27+CD28+ cells	54	0.508	0.938	-0.167	389	389	233	350
121	miR-21	62	0.488	0.898	0.095	375	374	293	354.25
122	Myeloid dendritic cells	54	0.512	0.900	0.075	374	376	313	359.25
123	TEMRA CD8+CD28+ cells	54	0.492	0.938	-0.104	390	390	284	363.5
124	Naive CD4+CD28+ cells	54	0.514	0.885	-0.043	367	368	352	363.5
125	Naive CD4+CD27+ cells	54	0.513	0.894	-0.049	372	372	347	365.75
126	miR-146a	62	0.503	0.974	0.114	398	397	279	368
127	IL-1β	62	0.509	0.923	0.074	386	383	317	368

128	Naive $CD4^+$ cells	54	0.513	0.894	-0.037	371	371	361	368.5
129	miR-150	62	0.504	0.968	-0.096	395	395	291	369
130	$CD56^{bright}CD16^-$ NK-cells	54	0.507	0.946	0.068	391	391	325	374.5
131	IGF-1	62	0.510	0.918	-0.023	381	381	376	379.75
132	miR-223	62	0.488	0.898	-0.008	376	375	394	380.25
133	IFN-γ	62	0.508	0.929	0.015	388	387	388	387.75
134	Naive $CD4^+CD27^-CD28^-$ cells	54	0.503	0.985	-0.024	400	400	374	393.5
